# A fraction of barrier-to-autointegration factor (BAF) associates with centromeres and controls mitosis progression

**DOI:** 10.1038/s42003-020-01182-y

**Published:** 2020-08-19

**Authors:** Mònica Torras-Llort, Sònia Medina-Giró, Paula Escudero-Ferruz, Zoltan Lipinszki, Olga Moreno-Moreno, Zoltan Karman, Marcin R. Przewloka, Fernando Azorín

**Affiliations:** 1 Institute of Molecular Biology of Barcelona, CSIC, Barcelona, Spain; 2 Institute for Research in Biomedicine (IRB Barcelona), The Barcelona Institute of Science and Technology, Barcelona, Spain; 3 MTA SZBK Lendület Laboratory of Cell Cycle Regulation and Institute of Biochemistry, Biological Research Centre, Szeged, Hungary; 4 Doctoral School of Biology, Faculty of Science and Informatics, University of Szeged, Szeged, Hungary; 5 School of Biological Sciences, Institute for Life Sciences, University of Southampton, Southampton, SO17 1BJ UK

**Keywords:** Cell biology, Chromatin, Chromosome segregation

## Abstract

Barrier-to-Autointegration Factor (BAF) is a conserved nuclear envelope (NE) component that binds chromatin and helps its anchoring to the NE. Cycles of phosphorylation and dephosphorylation control BAF function. Entering mitosis, phosphorylation releases BAF from chromatin and facilitates NE-disassembly. At mitotic exit, PP2A-mediated dephosphorylation restores chromatin binding and nucleates NE-reassembly. Here, we show that in *Drosophila* a small fraction of BAF (cenBAF) associates with centromeres. We also find that PP4 phosphatase, which is recruited to centromeres by CENP-C, prevents phosphorylation and release of cenBAF during mitosis. cenBAF is necessary for proper centromere assembly and accurate chromosome segregation, being critical for mitosis progression. Disrupting cenBAF localization prevents PP2A inactivation in mitosis compromising global BAF phosphorylation, which in turn leads to its persistent association with chromatin, delays anaphase onset and causes NE defects. These results suggest that, together with PP4 and CENP-C, cenBAF forms a centromere-based mechanism that controls chromosome segregation and mitosis progression.

## Abstract (web summary)

Torras-Llort et al. find that the chromatin binding protein Barrier-to-Autointegration Factor (BAF) associates with centromeres and is required for proper centromere assembly and function in *Drosophila*. They show that disruption of centromeric BAF localization prevents PP2A inactivation in mitosis compromising global BAF phosphorylation and leading to delayed anaphase onset and nuclear envelope defects.

## Introduction

Cell division involves major architectural rearrangements. Metazoa generally undergo open mitosis, which implies that the nuclear envelope (NE) disassembles at prometaphase and reassembles in telophase, after chromosome segregation is completed. A principal player in the regulation of NE dynamics during mitosis is barrier-to-autointegration factor (BAF)^1–7^. BAF is an essential 10 kDa chromatin-binding protein that is highly conserved in metazoan, being involved in multiple pathways including nuclear envelope reassembly (NER), chromatin epigenetics, DNA damage response, and defense against viral DNA infection (reviewed in ref. ^8^). Of great importance for its role in the regulation of NE dynamics, BAF interacts with the LEM-domain containing proteins LAP2, EMERIN, and MAN1^9–15^ that, together with lamins, form the nuclear lamina (reviewed in ref. ^16^). These interactions help anchoring chromatin to the NE in interphase and, in late mitosis, are essential for the recruitment of membranes to the ensemble of decondensing chromosomes^1,2,4^. A still poorly understood contribution of BAF to chromosome segregation has also been reported, since loss of BAF leads to strong chromosome segregation defects and high embryonic lethality in both *C. elegans* and *Drosophila*^1,2,17^.

Phosphorylation plays a key role in regulating BAF localization and function. The mitotic kinase VRK1/NHK1 phosphorylates BAF in mitosis and meiosis^1,18–20^. This phosphorylation weakens the binding of BAF to both chromatin and the LEM-domain proteins^21^, and is required for NE disassembly^1,22^. BAF plays also a crucial role in postmitotic NER^1–7^. At mitotic exit, BAF is dephosphorylated and reassociates with chromatin and the LEM-domain proteins, concentrating at the “core region” that surrounds the bulk of decondensing chromosomes, where its mobility and the mobility of the LEM-domain proteins decrease^3^, and nucleates NER. Two protein phosphatases, PP2A and PP4, have been shown to dephosphorylate BAF in different species^5,6,23^. In *C. elegans* and HeLa cells, PP2A is targeted to BAF by the LEM-domain protein Ankle2/LEM4, which is required for BAF dephosphorylation^5^. Ankle2/LEM4 also associates with VRK1/NHK1 and inhibits its activity, which enhances BAF dephosphorylation^5^. PP2A-mediated BAF dephosphorylation regulates BAF reassociation with chromatin at mitotic exit and is required for NER^5,6^. PP4 has also been shown to regulate BAF dephosphorylation during mitosis in HEK293 cells^23^.

Here we show that in *Drosophila* BAF is also a centromere-associated protein that is required for proper centromere assembly and function. Centromeric BAF (cenBAF) localization depends on the PP4 regulatory subunit Falafel (Flfl), which is recruited to centromeres by the constitutive centromeric protein CENP-C^24^. Our results suggest that, together with PP4/Flfl and CENP-C, cenBAF forms a centromeric network that controls phosphorylation and association with chromatin of the bulk of BAF, and regulates mitosis progression.

## Results

### BAF associates with centromeres

**Fig. 1:**
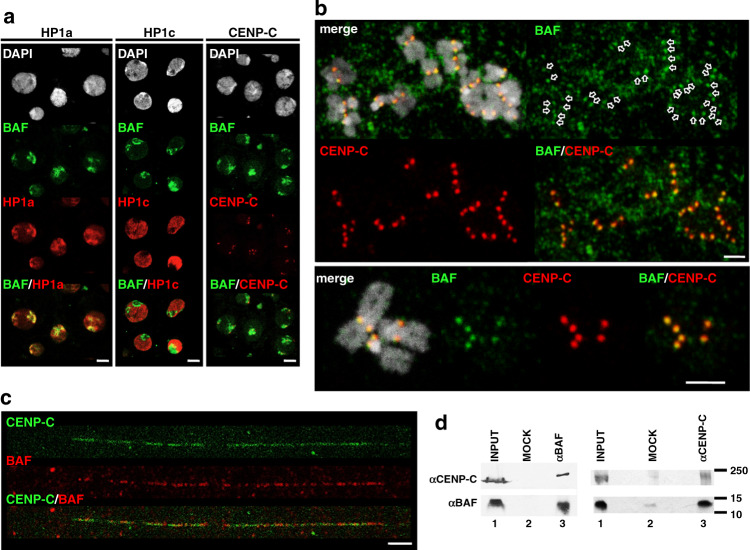
BAF associates with the centromere. **a** The patterns of immunolocalization with αBAF antibodies (green), and αHP1a (left), αHP1c (center), and αCENP-C (right) antibodies (red) are presented in interphase S2 cells. DNA was stained with DAPI. Scale bars correspond to 5 μm. **b** The patterns of immunolocalization with αBAF (green) and αCENP-C (red) antibodies are presented in metaphase chromosomes from S2 cells. Enlarged images are presented in the bottom. Arrows indicate αBAF signals that overlap with αCENP-C signals at centromeres. DNA was stained with DAPI. Scale bars correspond to 2.5 μm. **c** Immunostainings with αBAF (red) and αCENP-C (green) antibodies are presented in extended chromatin fibers prepared from S2 cells. Scale bar corresponds to 5 μm. **d** BAF/CENP-C co-immunoprecipitation. IPs were performed with αBAF or αCENP-C antibodies (lanes 3) and control preimmune serum (MOCK) (lanes 2) using S2 cells extracts. IP-materials were analyzed by WB using αCENP-C and αBAF antibodies. Lanes 1 correspond to 3% of the input material. The position of MW markers (in kDa) is indicated.

Others and we identified BAF amongst the proteins co-purifying with centromeric chromatin enriched in the histone H3 variant CENP-A^CID^^25^ (Supplementary Table 1) (see also “Methods” section). BAF also co-purified with canonical H3 containing chromatin^25^ and its binding to bulk chromatin has been reported^26,27^. Indeed, in interphase cells, we observed that BAF preferentially associated with heterochromatin, since it strongly co-localized with the heterochromatic HP1a variant (Fig. 1a, left), being largely excluded from regions enriched in the euchromatic HP1c isoform (Fig. 1a, center). However, unexpectedly, in metaphase chromosomes, BAF strongly overlapped with the constitutive centromeric protein CENP-C (Fig. 1b; see also Fig. 4d, e), suggesting that, in mitosis, chromosomal BAF localization was restricted to centromeres. The αBAF immunostaining detected in both interphase cells and metaphase chromosomes was specific, since it strongly decreased upon RNAi-mediated depletion of BAF (Supplementary Fig. 1). Chromatin fibers analysis confirmed centromeric localization of BAF since we detected BAF in ~80% of CENP-C-containing regions (*N* = 38) (one-tailed binomial test, *p*-value < 0.001) (Fig. 1c). This strong co-localization suggests that BAF localizes at centromeres also in interphase since mitotic cells accounted only for <5% of the total cells used in these analyses.

Co-immunoprecipitation experiments were consistent with the association of BAF with centromeres since αBAF antibodies immunoprecipitated CENP-C (Fig. 1d, left) and, vice versa, αCENP-C antibodies immunoprecipitated BAF (Fig. 1d, right). This interaction was resistant to treatment of the extract with DNAse I prior to immunoprecipitation (Supplementary Fig. 2a, b), suggesting that it was not mediated by chromatin binding. Along the same lines, we did not detect co-immunoprecipitation with the centromeric H3 variant CENP-A^CID^, which is an intrinsic structural component of the nucleosome and, hence, is tightly bound to centromeric chromatin. Endogenous CENP-A^CID^ was difficult to detect by WB in co-IP experiments. Thus, for these experiments, we used a stable S2 cell line expressing a CENP-A^CID^::YFP fusion protein that showed strong centromeric localization (Supplementary Fig. 2c). We observed that αBAF antibodies did not immunoprecipitate CENP-A^CID^::YFP (Supplementary Fig. 2d, lane 4) and, similarly, neither BAF nor CENP-C could be immunoprecipitated with αGFP (Supplementary Fig. 2d, lane 3). The lack of CENP-C co-immunoprecipitation with CENP-A^CID^ has been previously reported^28^.

Altogether these results suggest that a fraction of BAF (cenBAF) stays associated with centromeres throughout the cell cycle.

### BAF is required for functional centromere assembly

**Fig. 2:**
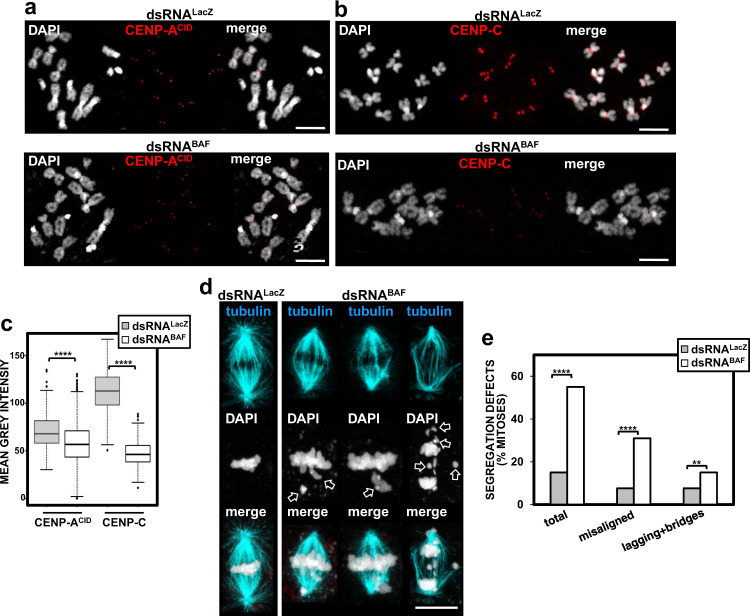
BAF depletion impairs centromere assembly and chromosome segregation. **a** and **b** The patterns of immunolocalization with αCENP-A^CID^ **a** and αCENP-C **b** antibodies (red) are presented in metaphase chromosomes from S2 cells upon RNAi-mediated depletion of BAF (dsRNA^BAF^) and in control cells treated with dsRNA against LacZ (dsRNA^LacZ^). DNA was stained with DAPI. Scale bars correspond to 5 μm. **c** Quantitative analyses of the results shown in **a** and **b**. The mean grey values per centromere of αCENP-A^CID^ and αCENP-C fluorescence are shown for dsRNA^BAF^ and control dsRNA^LacZ^ cells. Values correspond to a representative experiment out of five independent experiments showing equivalent results (*N* > 382; Kruskal–Wallis test, ****p-value < 0.0001. **d** Metaphase figures from dsRNA^BAF^ and control dsRNA^LacZ^ cells. The spindle was stained with αTubulin antibodies (blue). DNA was stained with DAPI. Arrows indicate chromosome segregation defects (metaphase misalignment, lagging and fragmented chromosomes, and chromatin bridges). Scale bar corresponds to 5 μm. **e** The percentages of mitoses showing segregation defects are presented for dsRNA^BAF^ and control dsRNA^LacZ^ cells. Results are presented for total defects, misaligned metaphase chromosomes, and anaphase chromatin bridges and lagging chromosomes. Values are the sum of two independent experiments showing equivalent results (*N* > 104; two-tailed Fisher’s test, **p < 0.01, ****p < 0.0001).

We observed that depletion of BAF decreased centromeric CENP-C and CENP-A^CID^ levels in both metaphase chromosomes (Fig. 2a–c) and interphase cells (Supplementary Fig. 3a, b). We observed that reduction of CENP-A^CID^ levels was markedly weaker than that observed for CENP-C (Fig. 2c and Supplementary Fig. 3b), suggesting that BAF primarily affects centromeric CENP-C levels. Along the same lines, we observed that expression of a BAF::YFP construct decreased CENP-C levels, without significantly affecting centromeric CENP-A^CID^ levels (Kruskal–Wallis test, *p*-value = 0.215) (Supplementary Fig. 3c, d). Localization of BAF::YFP largely mimicked the pattern of immunolocalization of endogenous BAF since, in interphase cells, BAF::YFP overlapped with HP1a (Supplementary Fig. 4a), while it localized at centromeric regions in metaphase chromosomes (Supplementary Fig. 4b, c) and strongly overlapped with CENP-C in chromatin fibers (64%; *N* = 37) (one-tailed binomial test, *p*-value < 0.05) (Supplementary Fig. 4d). However, in contrast to endogenous BAF, BAF::YFP localization in metaphase chromosomes extended to pericentromeric heterochromatin (Supplementary Fig. 4c). Genetic analysis showed that BAF::YFP acts as a dominant negative mutation. In these experiments, *baf*^*RNAi*^ knockdown flies, which carry a UAS_GAL4_-construct expressing a synthetic hairpin from the *baf*-coding region, were crossed to *nub*-GAL4 flies to specifically induced BAF depletion in the pouch region of wing imaginal disks. BAF depletion resulted in a strong wing phenotype in adult flies (Supplementary Fig. 3e, bottom left panel). This phenotype was partially rescued by expression of RNAi-resistant untagged BAF^R^ (Supplementary Fig. 3e, bottom center panel), but not by expression of an RNAi-resistant BAF::YFP^R^ construct (Supplementary Fig. 3e, bottom right panel). Moreover, while overexpression of untagged BAF caused no detectable wing defects (Supplementary Fig. 3e, top center panel), overexpression of BAF::YFP in control wild-type flies mimicked the loss-of-function phenotype observed upon BAF depletion (Supplementary Fig. 3e, top right panel). In this regard, we observed that expression of BAF::YFP strongly reduced the levels of endogenous BAF, whereas several other chromosomal proteins were not affected (i.e., CENP-C, CENP-A^CID^, HP1a) (Supplementary Fig. 3f). On the other hand, as discussed below, BAF::YFP showed an aberrant pattern of phosphorylation (Supplementary Fig. 5b). Impaired phosphorylation is likely affecting BAF::YFP function since phosphorylation regulates binding of BAF to both chromatin and the LEM-domain proteins^21^. Hence, the negative dominant character of BAF::YFP is likely associated with destabilization and replacement of endogenous BAF by a non-functional BAF::YFP form of altered chromatin-binding dynamics due to abnormal phosphorylation.

CENP-A^CID^ and CENP-C are conserved constitutive centromeric proteins that are essential for centromere/kinetochore assembly and chromosome segregation (reviewed in ref. ^29^). Hence, their decrease upon BAF depletion suggest a contribution of BAF to centromere function. Consistent with this hypothesis, depletion of BAF increased the frequency of chromosome segregation defects (Fig. 2d, e). In particular, the frequency of mitoses showing misaligned metaphase chromosomes strongly increased from ~7% in control dsRNA^LacZ^ cells to ~31% in BAF-depleted cells (Fig. 2e). The frequency of anaphase chromatin bridges and lagging chromosomes also increased, though to a lesser extent (Fig. 2e). Expression of the dominant negative BAF::YFP form also induced strong segregation defects (~86%) (*N* = 14; two-tailed Fisher’s test, *p*-value < 0.0001) (Supplementary Fig. 3g). These results are in agreement with previous reports showing a high incidence of chromosome segregation defects upon BAF depletion in *C. elegans*^1,2^ and in *baf* null mutant flies^17^.

Altogether these observations suggest that BAF stabilizes centromeric association of the essential centromeric components CENP-A^CID^ and CENP-C and, thus, it is required for accurate chromosome segregation.

### cenBAF localization depends on PP4 phosphatase

It has been shown that BAF is phosphorylated at mitosis by the mitotic kinase VRK1/NHK1^1,5,19,20^. Phos-tag gel electrophoretic analyses confirmed BAF phosphorylation since we detected mono- (1pBAF) and di-phosphorylated (2pBAF) BAF species that migrated slower than non-phosphorylated BAF and were sensitive to treatment with alkaline phosphatase (AP) (Supplementary Fig. 5a). VRK1/NHK1 overexpression increased the proportion of 2pBAF, whereas VRK1/NHK1 depletion increased non-phosphorylated BAF (Supplementary Fig. 5a). Previous studies showed that, at mitosis, phosphorylation resolves the interaction of BAF with chromatin as well as with the NE LEM-domain proteins^1,5,19,20^. In this regard, we observed that mitotic spreads had high non-chromosomal αBAF reactivity (Fig. 1b and Supplementary Fig. 1b, top panel), which likely reflects the bulk of free pBAF that exists during mitosis since this background was strongly reduced upon BAF depletion (Supplementary Fig. 1b, bottom panel). Noteworthy, the dominant negative BAF::YFP form showed an aberrant phosphorylation pattern (Supplementary Fig. 5b), suggesting that it was not properly phosphorylated by VRK1/NHK1. Impaired VRK1/NHK1 phosphorylation is likely responsible for the persistent binding of BAF::YFP to heterochromatin observed in mitosis (Supplementary Fig. 4c).

**Fig. 3:**
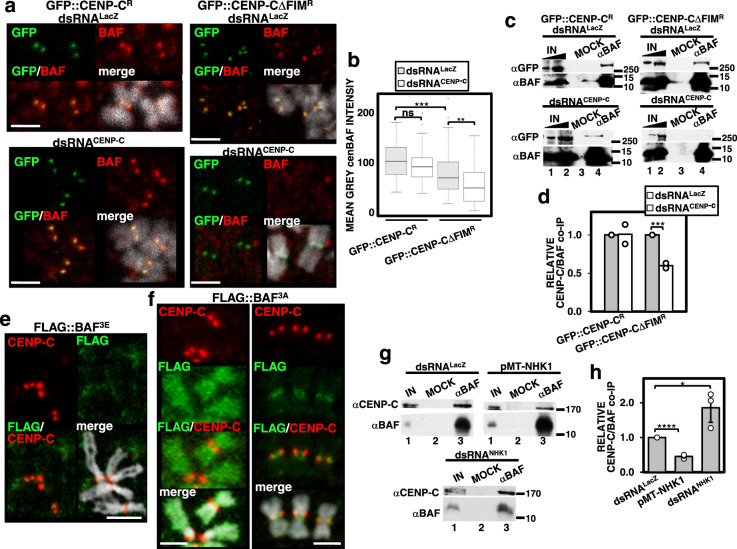
Centromeric cenBAF localization depends on Flfl. **a** Immunostainings with αBAF antibodies (red) are presented in cells expressing GFP::CENP-C^R^ (left) and GFP::CENP-CΔFIM^R^ (right) upon CENP-C depletion (dsRNA^CENP-C^) and in control dsRNA^LacZ^ cells. GFP signals are direct fluorescence. DNA was stained with DAPI. Scale bars are 2.5 μm. **b** Quantitative analysis of the results shown in **a**. Mean grey values per centromere of αBAF fluorescence are presented for control dsRNA^LacZ^ and dsRNA^CENP-C^ cells expressing the indicated constructs. Values correspond to a representative experiment out of five independent experiments showing equivalent results (*N* > 48; Kruskal–Wallis test, *p*-value: ns > 0.05, **p < 0.01, ***p < 0.001). **c** co-IP experiments with αBAF antibodies in extracts from control dsRNA^LacZ^ and dsRNA^CENP-C^ cells expressing GFP::CENP-C^R^ (left) and GFP::CENP-CΔFIM^R^ (right) (lanes 4). Lanes 3 are mock IPs with preimmune serum. Lanes 1 and 2 are 2% and 5% of the input, respectively. IPs were analyzed by WB using αGFP and αBAF antibodies. The position of MW markers (in kDa) is indicated. **d** Quantitative analysis of the results shown in **c**. The ratio of αCENP-C and αBAF signals normalized with respect to the corresponding control dsRNA^LacZ^ cells is presented for dsRNA^CENP-C^ cells expressing the indicated constructs. Results are the average of two independent experiments (two-tailed *t*-test, ***p-value < 0.001). **e** Immunostaining with αFLAG antibodies (green) in metaphase chromosomes from cells transiently expressing FLAG::BAF^3E^. Immunostaining with αCENP-C antibodies (red) is also presented. DNA was stained with DAPI. Scale bar is 5 μm. **f** As in **e** but for cells transiently expressing FLAG::BAF^3A^. **g** co-IP experiments with αBAF antibodies in extracts from control dsRNA^LacZ^ cells, VRK1/NHK1-depleted dsRNA^NHK1^ cells and pMT-NHK1 cells overexpressing VRK1/NHK1 (lanes 3). Lanes 2 are mock IPs with preimmune serum. Lanes 1 correspond to 3% of the input. IPs were analyzed by WB using αCENP-C and αBAF antibodies. The position of MW markers (in kDa) is indicated. **h** Quantitative analyses of the results shown in **g**. The ratio of αCENP-C and αBAF signals normalized respect to control dsRNA^LacZ^ cells is presented for pMT-NHK1 and dsRNA^NHK1^ cells. Results are the average of three independent experiments (error bars are SD; two-tailed *t*-test, *p < 0.05, ****p < 0.0001).

In light of these observations, we hypothesized that the fraction of BAF that remained bound to centromeres during mitosis was likely non-phosphorylated. In this regard, protein phosphatase 4 (PP4), which has been previously shown to regulate BAF phosphorylation^23^, is known to associate with centromeres through the interaction of its conserved regulatory 3 subunit, Flfl, with CENP-C^24^. A short sequence-motif in CENP-C (Falafel Interacting Motif (FIM)) mediates this interaction^24^. Notably, deletion of this motif, which disrupts binding of PP4 to centromeres^24^, strongly reduced the levels of centromeric BAF (cenBAF). In these experiments, stable cell lines expressing RNAi-resistant GFP-tagged CENP-C constructs, lacking the FIM motif (GFP::CENP-CΔFIM^R^) or not (GFP::CENP-C^R^), were subjected to depletion of endogenous CENP-C that did not affect centromeric localization of the RNAi-resistant constructs (Fig. 3a). Upon depletion of endogenous CENP-C, Flfl recruitment to centromeres was impaired in cells expressing GFP::CENP-CΔFIM^R^^24^ (Supplementary Fig. 6a, center and b) and, concomitantly, we observed that cenBAF levels were strongly reduced as determined by both IF (Fig. 3a, b) and co-IP experiments (Fig. 3c, d). As expected, in control GFP::CENP-C^R^-expressing cells, depletion of endogenous CENP-C did not affect Flfl recruitment to centromeres (two-tailed Fischer’s test, *p*-value = 0.58) (Supplementary Fig. 6a, left and b) and, consequently, cenBAF levels were not significantly affected (Fig. 3a–d). In addition, we observed that Flfl depletion decreased cenBAF levels (Supplementary Fig. 7c–f), without affecting total BAF levels (Supplementary Fig. 7a, b). These results suggest that PP4 is required for centromeric localization of cenBAF.

We also analyzed localization of FLAG::BAF phosphomutants, in which the three putative VRK1/NHK1 phosphorylation sites (S2, T4, and S5)^18–20,23^ were mutated to A (phosphonull) and E (phosphomimetic) (Supplementary Fig. 5c, d). As expected, we observed that the phosphomimetic FLAG::BAF3E construct was not binding chromosomes (*N* = 238) (Fig. 3e), while the phosphonull FLAG::BAF3A mutant showed persistent binding to chromosomes in mitosis (Fig. 3f). Noteworthy, although FLAG::BAF3A was generally binding across the entire chromosome (Fig. 3f, left), we observed that its localization was restricted to centromeres in ~15% of the mitoses (*N* = 123) (two-tailed binomial test, *p*-value < 0.0001) (Fig. 3f, right), which is consistent with cenBAF not being phosphorylated. In addition, BAF phosphorylation impaired BAF/CENP-C co-immunoprecipitation, since VRK1/NHK1 overexpression decreased co-immunoprecipitation of CENP-C with αBAF, whereas VRK1/NHK1 depletion increased it (Fig. 3g, h). Altogether these results suggest that, during mitosis, cenBAF is maintained non-phosphorylated by the action of PP4.

### cenBAF prevents PP2A-dependent BAF dephosphorylation

**Fig. 4:**
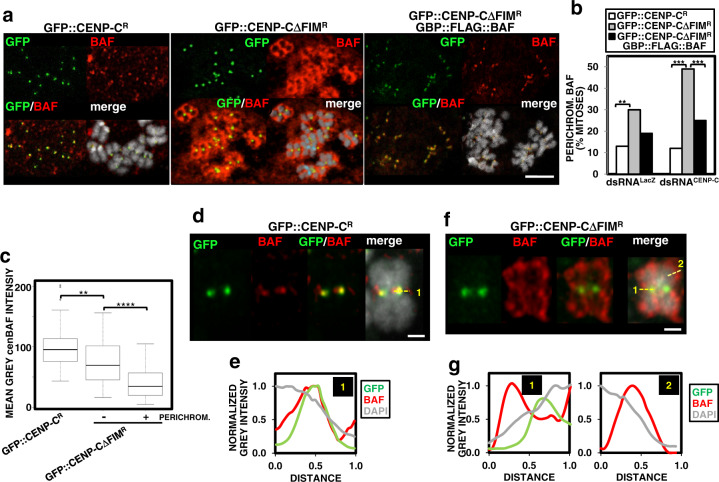
cenBAF prevents the accumulation of perichromosomal BAF in mitosis. **a** The patterns of immunolocalization with αBAF antibodies (red) are presented in dsRNA^CENP-C^ cells expressing the indicated constructs. GFP signals are direct fluorescence (green). DNA was stained with DAPI. Scale bar corresponds to 5 μm. **b** The percentages of mitoses showing perichromosomal BAF are presented for control dsRNA^LacZ^ and dsRNA^CENP-C^ cells expressing the indicated constructs. Values correspond to the sum of 3-4 independent experiments showing equivalent results (*N* > 73; two-tailed Fischer’s test, **p < 0.01, ***p < 0.001). **c** The mean grey values per centromere of αBAF fluorescence are presented for dsRNA^CENP-C^ cells expressing GFP::CENP-C^R^ (left) and dsRNA^CENP-C^ cells expressing GFP::CENP-CΔFIM^R^ showing perichromosomal BAF (right) or not (center). Values correspond to a representative experiment out of five independent experiments showing equivalent results (*N* > 33; Kruskal–Wallis test, **p < 0.01, ****p < 0.0001). **d** Super-resolution microscopy analysis of a representative chromosome from dsRNA^CENP-C^ cells expressing GFP::CENP-C^R^. The pattern of immunolocalization with αBAF antibodies is shown in red. GFP signals are direct fluorescence (green). DNA was stained with DAPI. Scale bar corresponds to 1 μm. **e** The profiles of αBAF (red), GFP (green), and DAPI (light grey) fluorescence along the line indicated in **d** are presented. Distance increases from left to right. **f** As in **d** but for a representative chromosome from dsRNA^CENP-C^ cells expressing GFP::CENP-CΔFIM^R^ showing perichromosomal BAF. **g** The profiles of αBAF (red), GFP (green), and DAPI (light grey) fluorescence along the lines indicated in **f** are presented. Distance increases from left to right.

Normally, in mitosis, the bulk of BAF stays phosphorylated and free in the cytoplasm, reassociating with chromatin only after chromosomes start to decondense in telophase, an event that marks the formation of the “core region” and the initiation of NER^3,5–7^. However, we observed that, concomitant to decreased cenBAF, CENP-C depletion in GFP::CENP-CΔFIM^R^-expressing cells induced intense perichromosomal αBAF immunostaining in ~50% of the mitosis, which was infrequent when CENP-C depletion was performed in control GFP::CENP-C^R^-expressing cells (Fig. 4a, left and center, and 4b). WB analysis showed that total BAF levels did not change upon CENP-C depletion in both GFP::CENP-CΔFIM^R^-expressing cells and control GFP::CENP-C^R^-expressing cells (Supplementary Fig. 8). The presence of perichromosomal BAF correlated with the reduction of cenBAF since, in CENP-C-depleted GFP::CENP-CΔFIM^R^-expressing cells, mitoses showing perichromosomal αBAF immunostaining had lower cenBAF levels than mitoses without perichromosomal BAF (Fig. 4c). We also observed that, in comparison to control GFP::CENP-C^R^-expressing cells, cells expressing GFP::CENP-CΔFIM^R^ had reduced cenBAF levels even without depletion of endogenous CENP-C in control dsRNA^LacZ^ cells (Fig. 3a, b) and, concomitantly, they showed increased perichromosomal BAF (Fig. 4b). Super-resolution microscopy analysis confirmed these results. In control CENP-C-depleted GFP::CENP-C^R^-expressing cells, the distribution of BAF was largely restricted to the centromere, showing a well-defined maximum that strongly overlapped with GFP::CENP-C (Fig. 4d, e). Instead, in CENP-C-depleted GFP::CENP-CΔFIM^R^-expressing cells, the distribution of BAF was radically different with the maximum in the perichromosomal layer surrounding the chromosome and a much-reduced overlapping with CENP-C at the centromere (Fig. 4f, g). Within this perichromosomal layer, BAF was in direct association with the chromosome, though the maximum BAF concentration was detected beyond its surface (Fig. 4g).

Results reported above suggest that disrupting cenBAF localization induces the accumulation of BAF in a perichromosomal layer that wraps around chromosomes in mitosis. This phenotype depends on cenBAF since constitutive targeting of BAF to centromeres prevents the accumulation of perichromosomal BAF. For these experiments, we used a GBP::FLAG::BAF construct that was tethered to centromeres by specifically recognizing the GFP-moiety of the GFP::CENP-C^R^ and GFP::CENP-CΔFIM^R^ constructs via the GFP-binding protein (GBP)^30^ (Supplementary Fig. 9a, b). Importantly, expression of GBP::FLAG::BAF in control GFP::CENP-C^R^-expressing cells caused no detectable defects (Supplementary Fig. 9c–h). Notably, we observed that expression of GBP::FLAG::BAF in CENP-C-depleted GFP::CENP-CΔFIM^R^-expressing cells strongly reduced perichromosomal BAF (Fig. 4a, right and b). Expression of GBP::FLAG::BAF also tended to reduce perichromosomal BAF in control dsRNA^LacZ^ GFP::CENP-CΔFIM^R^-expressing cells (two-tailed Fischer’s test, *p*-value = 0.07) (Fig. 4b).

**Fig. 5:**
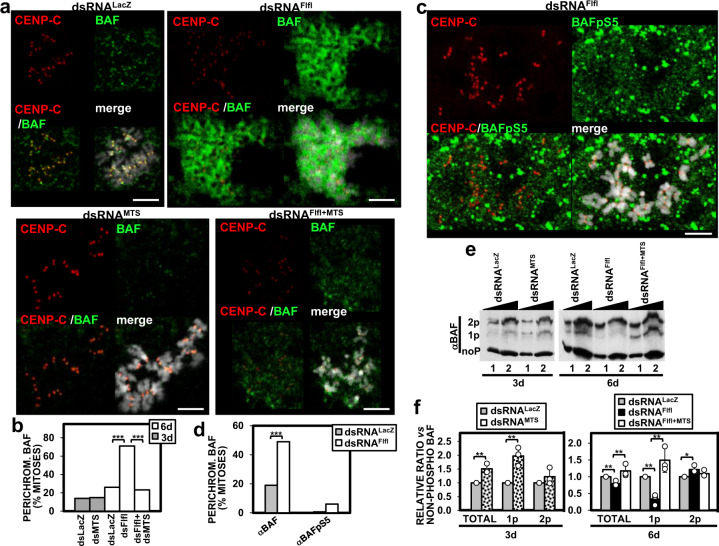
Perichromosomal BAF requires PP2A-mediated dephosphorylation of pBAF. **a** The patterns of immunolocalization with αBAF (green) and αCENP-C (red) antibodies are presented for control dsRNA^LacZ^ cells and cells treated with dsRNA against Flfl (dsRNA^Flfl^), MTS (dsRNA^MTS^), and both Flfl and MTS (dsRNA^Flfl+MTS^). DNA was stained with DAPI. Scale bars correspond to 5 μm. **b** The percentages of mitoses showing perichromosomal BAF are presented for the indicated RNAi-treated cells. Depletion was carried out for 3 or 6 days as indicated. Values are the sum of 5–9 independent experiments showing equivalent results (*N* > 165; two-tailed Fischer’s test, ****p < 0.0001). **c** The patterns of immunolocalization with αBAFpS5 (green) and αCENP-C (red) antibodies are presented for cells treated with dsRNA against Flfl (dsRNA^Flfl^). DNA was stained with DAPI. Scale bar corresponds to 5 μm. **d** The percentages of mitoses showing perichromosomal αBAF and αBAFpS5 signals in control dsRNA^LacZ^ and dsRNA^Flfl^ cells are compared. Values are the sum of three independent experiments showing equivalent results (*N* > 86 for αBAF and *N* > 28 for αBAFpS5; two-tailed Fischer’s test, ***p < 0.001). **e** The pattern of BAF phosphorylation is analyzed by phos-tag gel electrophoresis of increasing amounts of extracts (lanes 1 and 2) prepared from control dsRNA^LacZ^ cells and cells treated with dsRNA against Flfl (dsRNA^Flfl^), MTS (dsRNA^MTS^), and both Flfl and MTS (dsRNA^Flfl+MTS^). Extracts are analyzed by WB using αBAF antibodies. The positions corresponding to non-phosphorylated (noP), and mono- (1pBAF), and di-phosphorylated (2pBAF) species are indicated. **f** Quantitative analysis of the results shown in **e**. The relative proportions with respect to non-phosphorylated BAF of total pBAF, and 1pBAF and 2pBAF species are presented for the indicated RNAi-treated cells. Results are the average of three independent experiments (error bars are SD; two-tailed *t*-test, *p < 0.05, **p < 0.01).

The association of BAF with chromatin is regulated by PP2A phosphatase that, at mitotic exit, dephosphorylates free pBAF and restores its binding to chromatin^1,5,6,20^. In this regard, we observed that formation of the perichromosomal BAF layer depended on PP2A. In these experiments, we used Flfl-depleted cells that, similar to CENP-C-depleted GFP::CENP-CΔFIM^R^-expressing cells, showed impaired cenBAF localization (Supplementary Fig. 7c–f) and, consequently, high perichromosomal BAF (Fig. 5a, b). Depletion of the *Drosophila* PP2A catalytic subunit Microtubule star (MTS) in Flfl-depleted cells strongly reduced perichromosomal BAF (Fig. 5a, b), while, on the other hand, MTS depletion alone did not induce perichromosomal BAF (two-tailed Fischer’s test, *p*-value = 0.86) (Fig. 5a, b). Note that MTS depletion was carried out for only 3 days since longer depletion times resulted in high cell death. In Flfl-depleted cells, MTS depletion was carried out simultaneously to Flfl-depletion during the last 3 days of the 6 days of treatment with dsRNA^Flfl^ (see “Methods“ section). The efficiency of the knockdowns was confirmed by WB (Supplementary Fig. 10). Altogether these results suggest that the accumulation of perichromosomal BAF observed when cenBAF localization is impaired involves dephosphorylation of free pBAF by PP2A in mitosis. Consistent with this, perichromosomal BAF was not reactive with an αBAFpS5 antibody (Fig. 5c, d), which specifically recognized pBAF (Supplementary Fig. 5e) (see “Methods” section).

Phos-tag gel electrophoresis analysis confirmed the role of PP2A in BAF dephosphorylation since MTS depletion significantly increased total pBAF levels (Fig. 5e, f). Interestingly, this effect was mainly constrained to 1pBAF (Fig. 5e, f), since the levels of 2pBAF were not significantly affected (two-tailed *t*-test, *p*-value = 0.268) (Fig. 5e, f). These results suggest that PP2A preferentially dephosphorylates 1pBAF. Conversely, and opposite to what would be expected, Flfl depletion decreased total pBAF levels (Fig. 5e, f), strongly reducing 1pBAF levels (Fig. 5e, f), which suggests that Flfl depletion enhanced PP2A-mediated dephosphorylation of 1pBAF. Consistent with this, depletion of MTS in Flfl-depleted cells restored 1pBAF levels (Fig. 5e, f). We also observed that Flfl depletion slightly increased 2pBAF levels (Fig. 5e, f).

### cenBAF regulates progression through mitosis

**Fig. 6:**
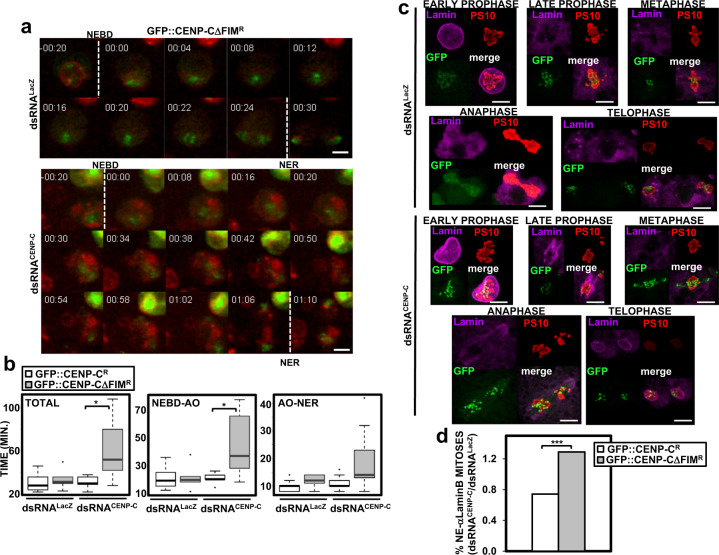
cenBAF regulates mitosis progression. **a** In vivo time-lapse recordings of control dsRNA^LacZ^ (top) and dsRNA^CENP-C^ (bottom) Nup-107::mRFP cells expressing GFP::CENP-CΔFIM^R^. mRFP (red) and GFP (green) signals are direct fluorescence. NEBD and NER are indicated. Times are minutes before/after NEBD. Scale bars correspond to 5 μm. **b** Quantitative analysis of the results shown in **a**. The total duration of mitosis (left), and the times from NEBD to anaphase onset (AO) (center) and from AO to NER (right) are presented for control dsRNA^LacZ^ and dsRNA^CENP-C^ Nup-107::mRFP cells expressing the indicated constructs (*n* = 3; *N* > 5; Kruskal–Wallis test, *p < 0.05). **c** Immunostainings with αLaminB antibodies (magenta) and αPS10 (red) of control dsRNA^LacZ^ and CENP-C-depleted dsRNA^CENP-C^ cells expressing GFP::CENP-CΔFIM^R^. Mitotic phases are indicated. GFP (green) is direct fluorescence. Scale bars correspond to 5 μm. **d** The effect of CENP-C depletion on the proportion of mitoses showing NE-assembled αLaminB immunostaining is shown with respect to control dsRNA^LacZ^ cells for GFP::CENP-C^R^ and GFP::CENP-CΔFIM^R^-expressing cells. Values are the sum of 5–8 independent experiments showing equivalent results (*N* > 92; Chi-square test, ***p < 0.001).

Next, we analyzed the effects of disrupting cenBAF localization on mitosis progression. For this purpose, we performed live image analysis in cells expressing the nuclear pore component Nup-107::mRFP to label the NE. We observed that, in comparison to control dsRNA^LacZ^ cells, depletion of CENP-C in GFP::CENP-CΔFIM^R^-expressing cells significantly increased the overall duration of mitosis (Fig. 6a, b, and Supplementary Movies 1 and 2). No such effect was observed in GFP::CENP-C^R^-expressing cells, where the length of mitosis was similar in control dsRNA^LacZ^ cells and after CENP-C depletion (Fig. 6b). These results suggest that impairing cenBAF localization delays mitosis progression. In particular, the time from NEBD to AO was significantly increased (Fig. 6a, b, and Supplementary Movies 1 and 2). The time from AO to NER also showed a clear tendency to increase (Kruskal–Wallis test, *p*-value = 0.057) (Fig. 6a, b, and Supplementary Movies 1 and 2).

In CENP-C-depleted GFP::CENP-CΔFIM^R^-expressing cells, we often observed that Nup-107::mRFP signal persisted through mitosis (Supplementary Movies 2–4), suggesting incomplete NE disassembly. Nuclear pore disassembly is a very early step in NEBD. Thus, to further analyze the effects on NE assembly, we performed IF experiments using αPS10 antibodies, which recognize H3S10P in mitotic cells, and αLaminB antibodies to monitor NE status. In control dsRNA^LacZ^ cells, αLaminB immunostaining marked the NE in interphase, became diffuse through the cytoplasm after NEBD in late prophase to relocate to the NE during NER in telophase (Fig. 6c, top). However, in CENP-C-depleted GFP::CENP-CΔFIM^R^-expressing cells, we often detected less diffuse αLaminB immunostaining in late prophase (Fig. 6c, bottom). Moreover, the proportion of αPS10-positive cells showing NE-assembled αLaminB immunostaining increased upon CENP-C depletion in GFP::CENP-CΔFIM^R^-expressing cells in comparison with control GFP::CENP-C^R^-expressing cells (Fig. 6d). Altogether these results suggest that, upon disrupting cenBAF localization, the NE remains partially assembled during mitosis.

**Fig. 7:**
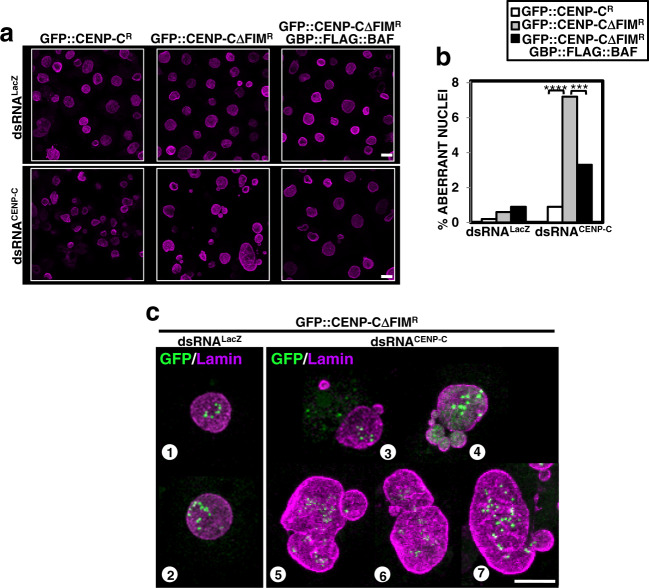
Disrupting cenBAF localization causes NE morphology defects. **a** Immunostainings with αLaminB antibodies (magenta) of control dsRNA^LacZ^ and CENP-C-depleted dsRNA^CENP-C^ cells expressing the indicated constructs. Scale bars correspond to 10 μm. **b** Quantitative analysis of the results are shown in **a**. The percentage of cells showing altered nuclear morphology is presented for control dsRNA^LacZ^ and dsRNA^CENP-C^ cells expressing the indicated constructs. Values are the sum of 3–4 independent experiments showing equivalent results (*N* > 334; two-tailed Fischer’s test, ***p < 0.001, ****p < 0.0001). **c** Enlarged images of immunostainings with αLaminB antibodies (magenta) of dsRNA^CENP-C^ cells (images 3–7) and control dsRNA^LacZ^ cells (images 1 and 2) expressing GFP::CENP-CΔFIM^R^. GFP signals are direct fluorescence. Scale bar corresponds to 5 μm.

In addition, in comparison to control GFP::CENP-C^R^-expressing cells, we observed an increased frequency of NE morphology defects in CENP-C-depleted GFP::CENP-CΔFIM^R^-expressing cells (two-tailed Fischer’s test, *p*-value < 0.0001) (Fig. 7a, b). These defects ranged from nuclear budding and the formation of micronuclei (Fig. 7c, image 3), to multinucleated cells (Fig. 7c, image 4) and cells with enlarged nucleus of irregular NE (Fig. 7c, images 5–7). In this regard, in CENP-C-depleted GFP::CENP-CΔFIM^R^-expressing cells, we detected aberrant mitoses that generated cells with abnormal nuclear morphology, often multinucleated (Supplementary Movies 3–6). Notably, these defects were significantly rescued when BAF was constitutively targeted to centromeres in cells expressing GBP::FLAG::BAF (Fig. 7a, b).

### cenBAF, CENP-C, and PP4 localization is interdependent

**Fig. 8:**
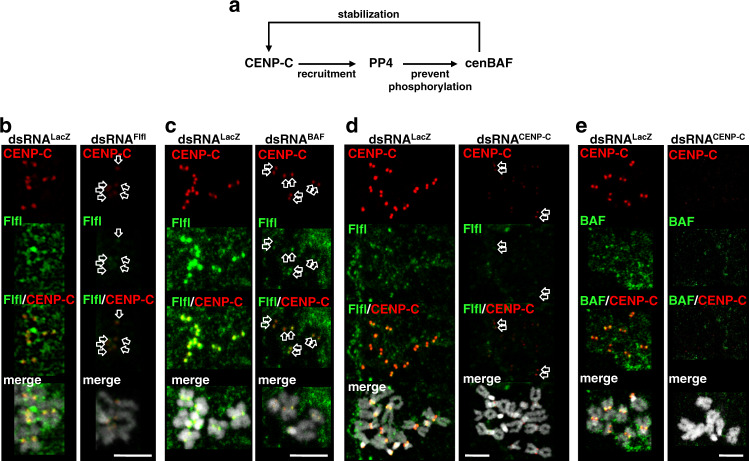
cenBAF, PP4, and CENP-C are interdependent for centromeric localization. **a** Schematic representation of the interdependences for centromeric localization between cenBAF, PP4, and CENP-C. CENP-C mediates centromeric recruitment of PP4, which is required to retain cenBAF at centromeres during mitosis. cenBAF stabilizes CENP-C at centromeres. **b** Immunostainings with αFlfl (green) and αCENP-C antibodies (red) are presented for mitotic chromosomes from dsRNA^Flfl^ (right) and control dsRNA^LacZ^ (left) cells. DNA was stained with DAPI. Arrows in dsRNA^Flfl^ cells indicate centromeric αCENP-C signals. Scale bar corresponds to 5 μm. **c** Immunostainings with αFlfl (green) and αCENP-C antibodies (red) are presented for mitotic chromosomes from dsRNA^BAF^ (right) and control dsRNA^LacZ^ (left) cells. DNA was stained with DAPI. Arrows indicate αFlfl signals in dsRNA^BAF^ cells overlapping with αCENP-C at centromeres. Scale bar corresponds to 5 μm. **d** Immunostainings with αFlfl (green) and αCENP-C antibodies (red) are presented for mitotic chromosomes from dsRNA^CENP-C^ (right) and control dsRNA^LacZ^ (left) cells. DNA was stained with DAPI. Scale bar corresponds to 5 μm. **e** Immunostainings with αBAF (green) and αCENP-C antibodies (red) are presented for mitotic chromosomes from dsRNA^CENP-C^ (right) and control dsRNA^LacZ^ (left) cells. DNA was stained with DAPI. Scale bar corresponds to 5 μm.

Results reported above suggest a model by which CENP-C mediates recruitment of PP4 to centromeres, PP4 retains cenBAF at centromeres in mitosis, which in turn stabilizes CENP-C (Fig. 8a). Consistent with these interdependences, Flfl depletion, which reduced centromeric cenBAF levels (Supplementary Fig. 7c–f), also decreased centromeric CENP-C (Fig. 8b) (see also Supplementary Fig. 7c, bottom panel), and, along with CENP-C, BAF depletion reduced centromeric levels of Flfl (Fig. 8c). Moreover, depletion of CENP-C abolished centromeric localization of both Flfl (Fig. 8d) and cenBAF (Fig. 8e). Noteworthy, like when cenBAF localization is disturbed in CENP-C-depleted GFP::CENP-CΔFIM^R^-expressing cells (Fig. 4a) or upon Flfl-depletion (Fig. 5a), depletion of CENP-C induced the accumulation of perichromosomal BAF too (Supplementary Fig. 11). Altogether these results indicate that CENP-C, PP4, and BAF are interdependent for centromeric localization in mitotic chromosomes. Interestingly, we also observed that targeting of GBP::FLAG::BAF to centromeres in CENP-C-depleted GFP::CENP-CΔFIM^R^-expressing cells significantly rescued centromeric Flfl levels (Supplementary Fig. 6a, b), without affecting depletion of endogenous CENP-C (Supplementary Fig. 6c), suggesting that BAF also stabilizes centromeric PP4 independently of CENP-C.

## Discussion

Here we have unveiled a novel centromere-based mechanism that controls mitosis progression. Central to this mechanism is the NE component BAF. We have shown that a fraction of BAF (cenBAF) associates with centromeres. BAF is known to bind across chromatin in interphase^26,27^, but, in mitosis, VRK1/NHK1 phosphorylates BAF^1,18–20^, resulting in its release from chromatin. Our results suggest that, at the centromere, PP4 prevents phosphorylation and release of cenBAF in mitosis. cenBAF is a very small proportion of total BAF. In this regard, the vast majority of BAF is phosphorylated and free in mitosis, resulting in high non-chromosomal background that likely precluded the identification of cenBAF in previous IF studies.

cenBAF forms a functional network with PP4 and CENP-C, as all three factors are interdependent for their centromeric localization. Whether they physically interact to form a centromeric complex remains to be determined. In favor of this possibility, CENP-C interacts directly with Flfl in vitro^24^ and, moreover, BAF and CENP-C co-immunoprecipitate, suggesting that, either directly or indirectly, CENP-C also interacts with BAF. Along the same lines, constitutive targeting of BAF to centromeres stabilizes centromeric Flfl as well as CENP-C.

Our results suggest that cenBAF stabilizes CENP-C at centromeres and, thus, it is required for accurate chromosome segregation. CENP-C connects centromeric chromatin with the outer kinetochore^31,32^ and loss-of-function mutations induce strong chromosome segregation defects, mostly chromosome misalignment in metaphase^33,34^. Interestingly, metaphase misalignment is the most frequent chromosome segregation defect observed in BAF-depleted cells, supporting that destabilization of CENP-C is their principal cause. The mechanism by which BAF stabilizes CENP-C at centromeres remains unknown. It is possible that cenBAF modifies centromeric chromatin in a way that stabilizes CENP-C, since BAF has been shown to affect histone modifications and higher-order chromatin organization^2,3,15,17,26^. It is also possible that the stabilization is through the action of PP4, since cenBAF is required for centromeric localization of Flfl. On the other hand, CENP-C destabilization at centromeres likely involves tension exerted by spindle microtubules since, when centromeric localization of cenBAF and PP4 are impaired in CENP-CΔFIM-expressing cells, CENP-C delocalizes to centrosomes and across the spindle in metaphase chromosomes^24^ (see also Fig. 6c, metaphase in bottom panel). Our results also show that cenBAF is reciprocally stabilized by CENP-C via the recruitment of Flfl. Altogether these observations suggest that the network of interactions between CENP-C, PP4, and cenBAF forms a positive feedback loop that reinforces assembly of centromeric chromatin and, hence, ensures faithful chromosome segregation. BAF depletion also affected centromeric CENP-A^CID^ levels. This effect is likely a consequence of CENP-C destabilization, since CENP-A^CID^ was reduced to a much lesser extent than CENP-C and it is known that CENP-A^CID^ and CENP-C are interdependent for their centromeric localization^35–37^.

**Fig. 9:**
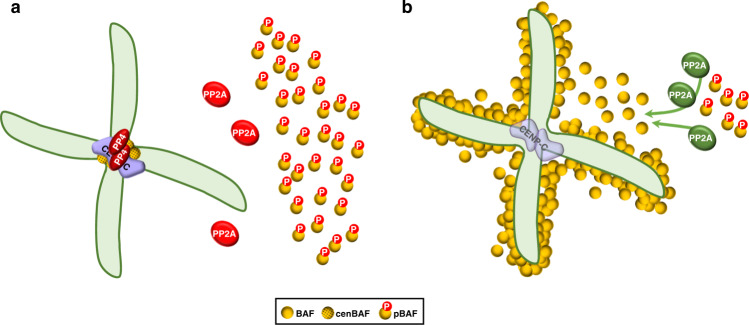
cenBAF, PP4, and CENP-C form a centromeric network that prevents PP2A-mediated dephosphorylation and perichromosomal accumulation of BAF in mitosis. **a** Normally, centromeric localization of cenBAF and PP4, which depends on CENP-C, maintains PP2A inactive (in red) during mitosis and, consequently, the bulk of BAF stays phosphorylated and free. **b** Disrupting centromeric localization of cenBAF and PP4 causes ectopic PP2A activation (in green) in mitosis, which results in dephosphorylation of pBAF and the accumulation of perichromosomal BAF. Centromeric CENP-C is also destabilized. See text for details.

The small fraction of cenBAF regulates the behavior of the large pool of free pBAF in mitosis. Disrupting cenBAF localization induces PP2A-mediated dephosphorylation of free pBAF in mitosis and the accumulation of BAF in a perichromosomal layer that surrounds chromosomes (Fig. 9). Normally, PP2A is inactivated at the entry into mitosis (reviewed in ref. ^38^). Thus, our results suggest that in the absence of cenBAF, PP2A remains active in mitosis. How might cenBAF regulate PP2A activity in mitosis remains to be determined. In this regard, PP4 could play a central role, since our results suggest that it regulates PP2A-mediated pBAF dephosphorylation. Whether the centromere-bound fraction of PP2A^39^ participates in this regulatory mechanism remains to be determined too.

PP2A selectively dephosphorylates 1pBAF, but not 2pBAF, suggesting that various phosphatases specifically target pBAF. In this regard, in *Drosophila*, a second unidentified phosphatase has been proposed to dephosphorylate pBAF at the exit from mitosis^6^. PP4 might be involved in 2pBAF dephosphorylation since, though weakly, Flfl depletion increased 2pBAF levels. Further work is required to clarify the actual phospho-sites in 1pBAF and 2pBAF and the potential site-specific activity of the various phosphatases involved in BAF dephosphorylation.

cenBAF disruption compromises progression through mitosis, delaying AO and increasing total mitosis duration. Several factors could contribute to these effects. On one hand, defects in centromere and kinetochore assembly are known to delay or arrest mitosis progression, particularly before AO. Furthermore, altering PP2A activity could impact mitosis in many different ways. In this regard, impaired BAF phosphorylation was shown to affect mitosis progression, since VRK1 depletion in mammalian cells, which also prevents BAF phosphorylation and its release from chromatin during mitosis, delays AO and increases mitosis duration too^22^. Delayed AO could reflect a defect in NEBD since BAF phosphorylation is important to weaken anchoring of chromatin to the NE^1,21,22^. On the other hand, exiting mitosis, pBAF dephosphorylation is crucial for NER^3,5,6,23^. Thus, it is also possible that, due to the ectopic activation of PP2A in mitosis, cenBAF disruption induces premature pBAF dephosphorylation and NER. The increased proportion of mitotic cells showing assembled NE, and the persistence of Nup-107::mRFP signal through mitosis, support a contribution of cenBAF to NE disassembly/reassembly. Along the same lines, cenBAF disruption induces strong NE morphological defects. Altered nuclear morphology is widely associated with generic mitotic problems. However, the defects observed upon impairing cenBAF localization are rescued by constitutive targeting of BAF to centromeres, indicating that they are linked to cenBAF disruption. Moreover, BAF mutations that affect its ability to polymerize and cross-bridge distant DNA sites^7^, or when BAF phosphorylation is impeded by VRK1 depletion^1,22^, induce similar nuclear morphology defects. Altogether these results suggest that cenBAF, although localized at centromeres, participates in the global regulation of the structural rearrangements that the NE undergoes during mitosis. Further work is required to reach a better understanding of this contribution.

In summary, our results suggest that, together with PP4 and CENP-C, cenBAF forms a functional centromeric network that is required for accurate chromosome segregation and controls mitosis progression by regulating PP2A-mediated BAF dephosphorylation. It is tempting to speculate that this network helps to coordinate chromosome segregation with the crucial NE rearrangements that mark mitosis progression. Interestingly, other NE components have also been reported to associate with the centromere/kinetochore during mitosis and contribute to spindle assembly^40–42^, revealing the strong functional links that exist between the NE and the centromere/kinetochore.

## Methods

### DNAs, protein constructs, cell lines, and antibodies

cDNA encoding BAF was obtained from *Drosophila* Genomics Resource Center (clone GH06291). Plasmids expressing BAF::YFP and CENP-A^CID^::YFP under the control of their own promoters were obtained by cloning the appropriate constructs into pEYFP (Clontech). BAF^R^ constructs resistant to RNAi knockdown were obtained by modifying codon usage following the *Drosophila* RNAi escape strategy construct (RESC). Plasmid expressing GBP::FLAG::BAF under the control of the copper-inducible metallothionein promoter was generated by standard PCR and Gateway cloning methods. Briefly, the GBP^30^ encoding sequence was PCR amplified using primers described in Supplementary Table 2 and cloned into the pMT-3xFlag-DEST vector upstream to and in frame with the 3xFlag-tag. This new product was used in LR reaction with the BAF-entry clone to generate pMT-GBP::FLAG::BAF. Plasmid expressing FLAG::BAF under the control of its own promoter was obtained by cloning BAF into pEYFP plasmid (Clontech) and replacing the YFP tag by the FLAG-tag. Plasmids FLAG::BAF^S5A^, FLAG::BAF^3A^, and FLAG::BAF^3E^ were obtained from plasmid FLAG::BAF mutating S5 to A and S2, T4 and S5 to A or E, respectively. Plasmid expressing CENP-A^CID^::TAP under the control of its own promoter was obtained by cloning the appropriate construct into plasmid pMK33-C::TAP (Clontech). Plasmid pMT-NHK1 expressing VRK1/NHK1::FLAG::HA construct was obtained from BDGP (clone FMO02828). Plasmid pMT-Nup-107::mRFP was a gift from Dr. Helder Maiato and is described in ref. ^43^.

Cultured cells used in these experiments were Schneider’s *Drosophila* Line 2 [D. Mel. (2), SL2] (ATCC^®^ CRL-1963™). To generate cell lines stably expressing RNAi-resistant (^R^) GFP::CENP-C^R^ and GFP::CENP-CΔFIM^R^ constructs, we replaced the Acc65I-EcoRI (420 bps) fragment in GFP::CENP-C and GFP::CENP-CΔFIM constructs^24^ to a codon modified version of the fragment (GeneArt gene synthesis) that encodes the same amino acids but is insensitive to RNAi targeting endogenous CENP-C. Stable lines expressing CENP-A^CID^::TAP, YFP::CENP-A^CID^, BAF::YFP, GBP::FLAG::BAF/GFP::CENP-C^R^, GBP::FLAG::BAF/GFP::CENP-CΔFIM^R^, Nup-107::mRFP/GFP::CENP-C^R^, and Nup-107::mRFP/GFP::CENP-CΔFIM^R^ were obtained according to standard procedures.

Rabbit polyclonal αBAF, and rat and rabbit polyclonal αCENP-C antibodies were raised against bacterially expressed full length *Drosophila* BAF and a *Drosophila* CENP-C fragment (aa 505–1227), respectively. Specificity of the antibodies was determined by WB and/or immunostaining (IF) (Supplementary Figs. 1 and Fig. 8d). Rabbit polyclonal αCID, and rat polyclonal αHP1a and αHP1c antibodies are described in refs. ^44,45^. Rat polyclonal αFlfl antibodies are described in ref. ^24^. Rabbit polyclonal αBAFpS5 antibodies raised against a phospho-peptide spanning the N-terminal region of human BAF and are described in ref. ^23^. Specificity of αBAFpS5 antibodies for *Drosophila* pBAF was determined by phos-tag gel electrophoresis analysis, in which αBAFpS5 antibodies recognized pBAF, but not non-phosphorylated BAF, and a S5A mutation abolished this reactivity (Supplementary Fig. 5e). The rest of antibodies used were commercially available: rabbit polyclonal αTAP (Open Biosystems, CAB1001), mouse monoclonal αMTS (BD-Transduction Laboratories, 610555), mouse monoclonal αFLAG (Sigma F3165), rabbit purified αFLAG (Sigma F7425), mouse monoclonal αTubulin (Millipore, MAB3408), mouse monoclonal αLaminB (DSHB ADL67.10), mouse monoclonal αGFP (Roche 1181446001), rabbit polyclonal αGFP (Invitrogen A11122), rabbit polyclonal αH3 (Cell Signaling 9715), and rabbit polyclonal αPS10 (Millipore 06-570).

### Fly stocks and genetic procedures

nub*-GAL4* flies were obtained from Bloomington Stock Center*. baf*^*RNAi*^ corresponds to 102,013 stock from the Vienna *Drosophila* RNAi Center. Transgenic flies carrying the various UAS-dBAF constructs described in the text were obtained by site-directed integration of the corresponding pUASTattb plasmids into chromosome 3 using 3R-86Fb embryos.

For experiments with knockdown *baf*^*RNAi*^ flies, crosses were left at 25 °C until third-instar larvae stage. For overexpression experiments, homozygous transgenic lines carrying the corresponding UAS-constructs were crossed to homozygous *nub-*GAL4 flies. To analyze the effects on wing development, flies were kept in 75% ethanol, 25% glycerol solution for at least 24 h at room temperature and washed in PBS. Then, wings were dissected and immediately mounted in Fauré’s medium under gentle pressure. Images were collected using a ×4 objective lens on a Nikon E-600 microscope equipped with an Olympus DP72 camera and CellF software.

### Identification of CENP-A^CID^ chromatin-associated proteins

For the identification of proteins associated with CENP-A^CID^-enriched chromatin, a stable S2 cell line expressing CENP-A^CID^::TAP under the control of the CENP-A^CID^ promoter was used. The pattern of CENP-A^CID^::TAP localization was determined by immunostaining with αTAP antibodies (Supplementary Fig. 12a, b). TAP-affinity purification of proteins associated with CENP-A^CID^::TAP containing chromatin was performed as described in ref. ^46^. Briefly, nuclei were purified and digested with micrococcal nuclease (MNase) (Sigma). After digestion was stopped, the soluble chromatin fraction (SN1), which accounted for ~66% of total chromatin, was prepared by centrifugation at 10,000×*g* for 15 min at 4 °C. The remaining insoluble material was extracted at increasing EDTA concentration from 2 to 200 mM. Nucleosomal composition of each fraction was analyzed by agarose gel electrophoresis (Supplementary Fig. 12c). CENP-A^CID^::TAP and CENP-C content were determined by WB (Supplementary Fig. 12d, e). Then, the SN1, 2 and 20 mM EDTA fractions were subjected to conventional TAP-affinity purification using IgG-Dynabeads (Invitrogen). Bound proteins were eluted and analyzed by standard LC/MS at the Proteomics Unit of the “Institut de Recerca de la Vall dʼHebron” (Barcelona). Supplementary Table 1 summarizes the proteins identified in these studies, which included BAF (mascot score: 97; sequence coverage: 16.7%).

### RNAi knockdown experiments

For RNAi-mediated BAF knockdown experiments, dsRNA encompassing the entire BAF-coding region was prepared using the MEGAscript T7 kit (Ambion). Then, cells were incubated with 20 µg of dsRNA at a concentration of 5 × 10^5^ cells/ml and, after 3 days, cells were diluted 1:2 and treated with a second dose of 20 µg of dsRNA for 4 days. CENP-C, VRK1/NHK1, and Flfl knockdown experiments were performed as for BAF using 40–50 µg (CENP-C) and 30 µg (VRK1/NHK1 and Flfl) of the corresponding dsRNA. MTS knockdown was performed with a single dose of 30 µg of dsRNA for 3 days. When MTS depletion was combined with Flfl knockdown, dsRNA against MTS was added with the second dose of dsRNA against Flfl. The extent of BAF, CENP-C, MTS, and Flfl depletion were determined by WB and/or IF. The extent of VRK1/NHK1 depletion was assessed from the effects on BAF phosphorylation (Supplementary Fig. 5a). Primers used in these experiments are indicated in Supplementary Table 2.

### Immunostaining experiments

Immunostaining experiments were performed as described elsewhere^44^. Briefly, cells were treated for 6 h with 25 μM colchicine (Sigma), immobilized onto a slide by centrifugation for 10 min at 500 rpm with low acceleration in a TermoShandon Cytospin using a single-chamber Cytofunnel and, then, fixed in 4% paraformaldehyde for 10 min, washed with PBS and blocked in 3% BSA, 0.5% TritonX-100 in PBS. Samples were then immunostained with αBAF (1:300), αBAFpS5 (1:200), rat αCENP-C (1:500), rabbit αCENP-C (1:300), αCENP-A^CID^ (1:500), αHP1a (1:200), αHP1c (1:500), αFlfl (1:1000), αFLAG (1:2000), αTubulin (1:10,000), αTAP (1:300), and αLaminB (1:1500) antibodies. For visualization, slides were mounted in Mowiol (Calbiochem-Novabiochem) containing 0.2 ng/ml DAPI (Sigma) and analyzed in a Leica TCS/SPE confocal microscope equipped with LAS/AF software. Images were acquired and processed identically using ImageJ (http://imagej.nih.gov/ij/) and Adobe Photoshop software. Mean grey intensities were calculated using ImageJ macros^47^ on thresholded images at DAPI-masked regions of interest running analyzed particles to plugin on the FeatureJ Laplacian (http://imagescience.org/meijering/software/featurej/).

For super-resolution microscopy, samples were mounted in Vectashield antifade mounting medium containing DAPI (Vector Laboratories). Images were taken with a Zeiss 880 confocal microscope equipped with Airyscan for image acquisition. A ×100 magnification 1.46 NA oil-immersion lens with a digital zoom of ×3 was used. The Z-step between the stacks was set at 167.9 nm. Airyscan raw data were preprocessed with the automatic setting of Zen Black. For the generation of the intensity profile plots a segmented line was manually drawn on a single z-stack and analyzed on ImageJ. The intensity profile was calculated for each staining separately.

### Live cell imaging

Stable cell lines expressing Nup-107::mRFP/GFP::CENP-C^R^ and Nup-107::mRFP/GFP::CENP-CΔFIM^R^ were treated with dsRNA against CENP-C as described above. Cells were plated 24 h before live imaging in poly-d-lysine dishes (MatTek Corporation P35GC-1.5-14C). Expression of the tagged constructs was induced by adding 500 μM CuSO_4_ to the media 20 h before live imaging. Live imaging was performed on an Ultraview ERS6 spinning disc system mounted on a Zeiss Axiovert 200M inverted microscope and equipped with a Hamamatsu C9100-50 electron-multiplied camera and a Plan-Neofluar ×40/1.3NA oil objective. z-stacks covering the entire volume of the mitotic cell were collected every 2 min at a step size of 8 nm, using the acquisition software Volocity 6.1. Images and movies were processed and analyzed in ImageJ. Merged images represent maximum intensity projections of all z-stacks.

### Chromatin fibers

For fiber analyses, extended chromatin fibers were prepared essentially as described in ref. ^48^. Briefly, fibers were extended in 450 mM NaCl and immunostained with αBAF (1:200) and αCENP-C (1:300) antibodies. Images were acquired with a ×63 objective on a SP5 Leica confocal microscope equipped with LAS/AF software and analyzed with Image J and Adobe Photoshop software.

### NE and chromosome segregation defects

For these experiments, cells were plated at 0.5 × 10^6^ cells/ml in well plates containing cover slips coated with Concanavalin A (0.5 mg/ml; Sigma) and, after 18 h, cells were processed for immunostaining. To monitor nuclear morphology and chromosome segregation defects, cells were stained with αLamin (1:1000) and αTubulin (1:5000) antibodies, respectively. To analyze NE assembly in mitosis, cells were co-stained with αLamin (1:1000) and αPS10 (1:3000) antibodies.

### Co-IP experiments

For Co-IP experiments, cell extracts were obtained in 50 mM Tris–HCl pH 8, 150 mM NaCl, 5 mM EDTA, 0.5% NP-40, 0.1 mM PMSF, Protease Inhibitor Cocktail and, after homogenization with Dounce (B pestle), supplemented to 300 mM NaCl and centrifuged at 14,000 rpm for 15 min at 4 °C. The supernatant was incubated overnight at 4 °C with the indicated antibodies or preimmune serum as control (mock). Then, Protein A Sepharose beads (GE Healthcare) were added and incubated at 4 °C for 2 h. After incubation, beads were pelleted by centrifugation, washed and eluted in PLB 1X, 10% β-mercaptoethanol, and analyzed by WB. For treatment with DNAse I, extracts were supplemented with 20 mM MgCl_2_ and 3 mM CaCl_2,_ and incubated at 37 °C for 30 min with Turbo^TM^ DNAse I (Ambion) at a final concentration of 0.4 units/μl. Digestion was stopped by adding 10 mM EDTA. Before DNAse I treatment, 30 μg of pUC19 were added to monitor the efficiency of digestion.

### Analysis of BAF phosphorylation

To analyze BAF phosphorylation, total cell extracts were obtained in PLB and analyzed by Phos-tag gel electrophoresis according to manufacturers’ instructions (Wako Chemicals Inc.). Briefly, 50 μM of acrylamide-pendant Phos-tag^TM^ (AAL-107) and 100 μM of MnCl_2_ were added to 10% polyacrylamide resolving gel solution before polymerization. After electrophoresis, gels were incubated 15 min in transfer buffer with 1 mM EDTA and 15 min in transfer buffer without EDTA, and analyzed by WB. For alkaline phosphatase (AP) treatment, cell extracts were obtained in 150 mM NaCl, 50 mM Tris–HCl pH 8, 10% glycerol, 0.1% SDS, 1% NP40, 1 mM PMSF, protease inhibitor cocktail, 50 mM NaF, 2 mM Na_3_VO_4_, and 10 mM glycerol phosphate. AP treatment was performed with calf intestine AP (Roche) for 1 h at 37 °C in 150 mM NaCl, 50 mM Tris–HCl pH 8, 10 mM MgCl_2_, and 1 mM DTT.

### Western blot analysis

Western blot analysis was performed according to standard procedures using the following antibody dilutions: αBAF (1:2500), αBAFpS5 (1:1000), αCENP-C (1:3000), αFLFL (1:10,000), αMTS (1:5000), αCENP-A^CID^ (1:2000), αHP1a (1:10,000), αH3 (1:2500), αTubulin (1:5000), αGFP (1:2000), αFLAG (1:2500), αTAP (1:2500). For all WBs presented in main and supplementary figures, uncropped images are presented in Supplementary Fig. 13.

### Statistics and reproducibility

Statistical significance of the difference in the proportion of mitoses showing segregation defects, perichromosomal BAF and centromeric Flfl, and in the proportion of cells with aberrant NE morphology was assessed via two-tailed Fisher’s exact test. Statistical significance of the difference in the proportion of NE-assembled mitoses between GFP::CENP-C^R^ and GFP::CENP-CΔFIM^R^-expressing cells was assessed via comparative Chi-square test using the cp.chisq.test function from the DiffXTables package version 0.1.0 using R 3.5.1^49^. One-tailed binomial test was used to assess the probability to find BAF and BAF-YFP co-localizing with CENP-C on the same chromatin fiber. Statistical significance of the centromeric localization of BAF phosphomutants was assessed by two-tailed binomial test. Statistical significance of the difference in centromeric intensity of BAF, CENP-C, and CENP-A^CID^ immunostaining was determined by Kruskal–Wallis test. Statistical difference in the extent of BAF/CENP-C co-IP and the changes in BAF phosphorylation was determined by two-tailed *t*-test comparison of the means. For each experiment, the number of independent biological replicates and sample sizes are indicated in the corresponding figure legend.

### Reporting summary

Further information on research design is available in the Nature Research Reporting Summary linked to this article.

## Supplementary information

Supplementary Information

Description of Additional Supplementary Files

Supplementary Data 1

Supplementary Movie 1

Supplementary Movie 2

Supplementary Movie 3

Supplementary Movie 4

Supplementary Movie 5

Supplementary Movie 6

Reporting Summary

Peer Review File

Supplementary Data 2

## Data Availability

All data and unique materials in this paper are available from the corresponding authors upon reasonable request. All data underlying the graphs described in the main and supplementary figures are presented in Supplementary Data 1.
