# Supplementary material for: A fraction of barrier-to-autointegration factor (BAF) associates with centromeres and controls mitosis progression
Source: Commun Biol. 2020 Aug 19;3:454. doi: 10.1038/s42003-020-01182-y (PMC7438335; doi:10.1038/s42003-020-01182-y)
Supplement: Supplementary file 1 — Supplementary Information [file 42003_2020_1182_MOESM1_ESM.pdf]

SUPPLEMENTARY FIGURES

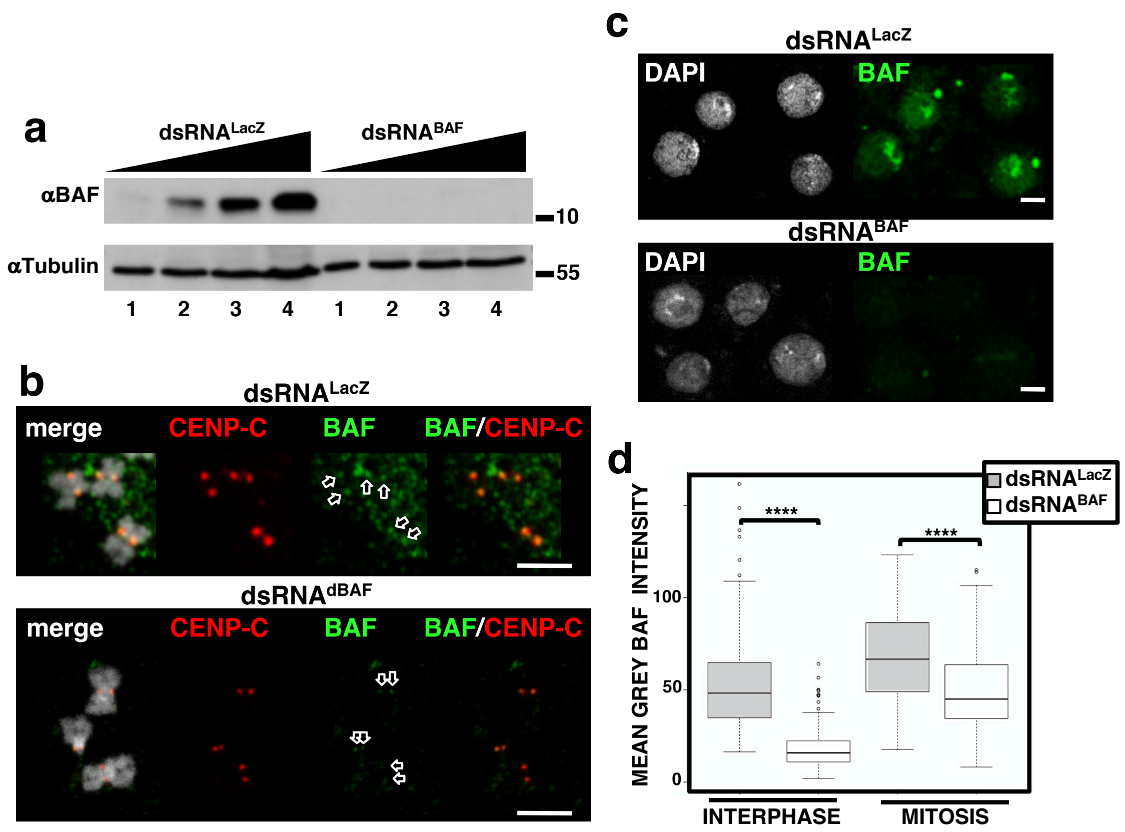

Supplementary Figure 1. Efficiency of RNAi-mediated knockdown of BAF. (**a**) WB analysis with αBAF antibodies of increasing amounts of total cell extracts (lanes 1-4) prepared from S2 cells treated with dsRNA against BAF (dsRNA^{BAF}) (right) or against LacZ (dsRNA^{LacZ}) (left). αTubulin antibodies were used for loading control. The position of MW markers (in kDa) is indicated. (**b**) Immunostainings with αBAF antibodies (green) and αCENP-C antibodies (red) are presented for dsRNA^{BAF} (bottom) and control dsRNA^{LacZ} (top) metaphase chromosomes. Arrows indicate αBAF signals overlapping with αCENP-C signals at centromeres. DNA was stained with DAPI. Scale bars correspond to 5μm. (**c**) Immunostaining with αBAF antibodies (green) of dsRNA^{BAF} (bottom) and control dsRNA^{LacZ} (top) interphase cells. DNA was stained with DAPI. Scale bars correspond to 5μm. (**d**) Quantitative analysis of the results shown in **b** and **c**. The mean grey value of αBAF fluorescence is shown for dsRNA^{BAF} and control dsRNA^{LacZ} cells in interphase and per centromere in metaphase chromosomes. Values correspond to a representative experiment out of 5 independent

experiments showing equivalent results (N>228 for interphase cells and N>116 for mitosis; Kruskal-Wallis test, p-value ****< 0.0001).

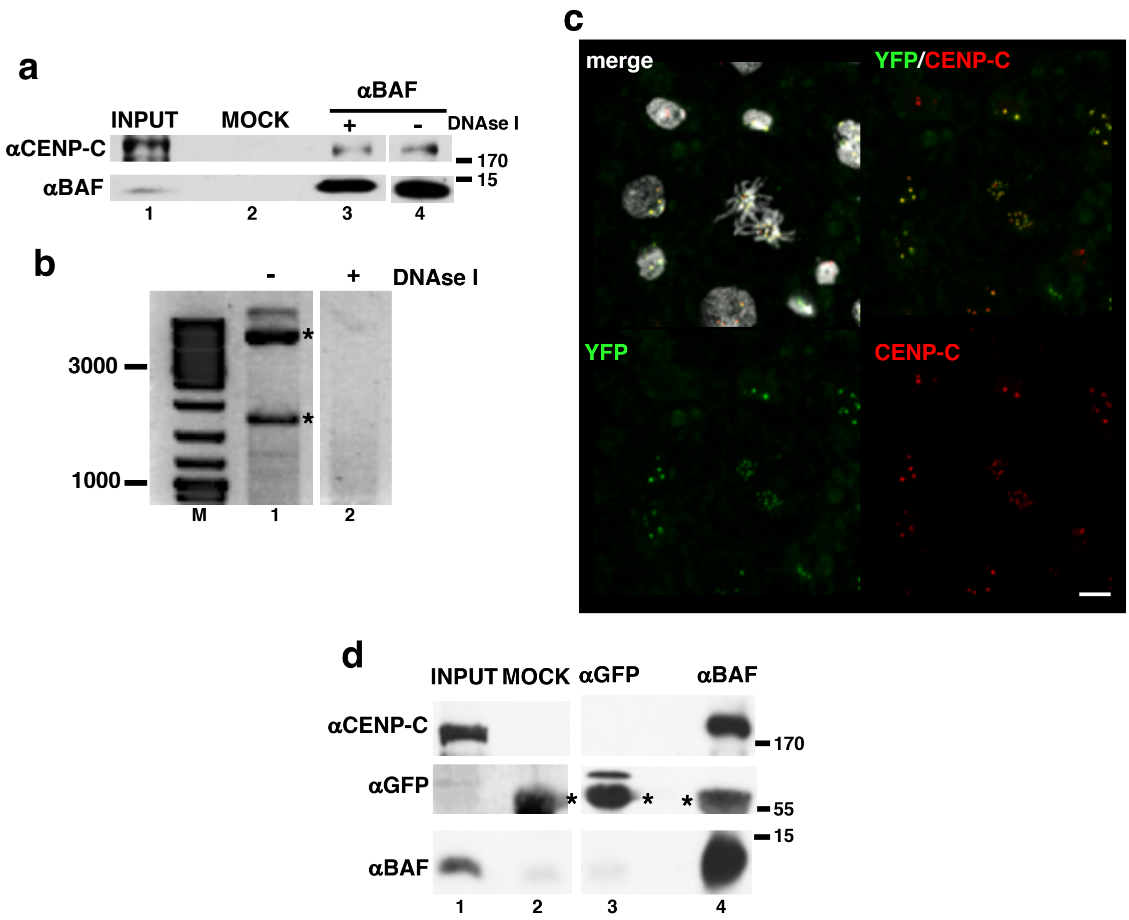

Supplementary Figure 2. CENP-C/BAF co-immunoprecipitation is independent of chromatin binding. (**a**) IPs with αBAF antibodies after treatment of the extract with DNAse I (lane 3) or not (lane 4), and with control preimmune serum (MOCK) (lane 2) are presented. IP-materials are analyzed by WB using αCENP-C and αBAF antibodies. Lane 1 corresponds to 4% of the input material. The position of MW markers (in kDa) is indicated. (**b**) DNA agarose gel electrophoretic analysis to determine the efficiency of DNAse I treatment. Prior to immunoprecipitation with αBAF antibodies, pUC19 plasmid DNA was added and, then, the extract was treated with DNAse I (lane 2) or not (lane 1). Asterisks indicate the bands corresponding to pUC19. Lane M corresponds to molecular weight markers. The size in bp of selected markers is indicated. (**c**) The pattern of localization of CENP-A^{CID}::YFP is determined by direct fluorescence (green) in

stable S2 cells expressing CENP-A^{CID}::YFP. Immunostaining with αCENP-C antibodies (red) is also presented. DNA was stained with DAPI. Scale bar corresponds to 5μm. (**d**) co-IPs with αGFP (lane 3), αBAF (lane 4) and with control preimmune serum (MOCK) (lane 2) using extracts prepared from a stable S2 line expressing CENP-A^{CID}::YFP are presented. Lane 1 corresponds to 3% of the input material. IP-materials are analyzed by WB with αCENP-C, αGFP and αBAF antibodies. Asterisks (*) indicate IgGs. The position of MW markers (in kDa) is indicated.

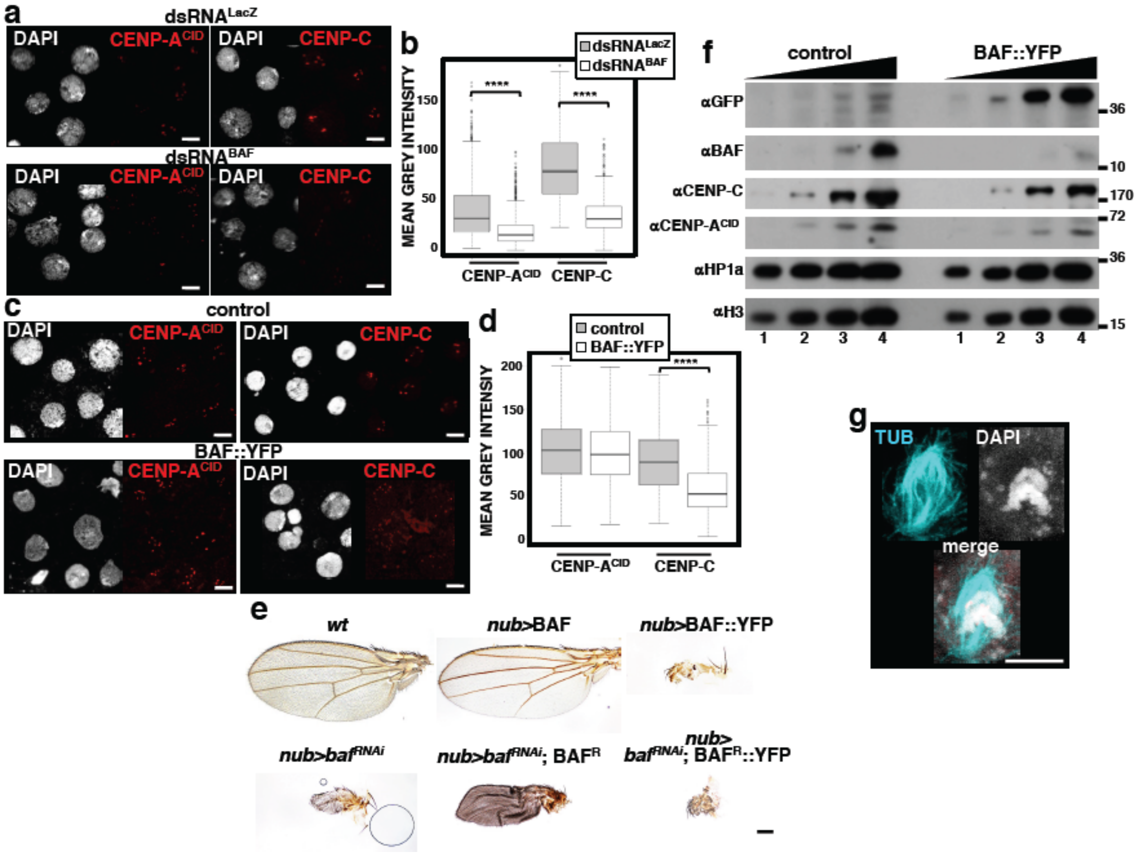

Supplementary Figure 3. The effect of BAF on centromere assembly and chromosome segregation. (**a**) Immunostainings with αCENP-A^{CID} (left) and αCENP-C (right) antibodies (red) are presented in dsRNA^{BAF} (bottom) and control dsRNA^{LacZ} (top) cells. DNA was stained with DAPI. Scale bars correspond to 5μm. (**b**) Quantitative analysis of the results shown in **a**. The mean grey values per centromere of αCENP-A^{CID} and αCENP-C fluorescence are shown for dsRNA^{BAF} and control dsRNA^{LacZ} cells. Values correspond to a representative experiment out of 5 independent experiments showing equivalent results (N>639;

Kruskal-Wallis test, p-value ****< 0.0001). (**c**) Immunostainings with αCENP-A^{CID} (left) and αCENP-C (right) antibodies (red) are presented in a stable S2 line expressing BAF::YFP (bottom), and in control non-expressing cells (top). DNA was stained with DAPI. Scale bars correspond to 5μm. (**d**) Quantitative analysis of the results shown in **c.** The mean grey value per centromere of αCENP-C and αCENP-A^{CID} fluorescence is presented for cells expressing BAF::YFP and control non-expressing cells. Values correspond to a representative experiment out of 3 independent experiments showing equivalent results (N> 485; Kruskal-Wallis test, p-value ****< 0.0001). (**e**) Wings from control flies (*wt*), knockdown *baf*^{RNAi} flies (*nub>baf*^{RNAi}), flies overexpressing BAF (*nub>*BAF) or BAF::YFP (*nub>*BAF::YFP) in a *wt* background, and flies overexpressing RNAi-resistant BAF^{R} (*nub>baf*^{RNAi}*;* BAF^{R}) or BAF^{R}::YFP (*nub>baf*^{RNAi}*;* BAF^{R}::YFP) in a knockdown *baf*^{RNAi} background. Scale bar corresponds to 200μm. (**f**) The levels of BAF::YFP and endogenous BAF, CENP-C, CENP-A^{CID} and HP1a are determined by WB in extracts prepared from cells expressing BAF::YFP (right) and control non-expressing cells (left) using αGFP, αBAF, αCENP-C, αCID and αHP1a antibodies. αH3 antibodies (bottom) were used for loading control. Increasing amounts of extract (lanes 1-4) are analyzed. The position of MW markers (in kDa) is indicated. (**g**) Metaphase figure from S2 cells expressing BAF::YFP. The spindle was stained with αTubulin antibodies (blue). DNA was stained with DAPI. Scale bar corresponds to 5μm.

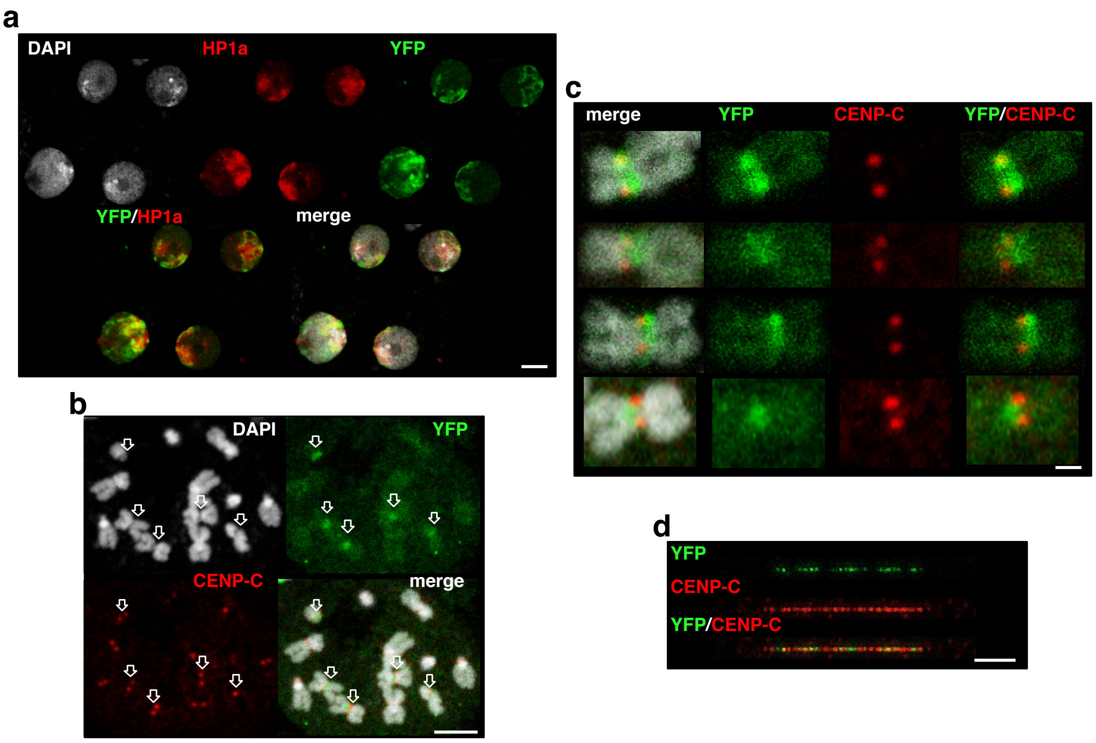

Supplementary Figure 4. Pattern of localization of BAF::YFP. (**a**) The pattern of localization of BAF::YFP is determined by direct fluorescence (green) in interphase cells from a stable S2 line expressing BAF::YFP. Immunostaining with αHP1a antibodies (red) is also presented. DNA was stained with DAPI. Scale bar corresponds to 40μm. (**b**) The pattern of localization of BAF::YFP is determined by direct fluorescence (green) in metaphase chromosomes of a stable S2 line expressing BAF::YFP. Immunostaining with αCENP-C antibodies (red) is also presented. DNA was stained with DAPI. Arrows indicate YFP signals detected at centromeric regions. Scale bar corresponds to 5μm. (**c**) Enlarged images of metaphase chromosomes from cells expressing BAF::YFP. Scale bar corresponds to 5μm. (**d**) Chromatin fibers analyses from stable S2 cells expressing BAF::YFP. BAF::YFP signals were determined by direct fluorescence (in green), and CENP-C by immunostaining with αCENP-C antibodies (red). Scale bar corresponds to 5μm.

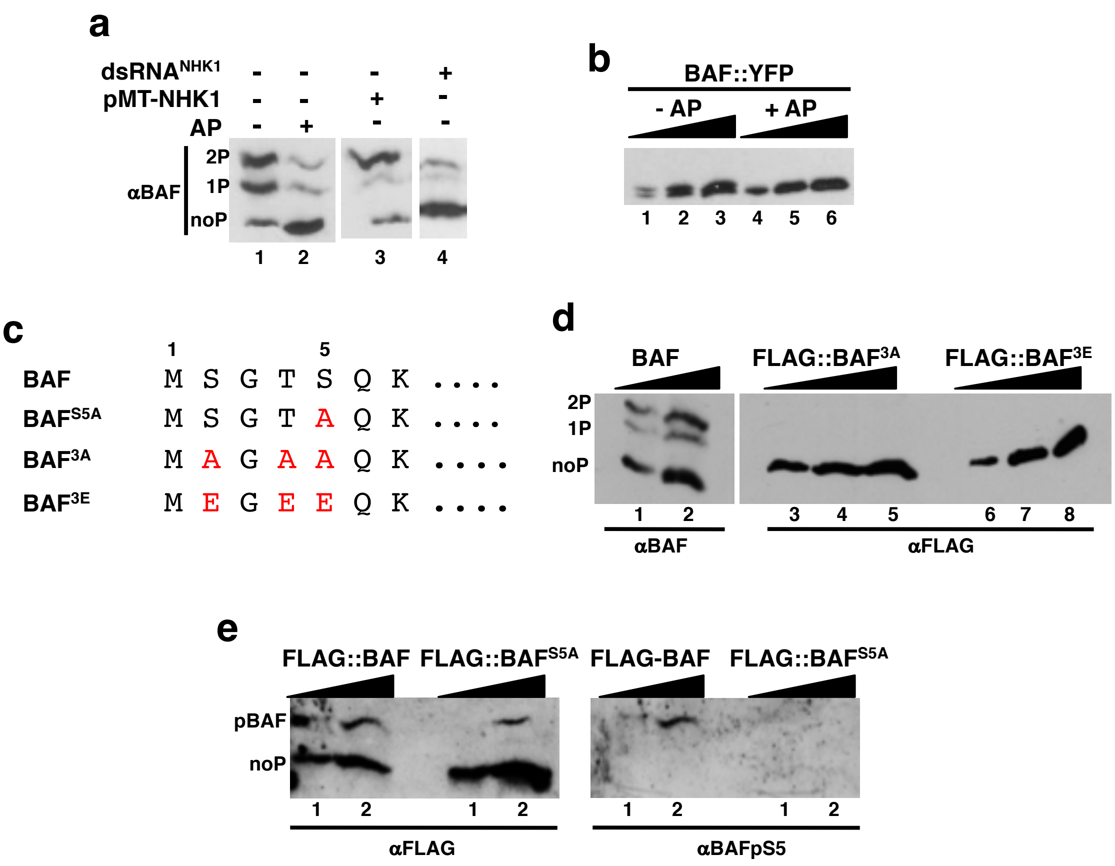

Supplementary Figure 5. **Analysis of BAF phosphorylation**. (**a**) The pattern of BAF phosphorylation is analyzed by phos-tag gel electrophoresis. Extracts were prepared from cells treated with dsRNA against VRK1/NHK1 (dsRNA^{NHK1}) (lane 4), overexpressing VRK1/NHK1 (pMT-NHK1) (lane 3) and control dsRNA^{LacZ} cells treated with alkaline phosphatase (AP) (lane 2) or not (lane 1). Extracts are analyzed by WB using αBAF antibodies. The positions corresponding to non-phosphorylated (noP), and mono- (1P) and di-phosphorylated (2P) BAF species are indicated. (**b**) The pattern of phosphorylation of BAF::YFP is analyzed by phos-tag gel electrophoresis. Extracts were prepared from cells expressing BAF::YFP treated with alkaline phosphatase (lanes 4-6) or not (lanes 1-3). Increasing amounts of extracts are analyzed by WB using αBAF antibodies. (**c**) Sequence of the BAF N-terminus (aa1-7). In red, VRK1/NHK1-phosphorylable residues that are replaced to A in the FLAG::BAF^{S5A} and the phosphonull FLAG::BAF^{3A} forms, and to E in the phosphomimetic FLAG::BAF^{3E} form. (**d**) The phosphorylation of endogenous BAF (left), FLAG::BAF^{3A} (center) and FLAG::BAF^{3E} (right) is analyzed by phos-tag gel electrophoresis. Increasing amounts of extracts (lanes 1 and 2) are

analyzed by WB using αBAF (left) and αFLAG (center and right) antibodies. The positions corresponding to non-phosphorylated (noP), and mono- (1pBAF) and di-phosphorylated (2pBAF) BAF species are indicated. (**e**) Phos-tag gel electrophoretic analysis of increasing amounts of extracts (lanes 1 and 2) prepared from cells transiently expressing FLAG::BAF and FLAG::BAF^{S5A} forms. Extracts are analyzed by WB using αFLAG (left) and αBAFpS5 antibodies (right). The positions corresponding to phosphorylated (pBAF) and non-phosphorylated (noP) species are indicated.

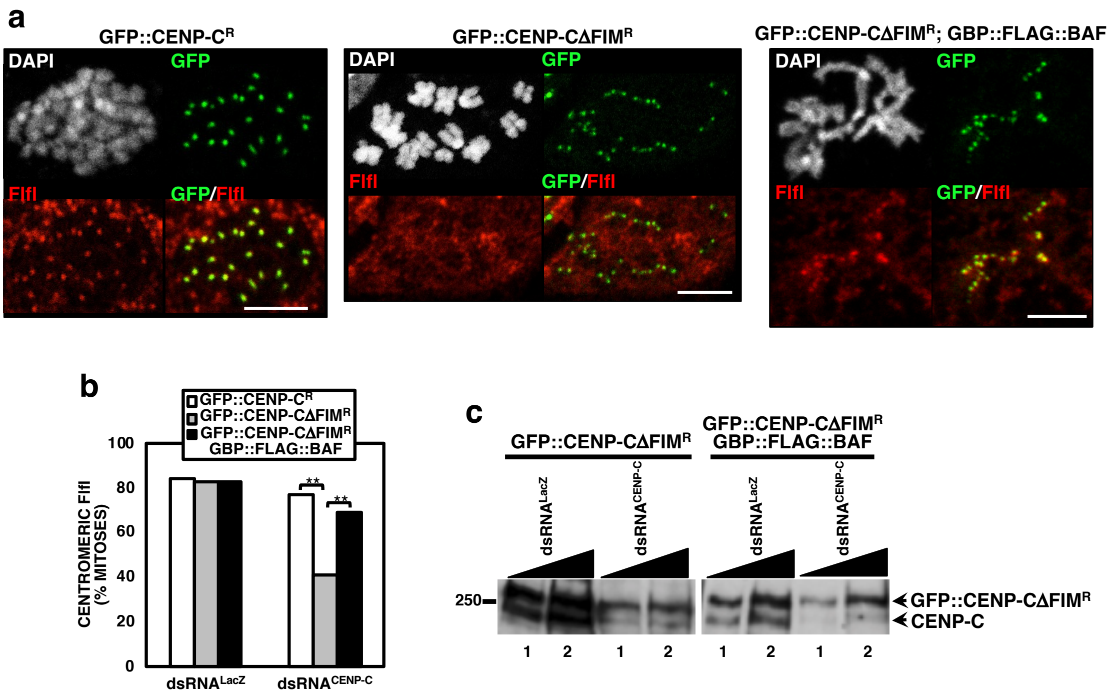

Supplementary Figure 6. Centromeric Flfl localization depends on CENP-C and is rescued in cells expressing GBP::FLAG::BAF. (**a**) Immunostainings with αFlfl antibodies (red) are presented for CENP-C depleted cells expressing the indicated constructs. GFP signals (green) are direct fluorescence. DNA was stained with DAPI. Scale bars correspond to 5μm. (**b**) Quantitative analysis of the results shown in **a**. The proportion of mitoses where Flfl is detected at the centromeres is presented for control dsRNA^{LacZ} and dsRNA$^{CENP\text{-}C}$ cells expressing the indicated constructs. Values are the sum of 3-5 independent experiments showing equivalent results (N> 45; two-tailed Fischer's test, p-value **<0.01). (**c**) WB analysis with αCENP-C antibodies of increasing amounts of

extracts (lanes 1 and 2) prepared from control dsRNA^{LacZ} and dsRNA$^{CENP\text{-}C}$ cells expressing the indicated constructs. The positions of endogenous CENP-C and the GFP::CENP-ΔFIM^{R} form are indicated. The position of MW markers (in kDa) is indicated.

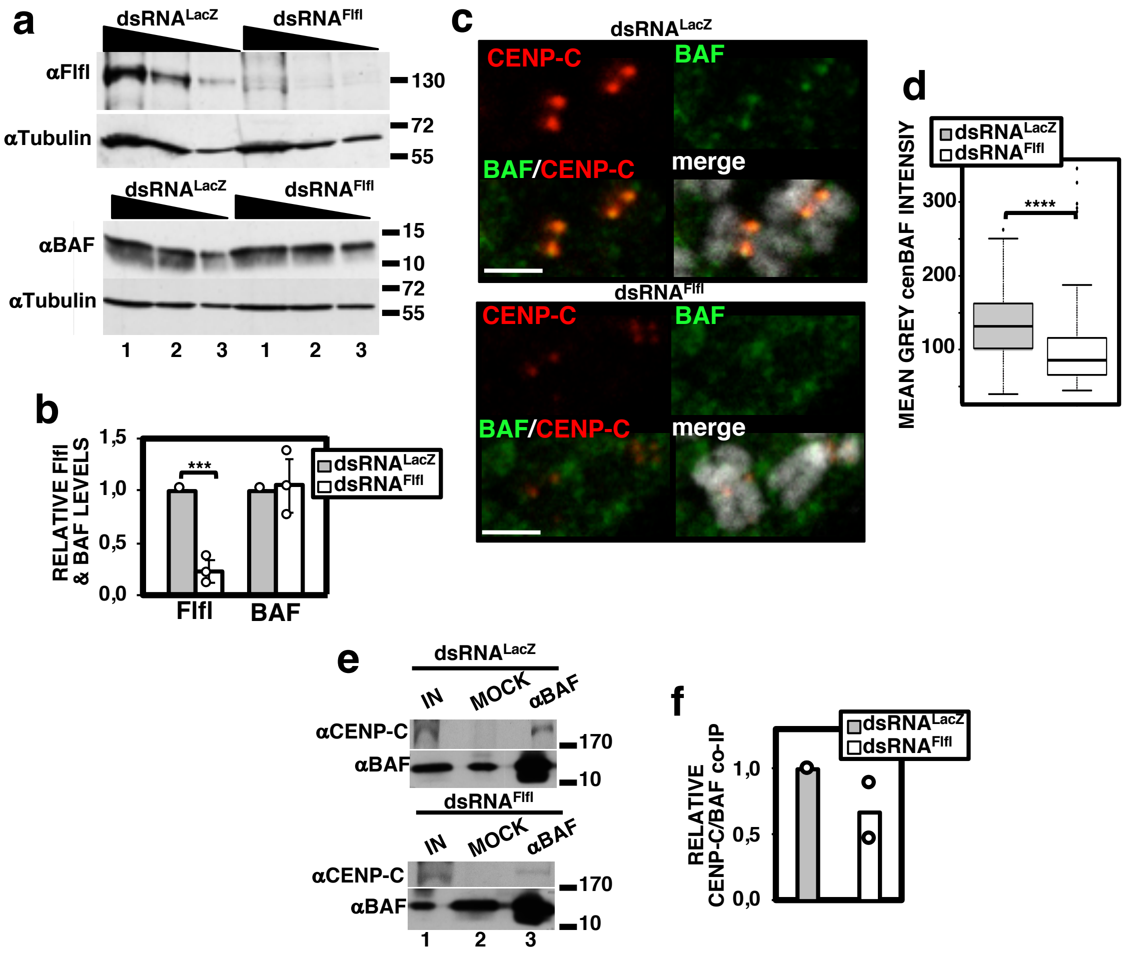

Supplementary Figure 7. Flfl depletion disrupts centromeric cenBAF localization. (**a**) The levels of Flfl (top) and BAF (bottom) are determined by WB in total cell extracts prepared from S2 cells treated with dsRNA against Flfl (dsRNA^{Flfl}) (right) or against LacZ (dsRNA^{LacZ}) (left). Increasing amounts of extract are analyzed (lanes 1-3). αTubulin antibodies are used for normalization. The position of MW markers (in kDa) is indicated. (**b**) Quantitative analysis of the results shown in **a**. The relative Flfl and BAF levels are presented for dsRNA^{Flfl} and control dsRNA^{LacZ} cells. Results are the average of 3 independent experiments (error bars are SD; two-tailed t-test, p-value ***< 0.001). (**c**) Immunostainings with αBAF (green) and αCENP-C antibodies (red) are

presented for mitotic chromosomes from dsRNA^{Flfl} (bottom) and control dsRNA^{LacZ} (top) cells. DNA was stained with DAPI. Scale bars correspond to 2.5μm. (**d**) Quantitative analysis of the results shown **c**. The mean grey values per centromere of αBAF fluorescence are shown for dsRNA^{Flfl} and control dsRNA^{LacZ} cells (n= 1; N= 67; Kruskal-Wallis test, p-value ****< 0.0001). (**e**) co-IP experiments performed with αBAF antibodies using extracts prepared from dsRNA^{Flfl} (bottom) and control dsRNA^{LacZ} (top) cells (lanes 3). Lanes 2 correspond to mock IPs performed with preimmune serum. Lanes 1 correspond to 3% of the input material. IP-materials are analyzed by WB using αCENP-C and αBAF antibodies. The position of MW markers (in kDa) is indicated. (**f**) Quantitative analysis of the results shown in **e**. The relative ratio of αCENP-C and αBAF signals is presented for dsRNA^{Flfl} and control dsRNA^{LacZ} cells. Results are the average of 2 independent experiments (two-tailed t-test, p-value > 0.05).

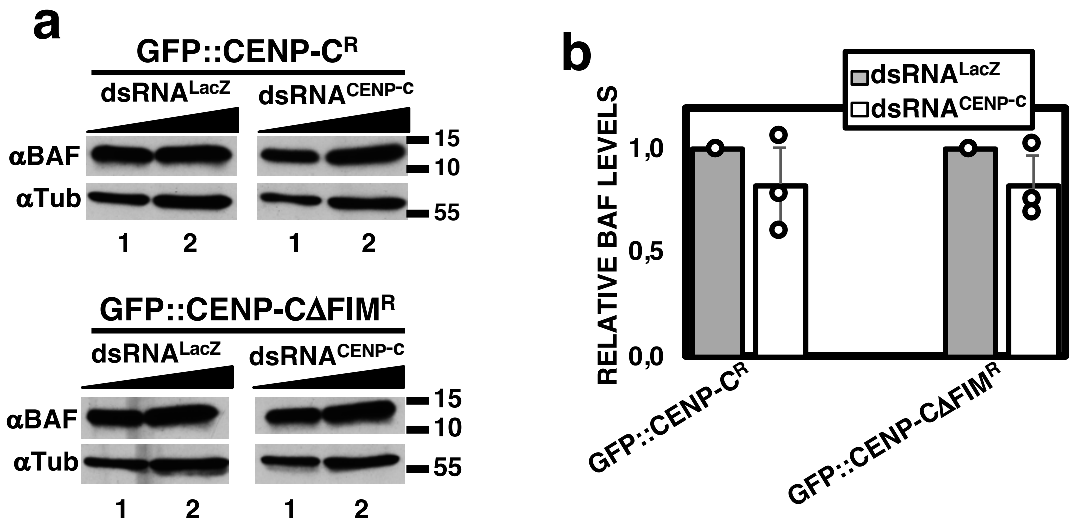

Supplementary Figure 8. Disrupting cenBAF localization does not increase total BAF levels. (**a**) The levels of BAF are determined by WB with αBAF antibodies in extracts prepared from cells expressing GFP::CENP-C^{R} (top) and GFP::CENP-CΔFIM^{R} (bottom) treated with dsRNA^{LacZ} (left) and dsRNA$^{CENP\text{-}C}$ (right). Increasing amounts of extract are analyzed (lanes 1 and 2). αTubulin antibodies are used for normalization. The position of MW markers (in kDa) is indicated. (**b**) Quantitative analysis of the results shown in **a**. The relative levels of BAF in cells expressing GFP::CENP-C^{R} and GFP::CENP-CΔFIM^{R} after

treatment with dsRNA^{LacZ} and dsRNA^{CENP-C} are presented. Results are the average of 3 independent experiments (error bars are SD; two-tailed t-test, p-value > 0.05).

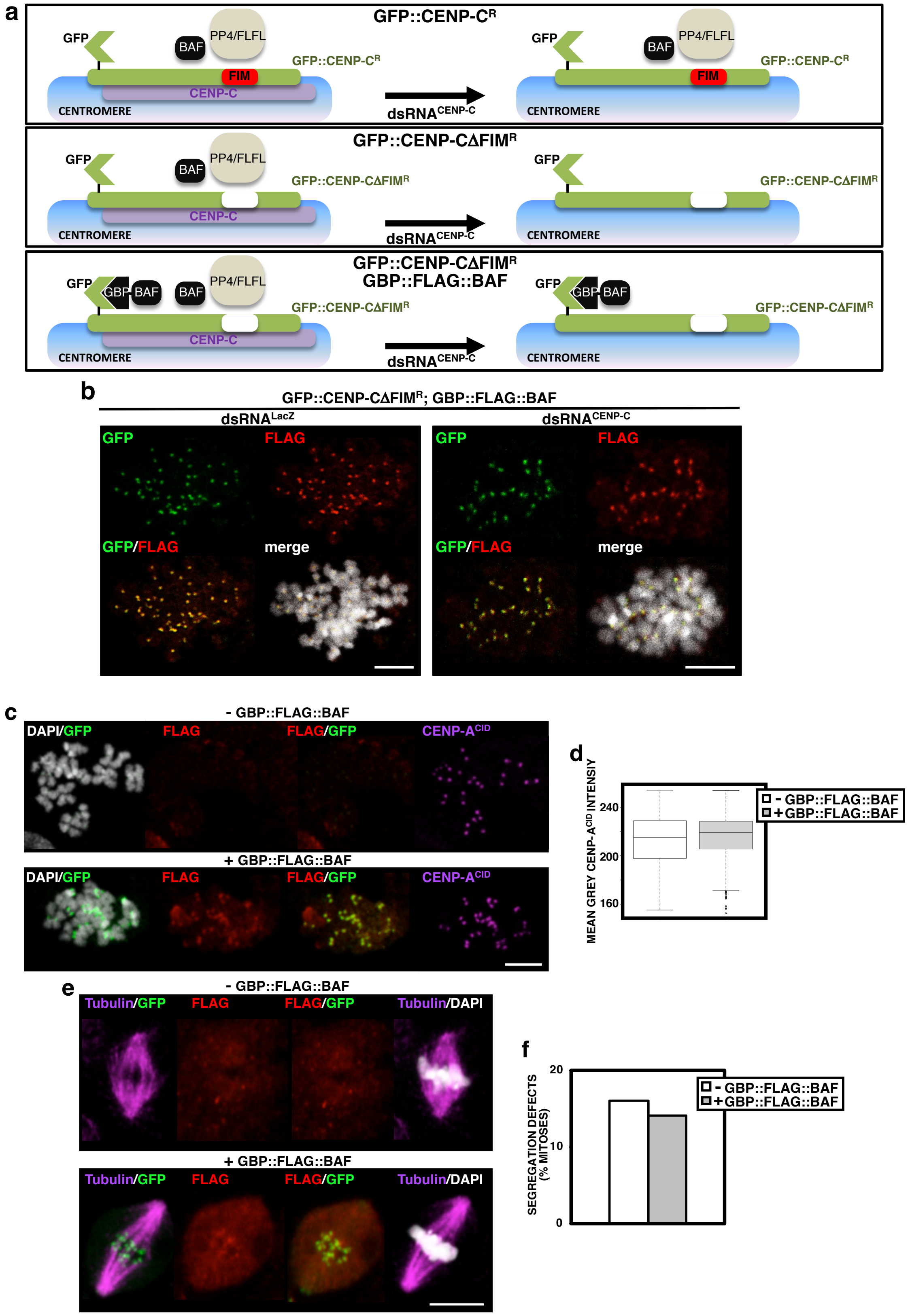

g

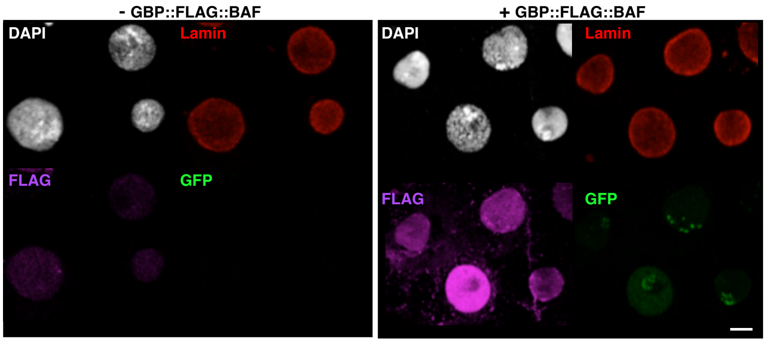

h

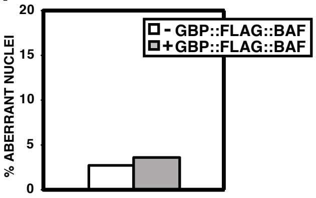

Supplementary Figure 9. **Constitutive targeting of BAF to centromeres**. (**a**) Schematic representation of these experiments. The effects of CENP-C depletion on centromeric localization of BAF and Flfl/PP4 is presented for GFP::CENP-C^R-expressing cells (top) and cells expressing GFP::CENP-CΔFIM^R cells together with GBP::FLAG::BAF (bottom) or not (center). The FIM domain is indicated in red. Endogenous CENP-C is also indicated in magenta. (**b**) Immunostainings with αFLAG antibodies (red) are presented for mitotic chromosomes from dsRNA^{CENP-C} (right) and control dsRNA^{LacZ} (left) cells expressing GFP::CENP-CΔFIM^R and GBP::FLAG::BAF. GFP signals are direct fluorescence. DNA was stained with DAPI. Scale bars correspond to 5μm. (**c**) Immunostainings with αFLAG (red) and αCENP-A^{CID} (magenta) antibodies are presented for mitotic chromosomes from GFP::CENP-C^R cells expressing GBP::FLAG::BAF (bottom) or not (top). GFP signal is direct fluorescence. DNA was stained with DAPI. Scale bar corresponds to 5μm. (**d**) Quantitative analysis of the results shown in **c**. The mean grey values per centromere of αCENP-A^{CID} fluorescence are shown for GFP::CENP-C^R cells expressing GBP::FLAG::BAF or not. (n= 1; N>451). (**e**) Metaphase figures from GFP::CENP-C^R cells expressing GBP::FLAG::BAF (bottom) or not (top). Immunostainings with αFLAG (green) and αTubulin (magenta) antibodies are shown. GFP signal is direct fluorescence. DNA was stained with DAPI. Scale bar corresponds to 5μm. (**f**) Quantitative analysis of the results shown in **e**. The percentage of segregation defects are presented for GFP::CENP-C^R cells expressing GBP::FLAG::BAF or not (n= 1; N>18). (**g**) Immunostainings with αLaminB antibodies (red) and αFLAG (magenta) antibodies are presented for GFP::CENP-C^R cells expressing GBP::FLAG::BAF (right) or not (left). GFP signal (green) is direct fluorescence. DNA was stained with DAPI. Scale bar

corresponds to 5μm. (**h**) Quantitative analysis of the results shown in **g**. The percentage of cells showing altered NE morphology is presented for GFP::CENP-C^R cells expressing GBP::FLAG::BAF or not (n= 1; N>111).

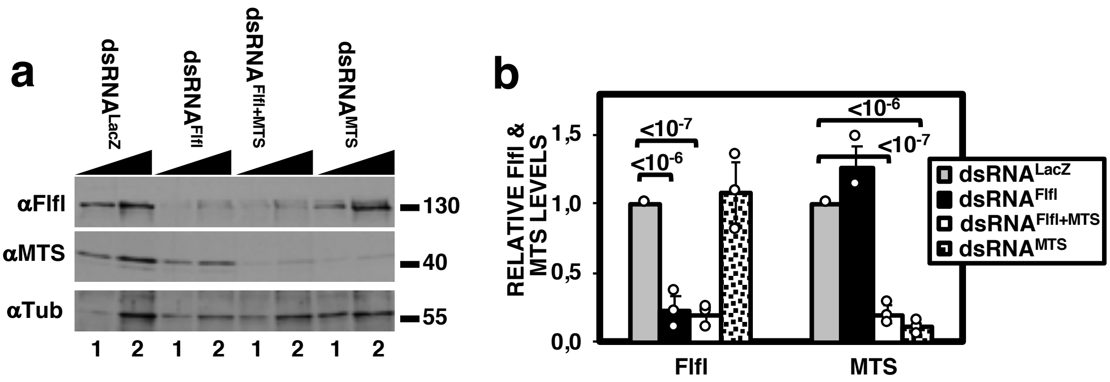

Supplementary Figure 10. Efficiency of the knockdown shown in Figs. 5a and 5b. (**a**) Increasing amounts of extracts (lanes 1 and 2) prepared from control $dsRNA^{LacZ}$ cells and from cells treated with dsRNA against Flfl ($dsRNA^{Flfl}$), MTS ($dsRNA^{MTS}$), and both Flfl and MTS ($dsRNA^{Flfl+MTS}$) are analyzed by WB using αFlfl, αMTS. αTubulin antibodies were used for loading control. The position of MW markers (in kDa) is indicated. (**b**) Quantitative analysis of the results shown in **a**. The relative Flfl and MTS levels are presented for the indicated RNAi-treated cells. Results are the average of 3 independent experiments (error bars are SD; two-tailed t-test, p-values are indicated).

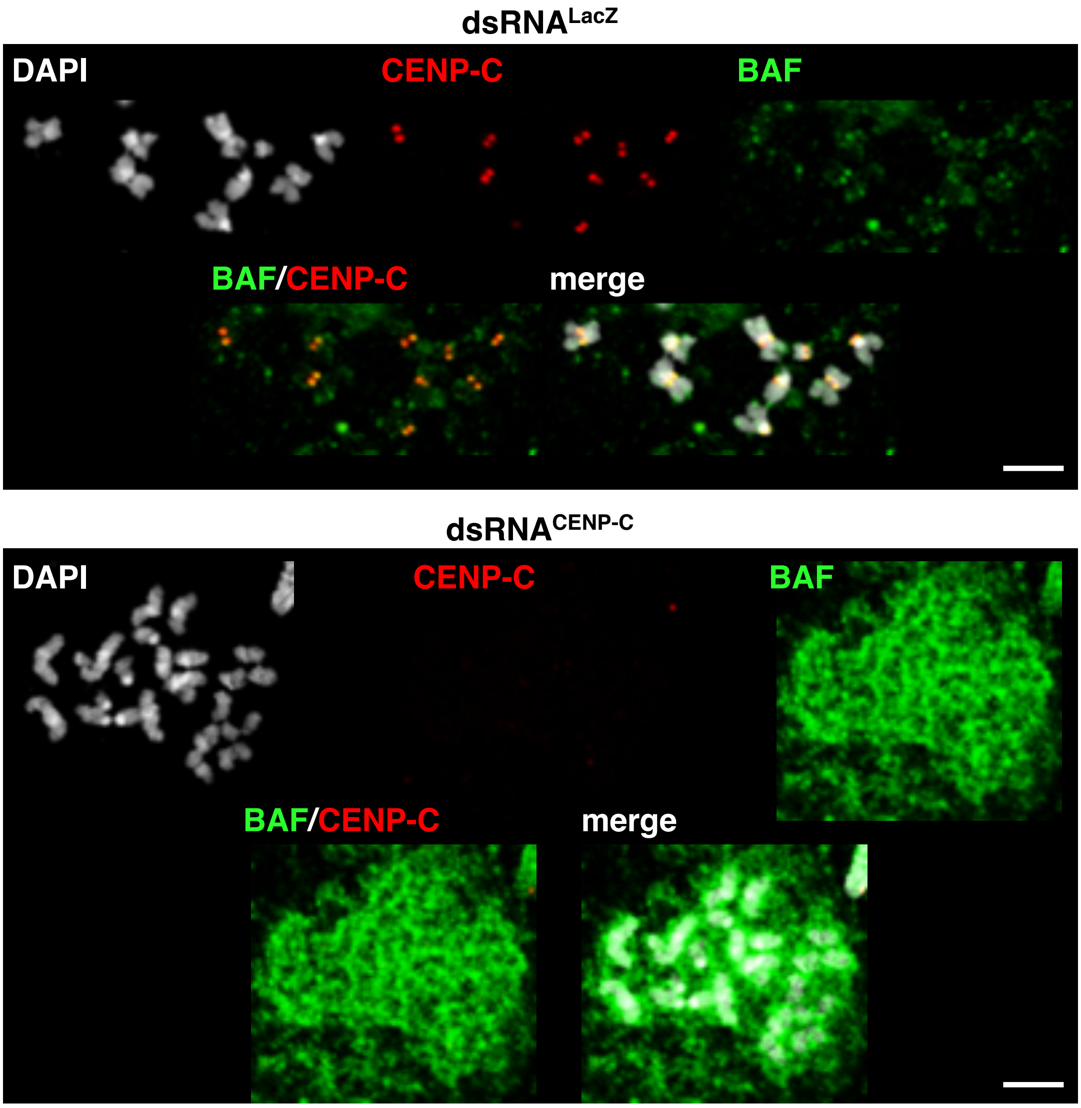

Supplementary Figure 11. CENP-C depletion induces the accumulation of perichromosomal BAF. Immunostainings with αBAF (green) and αCENP-C antibodies (red) are presented for mitotic chromosomes from dsRNA$^{CENP\text{-}C}$ (bottom) and control dsRNA^{LacZ} (top) cells. DNA was stained with DAPI. Scale bars correspond to 5μm.

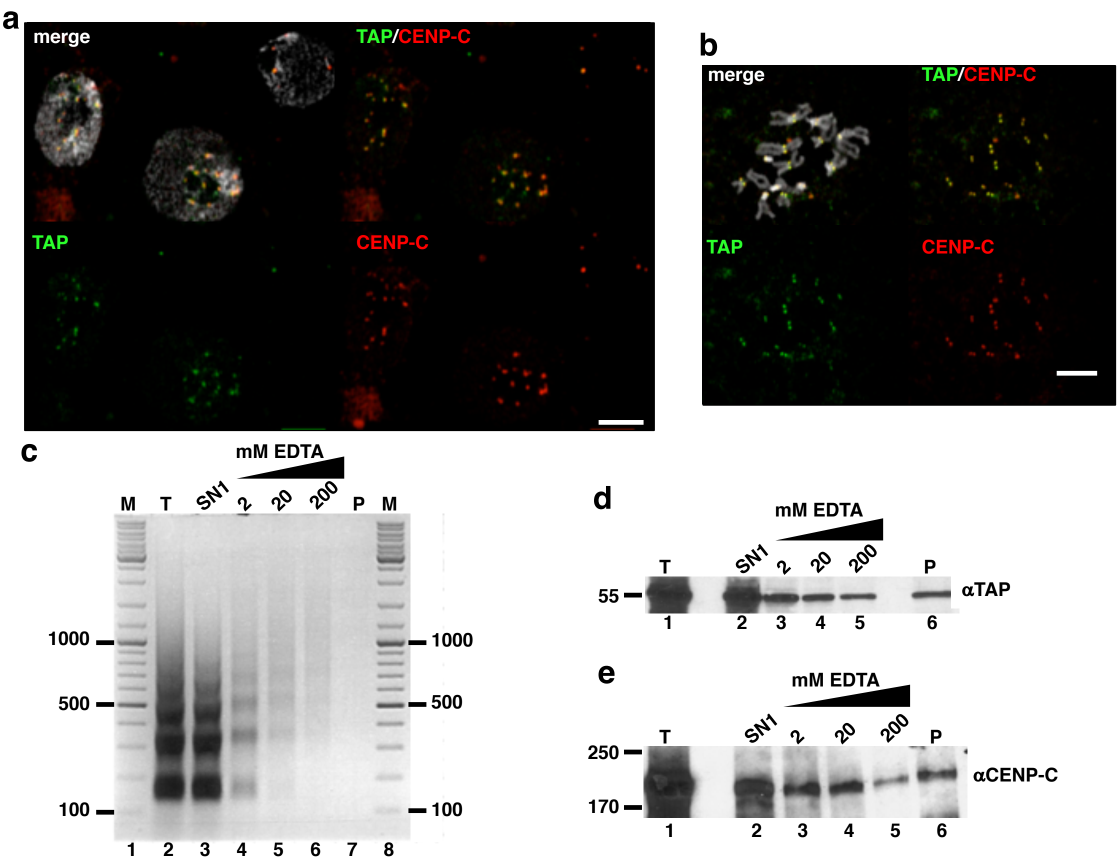

Supplementary Figure 12. **Preparation of CENP-A^{CID} enriched chromatin.** (**a** and **b**) Stable S2 cells expressing CENP-A^{CID}::TAP are stained with αTAP antibodies (green) and αCENP-C (red) antibodies in interphase (**a**) and mitosis (**b**). DNA was stained with DAPI. Scale bars are 5μm. (**c**) Agarose gel electrophoretic analysis of total DNA fragments produced after MNase digestion of purified nuclei prepared from stable S2 cells expressing CENP-A::^{CID}-TAP (T) (lane 2) and those contained in fraction SN1 (lane 3), released at increasing EDTA concentration (lanes 4-6) or retained in the insoluble pellet (P) (lane 7) (see **Methods** for details). Lanes 1 and 8 correspond to molecular weight markers (M). The size in bp of selected markers is indicated. (**d** and **e**) CENP-A^{CID}::TAP and CENP-C content of the indicated fractions is determined by WB using αTAP (**d**) and αCENP-C (**e**) antibodies. The position of MW markers (in kDa) is indicated.

Figure 1d

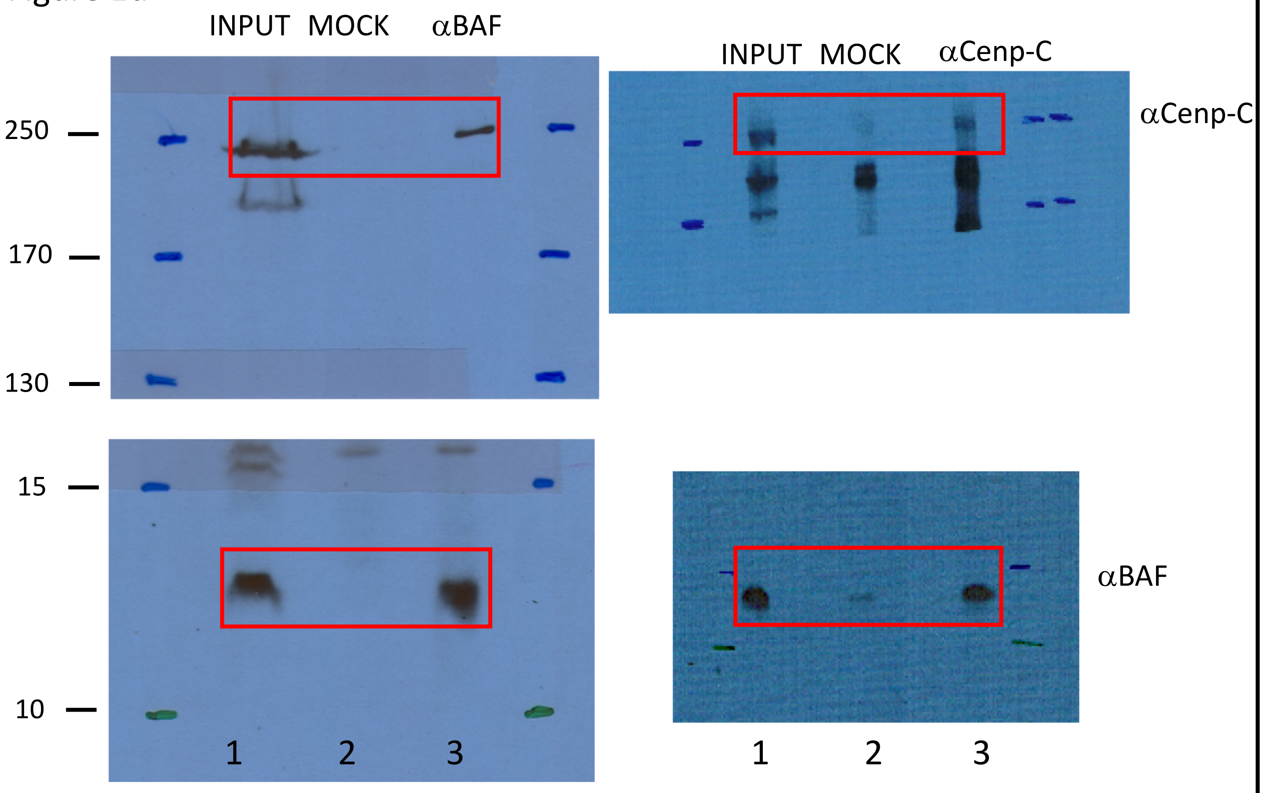

INPUT MOCK αBAF
250
170
130
15
10
1 2 3
INPUT MOCK αCenp-C
αCenp-C
αBAF
1 2 3

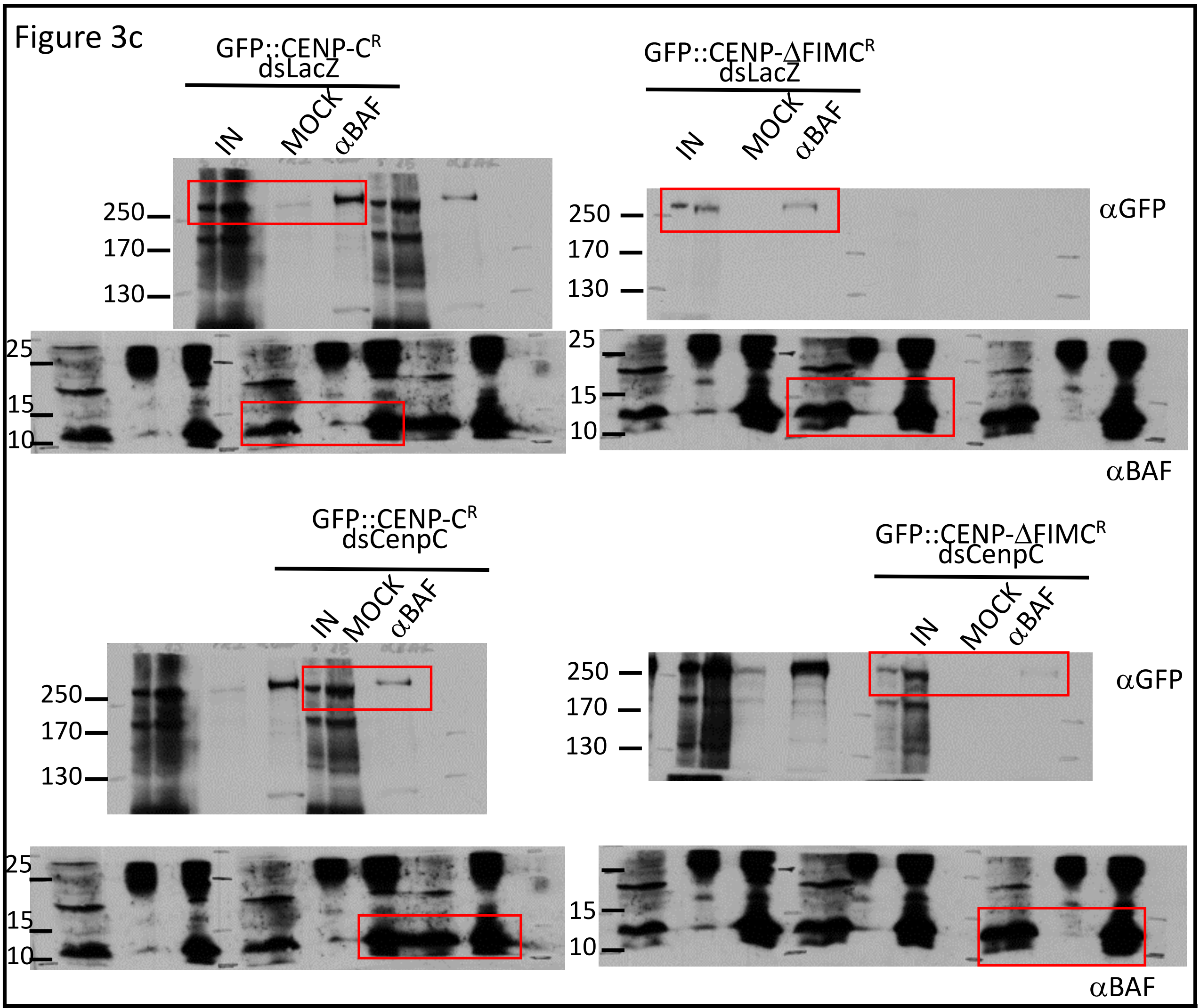
Figure 3c
GFP::CENP-CR dsLacZ
IN
MOCK
αBAF
GFP::CENP-ΔFIMCR dsLacZ
250
170
130
25
15
10
αGFP
αBAF
GFP::CENP-CR dsCenpC
GFP::CENP-ΔFIMCR dsCenpC

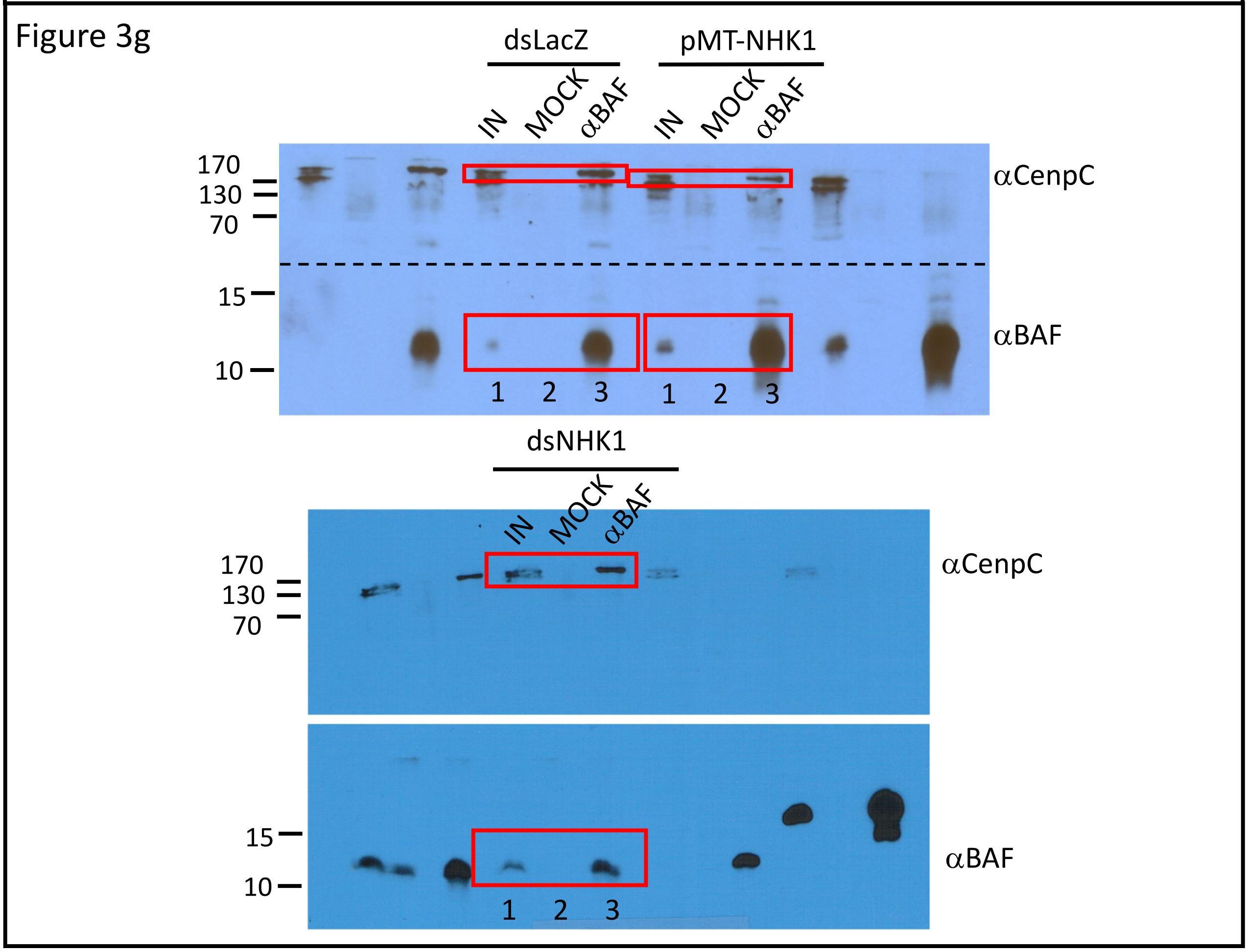
Figure 3g
dsLacZ
pMT-NHK1
IN
MOCK
αBAF
170
130
70
15
10
αCenpC
αBAF
1
2
3
dsNHK1

Figure 5e

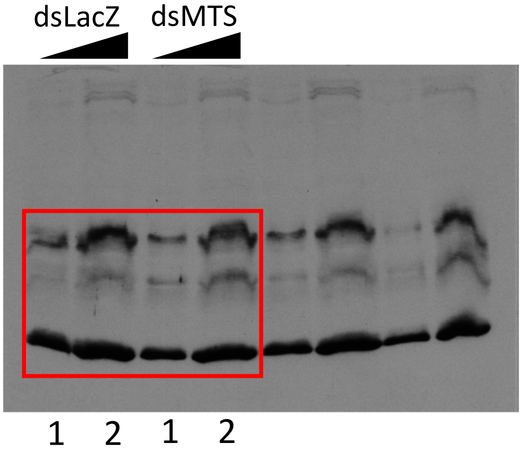

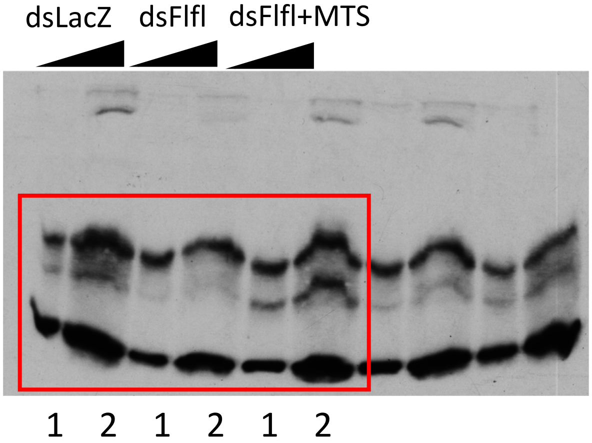

Supplementary Figure 1a

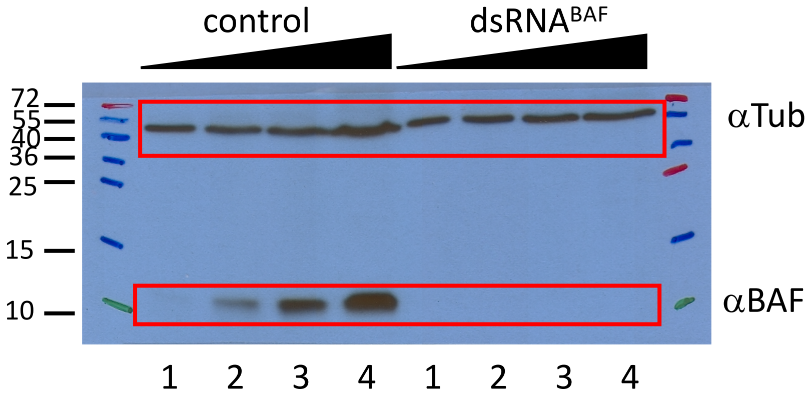

Supplementary Figure 2a

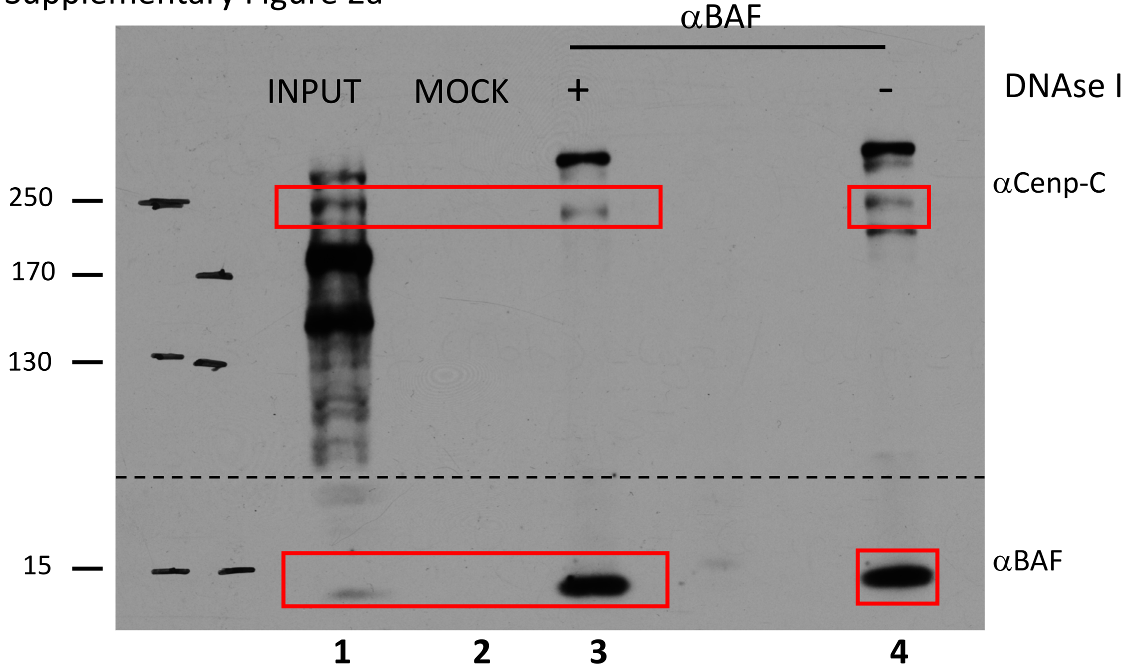

Supplementary Figure 2b

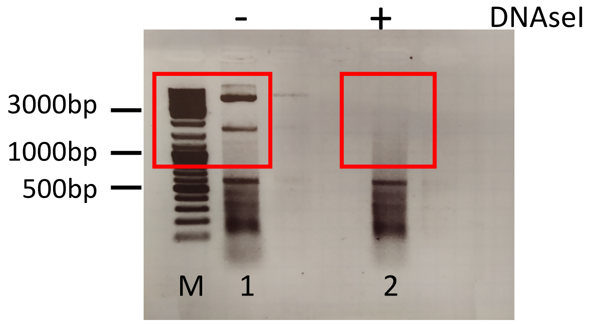

Supplementary Figure 2d

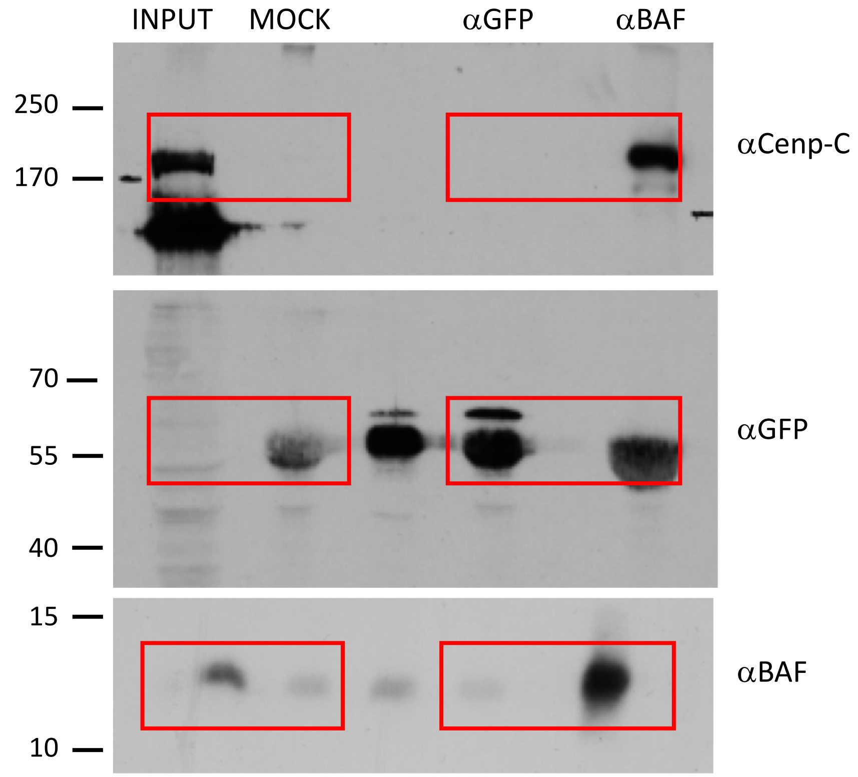

Supplementary Figure 3f

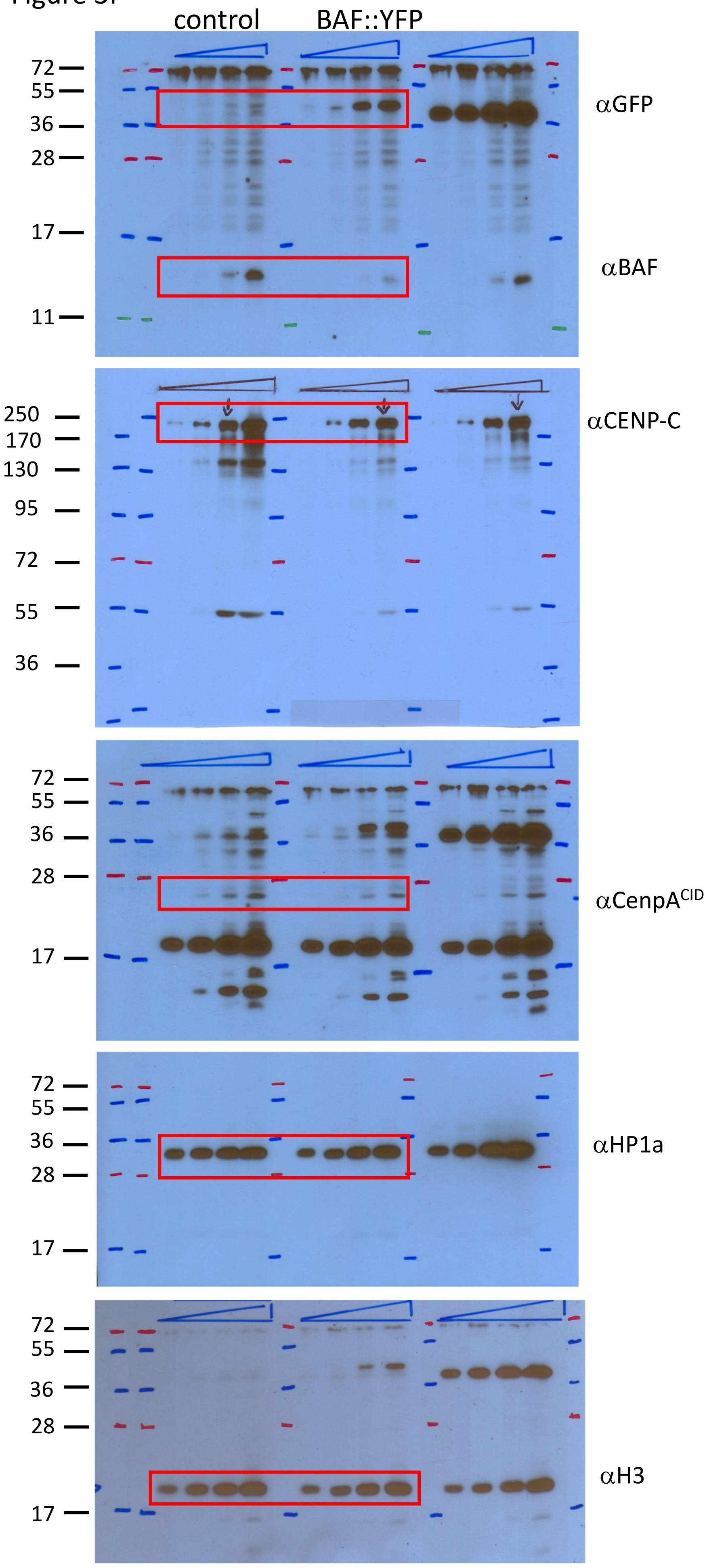

Supplementary Figure 5a

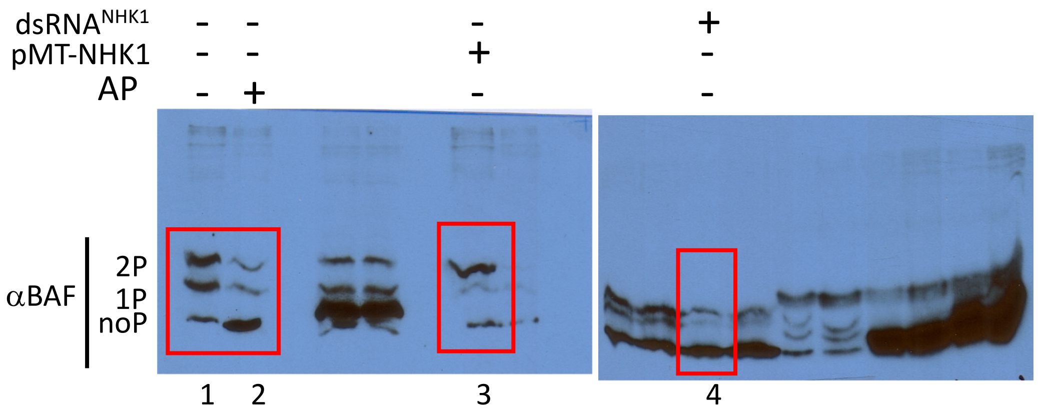

Supplementary Figure 5b

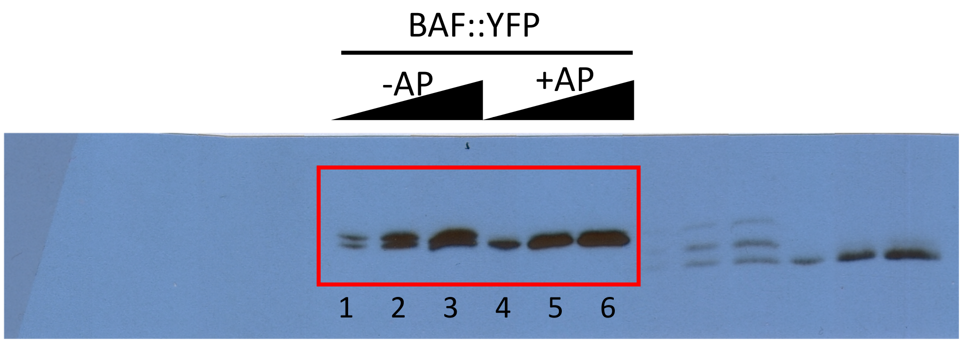

Supplementary Figure 5d

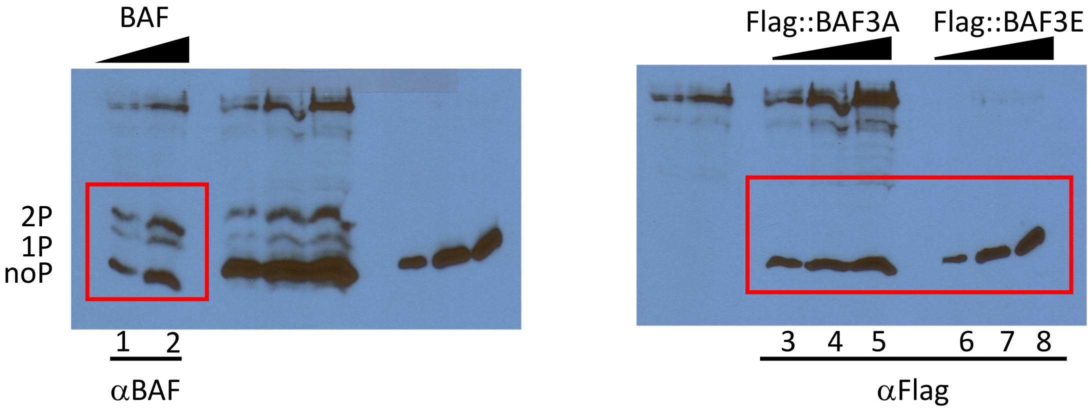

Supplementary Figure 5e

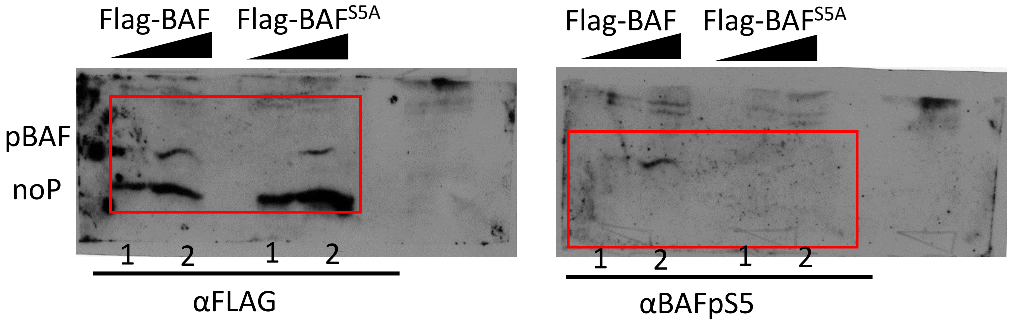

Supplementary Figure 6c

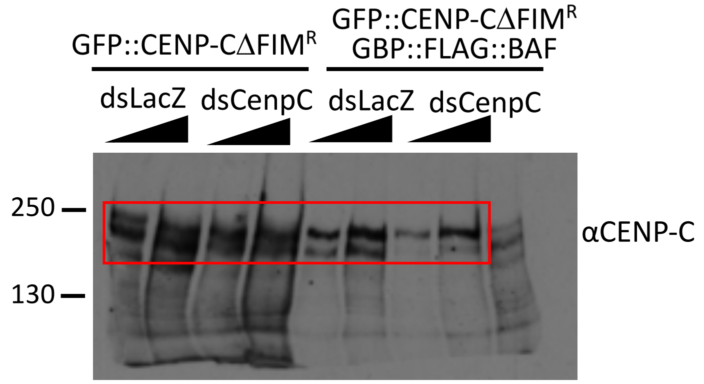

Supplemental Figure 7a

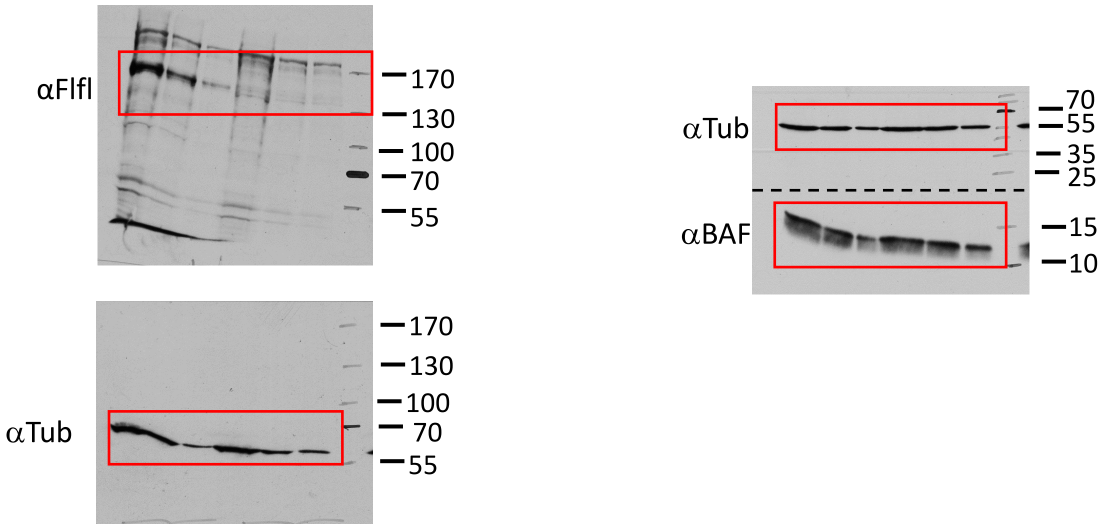

Supplemental Figure 7e

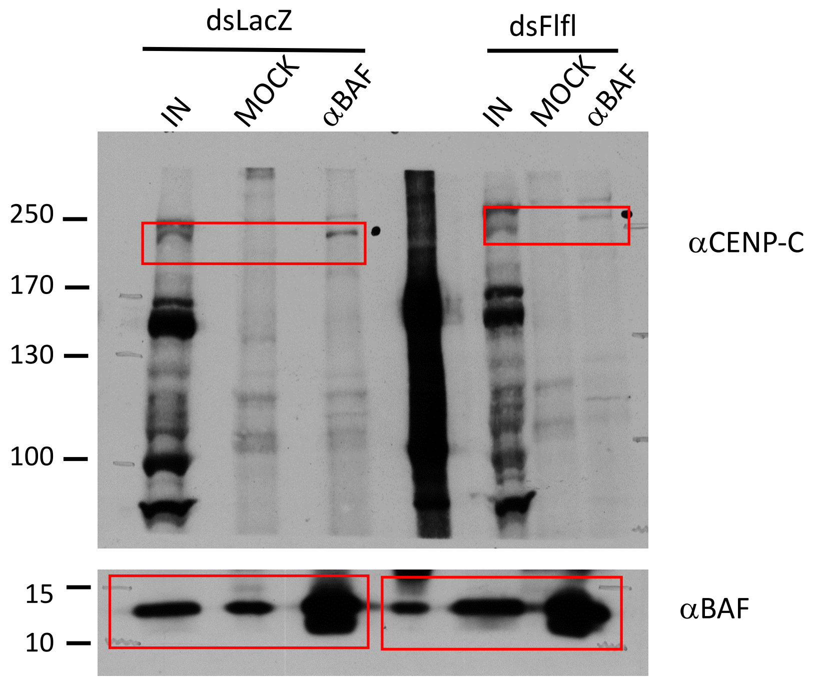

Supplementary Figure 8a

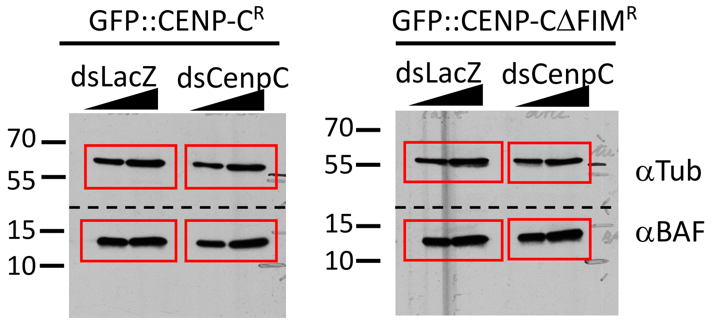

Supplementary Figure 10a

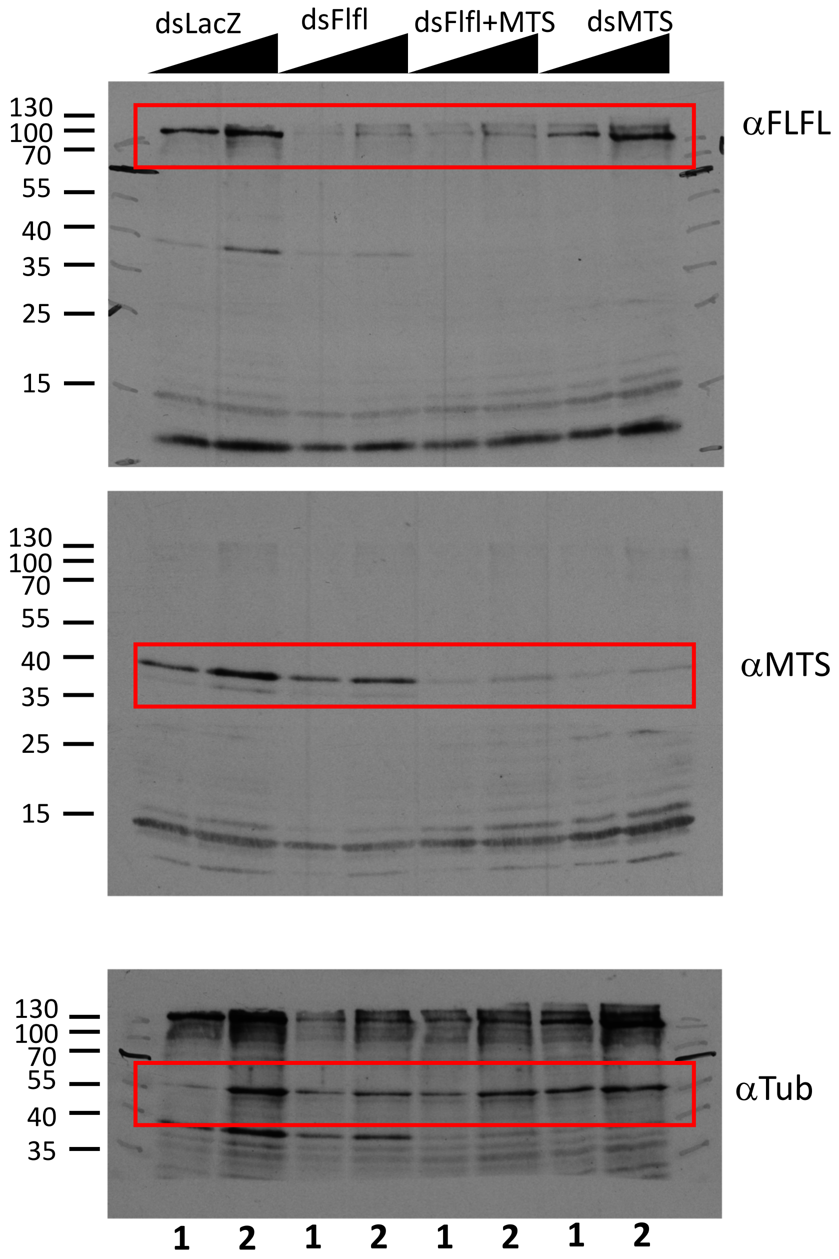

Supplementary Figure 12c

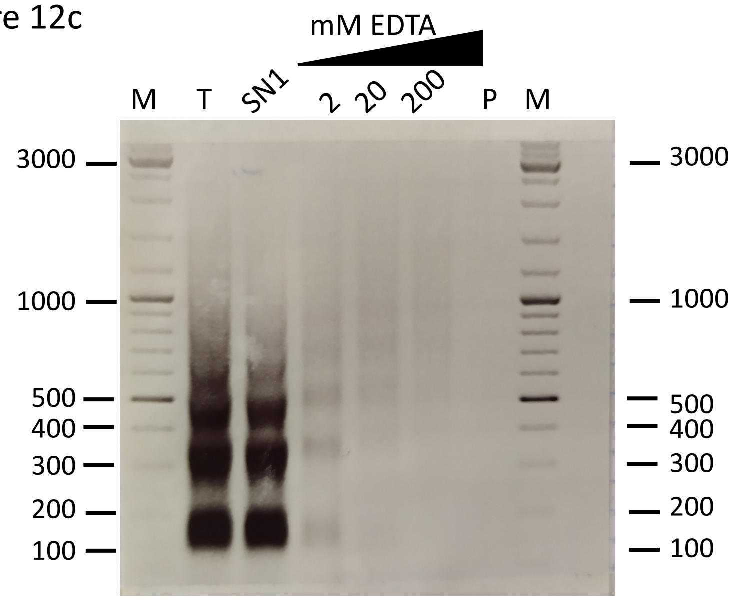

Supplementary Figure 12d

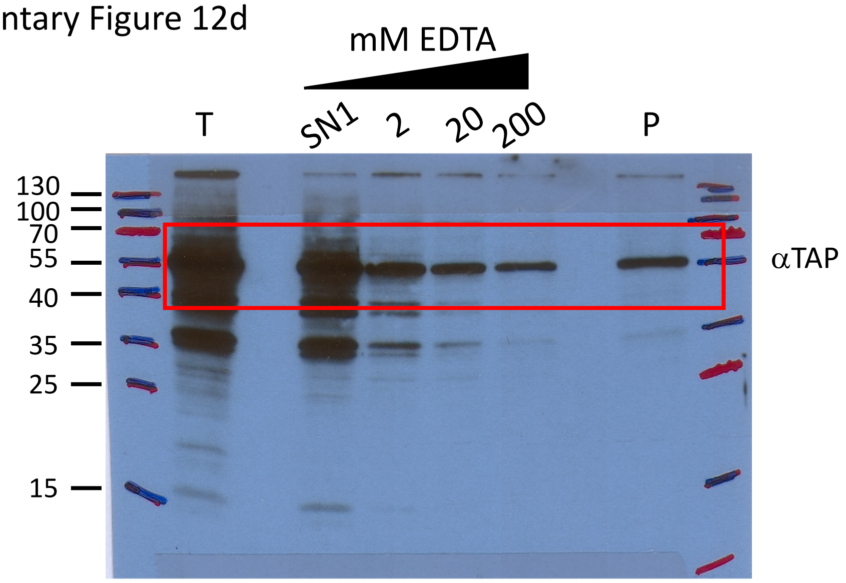

Supplementary Figure 12e

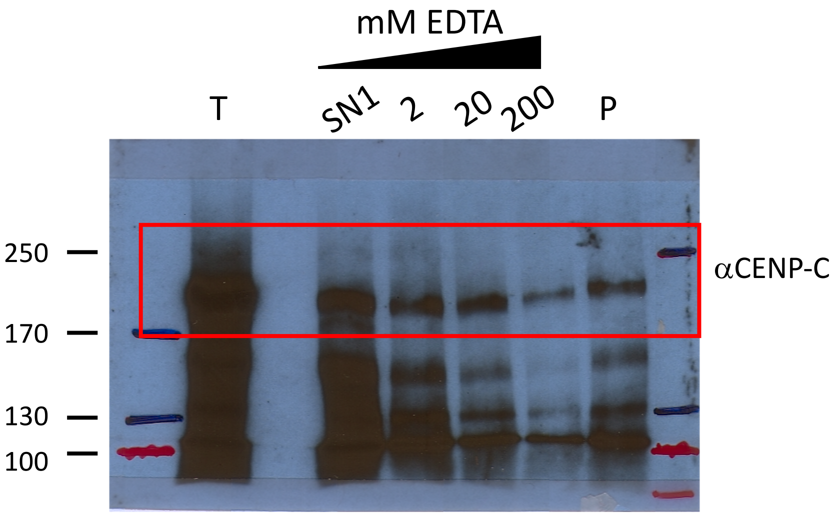

Supplementary Figure 13. Uncropped images of the WBs and DNA gels presented in the main figures and the rest of supplementary figures.

SUPPLEMENTARY TABLES

Supplementary Table 1. List of proteins co-purifying with CENP-A^{CID} enriched chromatin identified by MS**.**

Name	UniProt	MW [KDa]	Mascot Score	Coverage [%]	Number of peptides identified
**Histone H4**	P84040	11.4	594.5	48.5	11
**Histone H2B**	P02283	13.7	422.5	45.5	8
**CENP-A^{CID}**	Q9V6Q2	26.0	303.1	38.2	6
**Porin, isoform A**	Q94920	30.5	273.4	24.5	6
**Lamin Dm0**	P08928	71.2	236.8	13.5	4
**Actin**	P02572	41.8	197.1	17.8	4
**Histone H3**	P02299	15.4	182.2	22.1	4
**Histone H2Av**	P08985	15.0	174.5	24.1	3
**Histone H2A**	P84051	13.4	167.6	25.8	2
**ATP synthase subunit d, Mitochondrial**	Q24251	20.2	127.3	29.2	3
**l(2)03709, isoform C**	Q0E924	33.0	125.1	7.4	2
**CG30122, isoform B**	A1ZBB4	140.4	120.4	3.0	2
**Barrier to autointegration factor (BAF)**	Q9VLU0	10.1	97.0	16.7	1
**Hrp48.1**	P48809	41.0	91.8	4.2	1
**ADP/ATP translocase**	Q26365	32.9	90.4	5.7	2
**Scully protein**	O18404	26.9	90.2	5.9	1
**Protein l(2)37 Cc**	P24156	30.4	89.9	9.1	1
**Protein no-on-transient A**	Q04047	77.0	82.5	2.7	2
**CG8289**	Q9VX35	37.4	74.0	7.1	1
**Bellwether**	P35381	59.4	69.9	2.2	1
**60S Ribosomal protein L22**	P50887	32.3	69.4	3.8	1
**ATP synthase subunit beta**	Q05825	53.5	62.1	4.2	2
**Glorund, isoform A**	Q9VGH5	61.4	61.9	1.7	1
**Heat shock protein 60A**	O02649	60.8	61.8	6.1	2
**Heat shock 70KDa protein cognate 5**	P29845	74.2	59.2	1.7	1
**Phosphate transporter**	Q9XZE4	38.9	57.7	3.4	1
**CG11999**	Q9VNA3	23.6	56.1	5.6	1
**Signal sequence receptor subunit beta**	Q9VUZ0	21.2	47.4	12.6	1
**Shrub**	Q8T0Q4	25.4	43.4	3.1	1

**CG5787**	Q9VK59	100.2	40.9	1.9	1
**Calreticulin**	Q9U916	46.8	39.9	6.2	1
**Serine/Threonine-protein kinase TOR**	Q9VK45	280.9	38.6	0.3	1
**CG4169**	Q9VV75	45.4	37.4	4.5	1
**Hrb98DE**	P07909	39.0	17.1	13.7	5
**Su(var)205**	P05205	23.2	13.6	19.4	3
**Chromosomal protein D1**	P22058	37.0	5.8	8.5	2
**ATP-dependent RNA helicase p62**	P19109	78.5	5.5	2.1	1
**ATP Synthase subunit gamma, mitochondrial**	O01666	32.9	4.4	3.7	1
**NHP2-like protein 1**	Q9U3Z7	13.9	2.6	9.45	1
**Protein lingerer**	Q86S05	139.4	2.3	0.87	1
**Ribosomal protein L28**	Q9VZS5	16.0	2.2	5.6	1
**Elongation Factor 1-alpha 2**	P05303	50.6	2.0	2.3	1
**Circadian clock controlled protein**	Q24764	29.3	2.0	3.1	1

Supplementary Table 2. Primers for synthesis of dsRNAs used in RNAi knockdown experiments and for cloning.

dsLacZ-Fw	TAATACGACTCACTATAGGGATGACCATGATTACGC CAAGC
dsLacZ-Rv	TAATACGACTCACTATAGGGCAATTTCCATTCGCCA TTCAG
dsBAF-Fw	TAATACGACTCACTATAGGGATGTCGGGCACATCG CAGAAACACAGG
dsBAF-Rv	TAATACGACTCACTATAGGGTTACAAGAACTCCTCG CACCAATCGTT
dsCENP-C1-Fw	TAATACGACTCACTATAGGGGCACACCTTGCACAG AGAAA
dsCENP-C1-Rv	TAATACGACTCACTATAGGGTGTAGGTACGTCGTAG GCCC
dsCENP-C2-Fw	TAATACGACTCACTATAGGGTGGTAAACTATTTGGG TCTCTC
dsCENP-C2-Rv	TAATACGACTCACTATAGGGGGTACCAGTTCGTTCT CCATCG
dsCENP-C^R-Fw[(1)]	TAATACGACTCACTATAGGGAGAGGTACCACCTCCT ATCGAATA
dsCENP-C^R-Rv[(1)]	TAATACGACTCACTATAGGGAGAGAATTCCAATTTG GATCTGGA
dsFlfl1-Fw	TAATACGACTCACTATAGGGAGAATGACGACTGAC ACCCGC
dsFlfl1-Rv	TAATACGACTCACTATAGGGAGAACAACTTTTCCTT TCGCA
dsMTS-Fw	TAATACGACTCACTATAGGGGCCAAGGAGATTCTCT CC
dsMTS-Rw	TAATACGACTCACTATAGGGCTGGCCAAAGGTGTA ACC
dsNhk1.1-Fw	TAATACGACTCACTATAGGGACCCCAAGAAGATGC ATAATGGCACC
dsNhk1.1-Rv	TAATACGACTCACTATAGGGTTTTTTCGTCCAGTGA TGAGTTCAAGACT
dsNHK1.2-Fw	TAATACGACTCACTATAGGGAGTTCACCACCCAGC CAGAAGCGC
dsNHK1.2-Rv	TAATACGACTCACTATAGGGGCTGGTGTCCGCGGA CTACGGCCGGG
GBPSacI-Fw[(2)]	TCGGAGCTCATGGCTCAGGTGCAGCTG
GBPSacI-Rv[(2)]	GCGGAGCTCAATGAGGAGACGGTGACCTG

(1) used in the experiments in which endogenous CENP-C was depleted in cells expressing RNAi-resistant CENP-C constructs
(2) used for cloning the GBP::FLAG::BAF construct
