# Supplementary material for: A fraction of barrier-to-autointegration factor (BAF) associates with centromeres and controls mitosis progression
Source: Commun Biol. 2020 Aug 19;3:454. doi: 10.1038/s42003-020-01182-y (PMC7438335; doi:10.1038/s42003-020-01182-y)
Supplement: Supplementary file 2 — Description of Additional Supplementary Files [file 42003_2020_1182_MOESM2_ESM.pdf]

Description of Additional Supplementary Files

File Name: **Supplementary Data 1**

Description Source Data underlying graphs presented in the main and supplementary Figures

File Name: **Supplementary Movie 1**

Description Normal mitosis from control CENP-C depleted CENP-CR::GFP cells expressing Nup-107::mRFP to label the NE. Maximum intensity projections of three and all z-stacks are shown for transmitted light (left panel) and merged fluorescence images (right panel), respectively.

File Name: **Supplementary Movie 2**

Description Mitosis from CENP-C depleted CENP-CDFIMR::GFP cells expressing Nup-107::mRFP to label the NE showing increased total duration, as well as from NEBD to AO and from AO to NER. Maximum intensity projections of three and all z-stacks are shown for transmitted light (left panel) and merged fluorescence images (right panel), respectively.

File Name: **Supplementary Movie 3**

Description Aberrant mitosis from CENP-C-depleted CENP-CDFIMR::GFP cells expressing Nup-107::mRFP to label the NE showing increased mitosis duration and persistent Nup-107::mRFP during division, giving rise to daughter cells with abnormal nuclear morphology. Maximum intensity projections of three and all zstacks are shown for transmitted light (left panel) and merged fluorescence images (right panel), respectively.

File Name: **Supplementary Movie 4**

Description Aberrant mitosis from CENP-C-depleted CENP-CDFIMR::GFP cells expressing Nup-107::mRFP to label the NE showing increased mitosis duration and persistent Nup-107::mRFP during division, giving rise to a multinucleated cell. Maximum intensity projections of three and all z-stacks are shown for transmitted light (left panel) and merged fluorescence images (right panel), respectively

File Name: **Supplementary Movie 5**

Description In vivo time-lapse recording of a CENP-C-depleted CENPCDFIMR::GFP cell expressing Nup-107::mRFP to label the NE showing two nuclei. Maximum intensity projections of three and all z-stacks are shown for transmitted light (left panel) and merged fluorescence images (right panel), respectively.

File Name: **Supplementary Movie 6**

Description In vivo time-lapse recording of a CENP-C-depleted CENPCDFIMR::GFP cell expressing Nup-107::mRFP to label the NE showing two nuclei. Maximum intensity projections of three and all z-stacks are shown for transmitted light (left panel) and merged fluorescence images (right panel), respectively.
