# Supplementary material for: A fraction of barrier-to-autointegration factor (BAF) associates with centromeres and controls mitosis progression
Source: Commun Biol. 2020 Aug 19;3:454. doi: 10.1038/s42003-020-01182-y (PMC7438335; doi:10.1038/s42003-020-01182-y)
Supplement: Supplementary file 10 — Reporting Summary [file 42003_2020_1182_MOESM10_ESM.pdf]

nature research

Corresponding author(s): F. Azorin
COMMSBIO-19-1690A

Last updated by author(s): Jul 6, 2020

Reporting Summary

Nature Research wishes to improve the reproducibility of the work that we publish. This form provides structure for consistency and transparency in reporting. For further information on Nature Research policies, see Authors & Referees and the Editorial Policy Checklist.

Statistics

For all statistical analyses, confirm that the following items are present in the figure legend, table legend, main text, or Methods section.

n/a	Confirmed	
☐	☒	The exact sample size (n) for each experimental group/condition, given as a discrete number and unit of measurement
☐	☒	A statement on whether measurements were taken from distinct samples or whether the same sample was measured repeatedly
☐	☒	The statistical test(s) used AND whether they are one- or two-sided<br>*Only common tests should be described solely by name; describe more complex techniques in the Methods section.*
☒	☐	A description of all covariates tested
☒	☐	A description of any assumptions or corrections, such as tests of normality and adjustment for multiple comparisons
☒	☐	A full description of the statistical parameters including central tendency (e.g. means) or other basic estimates (e.g. regression coefficient) AND variation (e.g. standard deviation) or associated estimates of uncertainty (e.g. confidence intervals)
☐	☒	For null hypothesis testing, the test statistic (e.g. F, t, r) with confidence intervals, effect sizes, degrees of freedom and P value noted<br>*Give P values as exact values whenever suitable.*
☒	☐	For Bayesian analysis, information on the choice of priors and Markov chain Monte Carlo settings
☒	☐	For hierarchical and complex designs, identification of the appropriate level for tests and full reporting of outcomes
☒	☐	Estimates of effect sizes (e.g. Cohen's d, Pearson's r), indicating how they were calculated

Our web collection on statistics for biologists contains articles on many of the points above.

Software and code

Policy information about availability of computer code

Data collection	Described in Methods section
Data analysis	Described in Methods section

For manuscripts utilizing custom algorithms or software that are central to the research but not yet described in published literature, software must be made available to editors/reviewers. We strongly encourage code deposition in a community repository (e.g. GitHub). See the Nature Research guidelines for submitting code & software for further information.

Data

Policy information about availability of data

All manuscripts must include a data availability statement. This statement should provide the following information, where applicable:

- Accession codes, unique identifiers, or web links for publicly available datasets
- A list of figures that have associated raw data
- A description of any restrictions on data availability

The data supporting the findings of this study are available within the article and its supplementary materials. A statement of data availability is included in the manuscript

Field-specific reporting

Please select the one below that is the best fit for your research. If you are not sure, read the appropriate sections before making your selection.

☒ Life sciences ☐ Behavioural & social sciences ☐ Ecological, evolutionary & environmental sciences

Life sciences study design

All studies must disclose on these points even when the disclosure is negative.

Sample size	Sample size was determined to be adequate based on the magnitude and consistency of measurable differences between groups
Data exclusions	Data were not excluded from analysis
Replication	All immunostaining experiments were done 3 or more times with at least two independent slides in each condition and experiment
Randomization	Randomization of experiments was not required
Blinding	No blinding was done in this study. All the data are quantitative and measurements are made using a microscope and a software for the analysis, being not easily subject to operator bias

Reporting for specific materials, systems and methods

We require information from authors about some types of materials, experimental systems and methods used in many studies. Here, indicate whether each material, system or method listed is relevant to your study. If you are not sure if a list item applies to your research, read the appropriate section before selecting a response.

Materials & experimental systems

n/a	Involved in the study	
☐	☒	Antibodies
☐	☒	Eukaryotic cell lines
☒	☐	Palaeontology
☒	☐	Animals and other organisms
☒	☐	Human research participants
☒	☐	Clinical data

Methods

n/a	Involved in the study	
☒	☐	ChIP-seq
☒	☐	Flow cytometry
☒	☐	MRI-based neuroimaging

Antibodies

Antibodies used

Primary antibodies
Rabbit polyclonal antiBAF and rat and rabbit polyclonal antiCENP-C antibodies are described in the Methods of the present study.
anti-pBAF (human antibody described in Zhuang et al 2014)
antiCENP-ACID (rabbit polyclonal antibody previously described in Moreno-Moreno et al., 2006)
antiHP1a and antiHP1c (rat polyclonal antibodies described in Font-Burgada et al., 2008)
antiFlfl (rat polyclonal antibody described in Lipinszki et al., 2015)
antiMTS (BD-Transduction Laboratories, 610555),
rabbit purified antiFLAG (Sigma F7425)
mouse monoclonal antiFLAG (Sigma F3165),
mouse monoclonal antiTubulin (Millipore, MAB3408)
antiTAP (Open Biosystems, CAB1001)
antiLaminB (mouse monoclonal antibody DSHB ADL67.10)
Secondary antibodies:
Cy2 AffiniPure Goat anti-rabbit IgG (H+L) JACKSON (111-225-144)
Cy2 AffiniPure Goat anti-rat IgG (H+L) JACKSON (111-225-143)
Cy5 AffiniPure Goat anti-rat IgG (H+L) JACKSON (112-175-143)
Cy5 AffiniPure Goat anti-rabbit IgG (H+L) JACKSON (112-175-144)
Peroxidase AffiniPure Donkey anti-rat IgG (H+L) JACKSON (712-035-150)
Peroxidase AffiniPure Goat anti-rabbit IgG (H+L) JACKSON (111-035-144)
Peroxidase AffiniPure Donkey anti-mouse IgG (H+L) JACKSON (715-035-150)

Validation

antiBAF and antCENP-C were validated in the present study.
antBAFpS5 was validated for drosophila in the present study.

Eukaryotic cell lines

Policy information about cell lines

Cell line source(s)	Schneider's Drosophila Line 2 [D. Mel. (2), SL2] (ATCC® CRL-1963™)
Authentication	None of the cells have been authenticated

Mycoplasma contamination	Cell lines were not tested for mycoplasma contamination but no indication of contamination was observed
Commonly misidentified lines (See ICLAC register)	No commonly misidentified cell lines were used
