# Supplementary material for: A fraction of barrier-to-autointegration factor (BAF) associates with centromeres and controls mitosis progression
Source: Commun Biol. 2020 Aug 19;3:454. doi: 10.1038/s42003-020-01182-y (PMC7438335; doi:10.1038/s42003-020-01182-y)
Supplement: Supplementary file 11 — Peer Review File [file 42003_2020_1182_MOESM11_ESM.pdf]

Editorial Note: *Parts of this Peer Review File have been redacted as indicated to maintain the confidentiality of unpublished data*

Reviewers' comments:

Reviewer #1 (Remarks to the Author):

The manuscript entitled, "Centromeric control of nuclear envelope dynamics" by Torras-Llort et al. utilizes cytological methods combined with basic biochemical approaches to investigate the role of BAF in centromere assembly and mitotic progression. A previous study from this lab and others identified BAF as an interacting factor of the centromeric histone CENP-A. Here, the authors show that a small pool of BAF remains dephosphorylated during mitosis and localizes to centromeres (cenBAF), and this cenBAF is essential for CENP-C centromeric localization and normal mitotic progression. Using phoso-gel electrophoresis and Co-IP assays, the authors go on to show that cenBAF remains dephosphorylated during mitosis, and this phosphorylation status is regulated by the PP4 subunit Flfl through interaction with CENP-C. Deleting the Flfl-interacting motif of CENP-C (CENP-C∆FIM) disrupts cenBAF levels without disrupting total BAF levels, and reduces the interaction between BAF and CENP-C. The authors then show that the failure of cenBAF to localize to centromeres results in global BAF mislocalization during mitosis, and this mislocalization reflects impaired phosphorylation regulation. Finally, the authors use live imaging and standard IF to show that disrupting cenBAF localization using CENP-C∆FIM significantly increases the time between NEBD and anaphase onset, and leads to increased mitotic defects and abnormal nuclear morphology after mitosis.

The findings presented in this manuscript are very intriguing, and a better understanding of how centromeres influence nuclear envelope dynamics upon mitotic entry and exit is indeed of broad interest to both the centromere and nuclear organization fields. However, I feel that the title is misleading, as this study has in fact not revealed any novel information about how centromeres regulate nuclear envelope dynamics, as the title suggests. While BAF is indeed associated with the nuclear envelope during interphase, the fact that BAF is required for normal mitoses in Drosophila has previously been shown (see ref 17), and this study falls short of adding new insights to this aspect of this particular story. Additionally, the actual analyses of the nuclear envelope were minimal, and I found no data on "nuclear envelope dynamics". Despite these potential pitfalls, I still feel that there is novelty to the findings in this manuscript and that the link between BAF and CENP-C, and the mechanistic insights into this interaction, as well as the insights into how BAF affects mitotic progression and its role in centromere and kinetochore assembly – is very interesting and is highly relevant to many aspects of chromosome biology.

Therefore, it is my suggestion that the title be changed (see point 1 below), and extensive revisions to the manuscript and additional analyses of the existing data are done prior to acceptance for publication in Communications Biology.

Major concerns:
1. As stated above, I do not think this is an appropriate title for this manuscript, as "nuclear envelope dynamics" were not actually measured or quantified at any point, in my opinion. The pool of BAF being visualized and studied here is cenBAF, which is not associated with the nuclear envelope (as there is not NE during mitosis). The authors hypothesize that this cenBAF facilitates centromere association with the NE after mitosis to facilitate NER, but was it actually demonstrated that this cenBAF is essential for NER after mitosis? The mitotic delays found by live imaging were mostly after NEBD and before NER, and therefore seemingly independent of nuclear envelope dynamics (p.9). On p.13 of the manuscript in the discussion, the authors explicitly state that their data "suggest" a defect in NEBD. Nuclear envelope morphology defects do not equal nuclear envelope dynamics defects. Studies have

shown that chromosome missegregation itself is sufficient to cause nuclear blebbing and micronuclei formation. One way to directly address the dynamics of the nuclear envelope would be to better analyze the live imaging with the RFP-Nup107 (or even better, an RFP-Lamin construct) to see if indeed there are defects in nuclear envelope dynamics before or after mitosis when the BAF pathway is defective.

2. How many biological and/or technical replicates were done for each of these experiments? Ns are presented throughout the manuscript, but this seems to represent the number of cells analyzed for one replicate, and not how many replicates were performed.

3. On p. 7 of the text and figure 4A – how is this different from what is shown in figure 3A? On the right in 3A, we see the GFP-CENP-CΔFIM and BAF IF with control knockdown, which should look the same as middle of 4A, showing GFP-CENP-CΔFIM with BAF IF with no knockdown. Why is the perichromatin BAF only present in 4A? If this image was enhanced to emphasize this localization pattern, that's fine, but it needs to be indicated in the figure legend, and the original unenhanced image needs to be included in the supplemental data. If this image has NOT been enhanced, then again, why is there no perichromatin BAF in 3A?

4. This whole section starting on p.7 and ending on p.9 describing the results in figure 4 ("cenBAF prevents dephos..") was nearly impossible to follow and needs to be simplified.

5. In the discussion section on p.11, the "not shown" in vitro pull down data need to be shown and further discussed – how can the authors say that it is possible for BAF and Flfl to directly interact, when the experimental data for this interaction suggest otherwise?

6. Bottom of p.9, Figure 6 – The authors make several inferences here that are not entirely supported by the data, or at least are not the only possible explanation for the findings. Specifically, the result that these mitotic defects lead to increased ploidy – how did the authors come to this conclusion? Because of increased number of CENP-C foci? This could also be the result of defects in centromere clustering since S2 cells are already highly polyploid, and disrupted centromere clustering is known to happen in the subsequent interphase after defective chromosome segregation. Is ploidy inferred from nuclear volume? Again, this could also be the result of global chromatin decompaction. The reasoning behind these inferences is unclear and again, alternative possibilities need to be discussed. Directly testing ploidy would require doing mitotic chromosome spreads to show increased chromosome number (interphase DNA FISH would not be helpful in this case, as somatic homolog pairing in flies makes ploidy differences undetectable during interphase). Additionally, as described above, the defects in nuclear envelope morphology described here could all be an indirect result of general mitotic errors rather than a direct consequence of cenBAF disruption. This needs to be discussed.

Minor concerns/questions:

7. General issues with the figures:

o no statistical analyses included in the graphs of the figures or the figure legends, only the main text. Asterisks indicating significance should be added to the graphs.

o Graphs should all have their own letter assignment and not be "grouped" with the associated IF so that the figures are easier to follow and references made to figures in the text are clearer (for example, sometimes it is unclear if the authors are referencing an image or a graph if they are both designed 4A).

o The red/green coloring of all images is problematic for any colorblind readers. I suggest switching to magenta/cyan if possible.

8. Figure 2E – in the text (p.5) these data are further broken down into metaphase alignment defects and anaphase segregation defects. These data should be included in the main figure here, as the mechanisms behind these two types of mitotic defects are typically distinct. For example, metaphase misalignment is typically the result of defective kinetochore/microtubule attachments, while segregation errors in anaphase can happen for many reasons, including chromosome compaction defects and other non-centromeric defects. The fact that both of these types of defects are seen in the BAF knockdown is significant, and is important to support the authors' model for a role of BAF in

centromere/kinetochore assembly.
9. Figure 3B – the anti-BAF on this blot is very dirty and it's nearly impossible to see the bands. Do you have a cleaner blot you can include instead? The same is true for Figure S3D.
10. Figure 3C – why is the DAPI so saturated here? It's really hard to see where/if the IF is colocalized in the merged panel. The same is true for Figure S7B.
11. P. 7 and figure 4A – does the GBP-FLAG-BAF construct affect alone affect mitotic progression or centromere assembly?
12. P. 7,8, figure 4A,B – why do you think that this "perichromosomal" BAF is ectopically associated with chromatin? Wouldn't it co-localize with DAPI if it were chromatin bound? To me this seems like it could be more broadly nucleoplasmic.
13. P. 8 and figure 4B – the authors state that, "MTS depletion in Flfl-depleted cells .. had no significant effect in control non-depleted cells." but the BAF IF in the bottom left panel looks quite different from the BAF IF in the top right panel. Please explain. Also the lacZ spread here is pretty terrible and it's really hard to tell whether or not there is actually overlap between the CENP-C and BAF.
14. Middle of p. 8 and figure 4B,4C - Again, this part is very confusing. The order of the bars on the graph in 4B does not quite make sense to me, and I cannot tell if there are bars with 0% that cannot be seen. Perhaps it would be better to label the RNAi on the X axis and color code by 6d vs 3d.
15. It would be helpful if the RNAi protocol were a little better explained in the text – in the methods the authors describe sequential knockdowns or repeated RNAi treatments, and I think this needs to included in the main text where appropriate (for example, middle of p. 8 where MTS knockdowns are being done in Flfl depleted cells, etc).
16. The statistical analyses preformed need a bit more explanation in the methods section. Also, it is "Fisher's" not "Fischer".
17. P.10 – I am not convinced that cenBAF, CENP-C, and Flfl are interdependent for centromeric localization. Is there a way to show that CENP-C depletion affects the localization of both BAF and Flfl, and vise versa? Some nice IF is all that is needed.
18. The figure legend for figure 7 is extremely insufficient and even after having read the manuscript, I cannot understand what is being portrayed in this model.

Reviewer #2 (Remarks to the Author):

This manuscript by Torras-Llort et. al. describes a role for a sub-population of mitotic centromeric BAF in the regulation of the larger pool of non-chromatin associated mitotic BAF and in the proper assembly of CENP-C at centromeres and metaphase chromosome alignment. At the heart of this centromeric BAF is a proposed interaction of CENP-C, PP4 and BAF. The mis-regulation of mitotic BAF phosphorylation upon loss of centromeric BAF is reported to be due to aberrant activation of PP2A. Overall the experiments appear generally rigorous, perhaps with the exception of protein interaction studies and some of the imaging, and the data partially support the conclusions. There are some deficiencies in the design and/or interpretation of the co-IP studies as well as the colocalization of BAF with CENP-C and the localization of BAF in the absence of PP4. Until more clearly resolved these deficiencies limit the support for the proposed model/conclusions. Given that the relatively well studied BAF has never been seen before at mitotic centromeres, it would be worth demonstrating that this occurs in another well-studied system such as mammalian cells to ensure that the behavior is not species specific and to add rigor to the studies by showing the result is not specific to the utilized antibody.

Specific comments:

The localization of BAF, as detected with anti-BAF in figure 1A during interphase is strikingly unusual and not what has been shown for interphase BAF in other studies. Even given the striking genome organization of S2 cells compared to mammalian cells, there are not reports I am aware of for BAF being enriched so dramatically in HP1a foci of pericentromeric/telomeric heterochromatin. Was the custom anti-BAF used in this manuscript raised against the drosophila BAF, information on this was missing from the relevant section of the methods?

All Western blots need MW markers and loading controls where appropriate.

Why would FLAG-BAF3A bind only at centromeres and not all over the chromatin during mitosis as has been previously reported.

Figure2A. Could another histone protein be shown to show that there is specificity of loss of CENC-C from chromatin in the absence of BAF.

The BAF-YFP does not appear to colocalize well with the CENP-C in Figure S2E-F. There is some overlap in the signal, but it does not appear to predominantly overlap. Given the resolution of light microscopy this would not suggest direct interaction, or even localization in the same protein complex.

The co-IP experiments are not convincing of a BAF-CENP-C association. Both proteins bind to double stranded DNA and are likely being co-IP'd via that very indirect mechanism. Similar issues would have also impacted the original proteomic analysis of CENP-A -based affinity purification. Could the authors also probe for other DNA-binding and BAF-binding proteins in Figure 1D to show if there is any specificity of the CENP-C interaction.

Blots in Figure S3 D and E are difficult to interpret.

Can the authors show an immunoblot to accompany the results of Figure 4A. Levels of BAF in the CENP-C-FIM expressing cells appear quite elevated compared to the other conditions shown. It is also unclear what the BAF is colocalizing with as it doesn't appear on the chromosomes themselves or clearly concentrated in the chromosomal surface. It almost seems like there is simply more BAF and it is found in the cytoplasm, not on the chromatin.

Is there any evidence of A-type lamins or LEM domain proteins at the centromeres?

Figure S8A: Please show higher magnification images, and show colocalization with a centromeric marker.

POINT-BY-POINT RESPONSE TO REVIEWERS

Reviewer #1

General comment

The manuscript entitled, "Centromeric control of nuclear envelope dynamics" by Torras-Llort et al. utilizes cytological methods combined with basic biochemical approaches to investigate the role of BAF in centromere assembly and mitotic progression. A previous study from this lab and others identified BAF as an interacting factor of the centromeric histone CENP-A. Here, the authors show that a small pool of BAF remains dephosphorylated during mitosis and localizes to centromeres (cenBAF), and this cenBAF is essential for CENP-C centromeric localization and normal mitotic progression. Using phoso-gel electrophoresis and Co-IP assays, the authors go on to show that cenBAF remains dephosphorylated during mitosis, and this phosphorylation status is regulated by the PP4 subunit Flfl through interaction with CENP-C. Deleting the Flfl-interacting motif of CENP-C (CENP-CΔFIM) disrupts cenBAF levels without disrupting total BAF levels, and reduces the interaction between BAF and CENP-C. The authors then show that the failure of cenBAF to localize to centromeres results in global BAF mislocalization during mitosis, and this mislocalization reflects impaired phosphorylation regulation. Finally, the authors use live imaging and standard IF to show that disrupting cenBAF localization using CENP-CΔFIM significantly increases the time between NEBD and anaphase onset, and leads to increased mitotic defects and abnormal nuclear morphology after mitosis.
The findings presented in this manuscript are very intriguing, and a better understanding of how centromeres influence nuclear envelope dynamics upon mitotic entry and exit is indeed of broad interest to both the centromere and nuclear organization fields. However, I feel that the title is misleading, as this study has in fact not revealed any novel information about how centromeres regulate nuclear envelope dynamics, as the title suggests. While BAF is indeed associated with the nuclear envelope during interphase, the fact that BAF is required for normal mitoses in Drosophila has previously been shown (see ref 17), and this study falls short of adding new insights to this aspect of this particular story. Additionally, the actual analyses of the nuclear envelope were minimal, and I found no data on "nuclear envelope dynamics". Despite these potential pitfalls, I still feel that there is novelty to the findings in this manuscript and that the link between BAF and CENP-C, and the mechanistic insights into this interaction, as well as the insights into how BAF affects mitotic progression and its role in centromere and kinetochore assembly – is very interesting and is highly relevant to many aspects of chromosome biology.
Therefore, it is my suggestion that the title be changed (see point 1 below), and extensive revisions to the manuscript and additional analyses of the

existing data are done prior to acceptance for publication in Communications Biology.

We thank the reviewer for the very careful and detailed review of our manuscript. We also appreciate the reviewer's comments on the general interest and novelty of our findings and their relevance to the field of chromosome biology. We found the points raised by the reviewer very helpful and, by addressing them, we certainly think that our manuscript strongly improved. In particular, we must agree that the title was a bit of an overinterpretation and, perhaps, other parts of the manuscript too. Thus, we have extensively rewritten several sections (Title, Abstract, and parts of the Results and Discussion) trying to avoid any overinterpretation. In addition, we have performed additional studies on the integrity of the NE in mitosis, as well as to address the rest of the reviewer's concerns (see below). We hope that these revisions would answer the reviewer's concerns.

Major concerns

1. As stated above, I do not think this is an appropriate title for this manuscript, as "nuclear envelope dynamics" were not actually measured or quantified at any point, in my opinion.

We agree with the reviewer. The title has been changed to better reflect the main findings of our work.

The pool of BAF being visualized and studied here is cenBAF, which is not associated with the nuclear envelope (as there is not NE during mitosis). The authors hypothesize that this cenBAF facilitates centromere association with the NE after mitosis to facilitate NER, but was it actually demonstrated that this cenBAF is essential for NER after mitosis? The mitotic delays found by live imaging were mostly after NEBD and before NER, and therefore seemingly independent of nuclear envelope dynamics (p.9). On p.13 of the manuscript in the discussion, the authors explicitly state that their data "suggest" a defect in NEBD.

Our live imaging results show that mitosis duration is increased and, though the time from AO to NER is affected, the strongest delay observed is from NEBD to AO. We interpreted these results as suggesting a defect in "NE dynamics", meaning the rearrangements that the NE undergoes in mitosis. To call this "NE dynamics" was probably inappropriate. Thus, in this revised version, we avoid using the term "NE dynamics" and, trying to explain all this in a more precise way, we have extensively rewritten the parts of the Results (page 11, second paragraph) and Discussion (page 14, last paragraph, and page 15, first paragraph) sections describing these results.

Nuclear envelope morphology defects do not equal nuclear envelope dynamics defects. Studies have shown that chromosome missegregation itself is sufficient to cause nuclear blebbing and micronuclei formation.

Again, the reviewer is right on this matter. Altered nuclear morphology could be caused by multiple factors. But, importantly, constitutive targeting of BAF to centromeres rescued the observed morphology defects, indicating that they are

linked to cenBAF disruption. In this revised version we explicitly stated all this and the corresponding parts of the Results (page 12, first paragraph) and Discussion (page 15, last part in first paragraph) sections have been extensively rewritten.

One way to directly address the dynamics of the nuclear envelope would be to better analyze the live imaging with the RFP-Nup107 (or even better, an RFP-Lamin construct) to see if indeed there are defects in nuclear envelope dynamics before or after mitosis when the BAF pathway is defective.

*Indeed, we often observed persistent Nup-107::mRFP signal during mitosis (**Supplementary Movies S2-S4**), suggesting that NE disassembly was compromised. Nuclear pore disassembly is a very early step in NEBD. Thus, to further analyze the effects of disrupting cenBAF localization on NE assembly, we performed IF experiments using αPS10 antibodies, to identify mitotic cells, and αLaminB antibodies to monitor NE status. These experiments showed that, in comparison with control cells, the proportion of cells showing NE-assembled αLaminB immunostaining increased when cenBAF localization was disrupted. This increase was mainly observed in prophase, pointing to a defect in NEBD, but, though not statistically significant, we also observed an increase after AO. These new results are presented in **Figures 6c** and **6d**, and discussed in the Results (page 11, last paragraph and page 12, first two lines) and the Discussion (page 15, first paragraph, lines 12 to 14) sections.*

2. How many biological and/or technical replicates were done for each of these experiments? Ns are presented throughout the manuscript, but this seems to represent the number of cells analyzed for one replicate, and not how many replicates were performed.

We thank the reviewer for pointing out that this information was missing. In this revised version, for each experiment, we indicate the number of individual biological replicates performed in the corresponding Figure Legend.

3. On p. 7 of the text and figure 4A – how is this different from what is shown in figure 3A? On the right in 3A, we see the GFP-CENP-C∆FIM and BAF IF with control knockdown, which should look the same as middle of 4A, showing GFP-CENP-C∆FIM with BAF IF with no knockdown. Why is the perichromatin BAF only present in 4A? If this image was enhanced to emphasize this localization pattern, that's fine, but it needs to be indicated in the figure legend, and the original unenhanced image needs to be included in the supplemental data. If this image has NOT been enhanced, then again, why is there no perichromatin BAF in 3A?

*We thank the reviewer for raising this important point. Impairing centromeric localization of Flfl results in reduced cenBAF localization. However, significant accumulation of perichromosomal BAF was observed in only ~50% of the mitoses. Thus, a mitosis showing strong perichromosomal BAF accumulation was presented in **Figure 4a** (not enhanced), while, to better show the reduction of cenBAF in **Figure 3a**, we presented a mitosis without perichromosomal BAF accumulation to avoid any interference. In this regard, in this revised version, we have performed additional analyses and experiments. On one hand, we have*

*determined separately the extent of cenBAF reduction in mitoses that do or do not show perichromosomal BAF. We found that the accumulation of perichromosomal BAF correlates with the decrease of cenBAF. These new analysis is presented in **Fig. 4c**. In addition, we performed super-resolution microscopy experiments that confirmed the strong co-localization of cenBAF with CENP-C at centromeres in control cells and its strong reduction when centromeric Flfl localization was disrupted in CENP-C-depleted GFP::CENP-CΔFIM^R-expressing cells. These new results are presented in **Figs. 4d-4f**. Accordingly, we have extensively rewritten the corresponding part of the Results section (last paragraph in page 8, page 9 and first three lines in page 10).*

4. This whole section starting on p.7 and ending on p.9 describing the results in figure 4 ("cenBAF prevents dephos..") was nearly impossible to follow and needs to be simplified.

Following the reviewer's suggestions, this whole section has been extensively rewritten for simplicity and to include the new results discussed in previous point 3. We have also changed the subtitle of this section. Hopefully, we may have succeeded in making the presentation of the results easier to follow.

5. In the discussion section on p.11, the "not shown" in vitro pull down data need to be shown and further discussed – how can the authors say that it is possible for BAF and Flfl to directly interact, when the experimental data for this interaction suggest otherwise?

*For the in vitro pull-down experiments, we used GST-constructs carrying each of the two substrate-binding domains of Flfl, EVH1 and SMK1. None of the two constructs were able to pull-down ^{35}S-BAF expressed in vitro using coupled transcription-translation (IVTT) (see **Fig. 1** below). Although these experiments failed to detect a direct Flfl-BAF interaction, we cannot completely rule out the possibility that the interaction occurs in vivo. In fact, in vitro pull-down experiments have some important limitations. On one hand, the proteins used in these experiments are missing any specific post-translational modification that could play a critical role in mediating the interaction. In addition, the interaction could be facilitated by another factor(s) that was missing in the reaction (for instance CENP-C). In the absence of this partner, the interaction could be too transient to be detected by GST-pull down experiments. Therefore, we think that more experiments need to be done to study Flfl-BAF interaction. Given all these limitations, we feel more comfortable by removing the sentence discussing these results and, accordingly, we have rewritten this part of the Discussion section (page 13, second paragraph).*

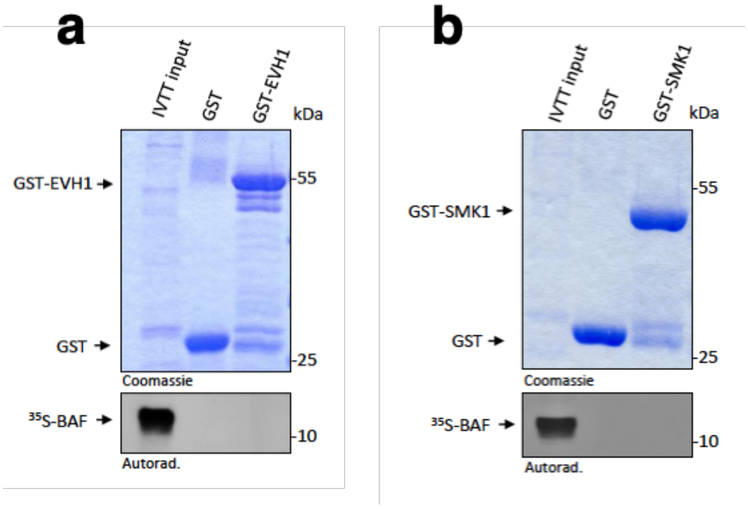

***Figure 1**. Recombinant GST, GST-EVH1 (**a**) or GST-SMK1 (**b**) were incubated with in vitro expressed (IVTT) ^{35}S-methionine-labelled BAF, purified and run on SDS-PAGE. Coomassie-stained gel shows the bait proteins while autoradiography shows ^{35}S-BAF.*

6. Bottom of p.9, Figure 6 – The authors make several inferences here that are not entirely supported by the data, or at least are not the only possible explanation for the findings. Specifically, the result that these mitotic defects lead to increased ploidy – how did the authors come to this conclusion? Because of increased number of CENP-C foci? This could also be the result of defects in centromere clustering since S2 cells are already highly polyploid, and disrupted centromere clustering is known to happen in the subsequent interphase after defective chromosome segregation. Is ploidy inferred from nuclear volume? Again, this could also be the result of global chromatin decompaction. The reasoning behind these inferences is unclear and again, alternative possibilities need to be discussed. Directly testing ploidy would require doing mitotic chromosome spreads to show increased chromosome number (interphase DNA FISH would not be helpful in this case, as somatic homolog pairing in flies makes ploidy differences undetectable during interphase). Additionally, as described above, the defects in nuclear envelope morphology described here could all be an indirect result of general mitotic errors rather than a direct consequence of cenBAF disruption. This needs to be discussed.

We agree with the reviewer that to conclude that disrupting cenBAF localization results in cells with increased ploidy due to a defect in NE dynamics was a bit of an overinterpretation of the results. Additionally, whether cells with large irregular nuclei have increased ploidy or not is not a main point in our story. Even more since, as the reviewer pointed out, S2 cells already have altered ploidy and defective NE morphology could arise from several different causes. Therefore, in this revised version, we mild down our initial claims and we have extensively rewritten the part of the Results section describing the effects on NE morphology (page 12, first paragraph). As mentioned above, we have also rewritten the

corresponding part of the Discussion section (page 15, last part in first paragraph).

Minor concerns
7. General issues with the figures.
- no statistical analyses included in the graphs of the figures or the figure legends, only the main text. Asterisks indicating significance should be added to the graphs.
In this revised version, we have included all the data on the statistical tests used in the Figure Legends and asterisks are shown in the Figures themselves.
- Graphs should all have their own letter assignment and not be "grouped" with the associated IF so that the figures are easier to follow and references made to figures in the text are clearer (for example, sometimes it is unclear if the authors are referencing an image or a graph if they are both designed 4A*).*
We have relabeled all the Figures according to the reviewer's suggestion.
- The red/green coloring of all images is problematic for any colorblind readers. I suggest switching to magenta/cyan if possible.
We apologize for this and we will take into consideration this sensitive suggestion in future works.

8. Figure 2E – in the text (p.5) these data are further broken down into metaphase alignment defects and anaphase segregation defects. These data should be included in the main figure here, as the mechanisms behind these two types of mitotic defects are typically distinct. For example, metaphase misalignment is typically the result of defective kinetochore/microtubule attachments, while segregation errors in anaphase can happen for many reasons, including chromosome compaction defects and other non-centromeric defects. The fact that both of these types of defects are seen in the BAF knockdown is significant, and is important to support the authors' model for a role of BAF in centromere/kinetochore assembly.
We thank the reviewer for this suggestion. In this revised version, we have included in ***Fig. 2e*** *the data on chromosome segregation defects broken down into total, metaphase misalignment and anaphase bridges+lagging chromosomes.*

9. Figure 3B – the anti-BAF on this blot is very dirty and it's nearly impossible to see the bands. Do you have a cleaner blot you can include instead? The same is true for Figure S3D.
We have improved the quality of the image in Fig. 3c so that the bands corresponding to BAF are more visible and Supplementary Fig. S3d has been removed since the information it contained was redundant with that in Supplementary Fig. S3e (in this revised version ***Supplementary Fig. S5e****).*

10. Figure 3C – why is the DAPI so saturated here? It's really hard to see where/if the IF is colocalized in the merged panel. The same is true for Figure S7B.

We have replaced/improved these images to show less saturated DAPI signals. **Figs. 3e** and **3f** correspond to previous **Fig. 3c**, and **Fig. 8e** corresponds to previous **Fig. S7b.**

11. P. 7 and figure 4A – does the GBP-FLAG-BAF construct affect alone affect mitotic progression or centromere assembly?

*To address this question, we have performed IF experiments to determine the effects of GBP::FLAG::BAF expression in control GFP::CENP-C^R-expressing cells. We have determined the centromeric levels of CENP-A^{CID} (**Supplementary Figs. S9c** and **S9d**), the incidence of chromosome segregation defects (**Supplementary Figs. S9e** and **S9f**) and the proportion of cells showing altered nuclear morphology (**Supplementary Figs. S9g** and **S9h**). In all these analyses, we found that expression of GBP::FLAG::BAF had no detectable effects. These results are presented in the Results section (page 9, third paragraph, lines 6 and 7).*

12. P. 7,8, figure 4A,B – why do you think that this "perichromosomal" BAF is ectopically associated with chromatin? Wouldn't it co-localize with DAPI if it were chromatin bound? To me this seems like it could be more broadly nucleoplasmic.

*We thank the reviewer for raising this point. To address this question, we have performed super-resolution microscopy analysis (**Fig. 4d-4g**). These studies showed that perichromosomal BAF partially overlaps with the DAPI signal, but the maximum intensity is observed beyond the chromosome surface in the "nucleoplasm". It is possible that this reflects polymerization of BAF around chromatin as observed at the initial steps of NER when BAF forms a dense network around the decondensing chromosomes. However, we cannot unambiguously tell if perichromosomal BAF is actually bound to the chromosomes, we can simply say that it is associated with them. Therefore, in this revised version of the manuscript, we avoid talking about "binding of perichromosomal BAF to chromosomes" and we have extensively rewritten the part of the Results section describing these results (page 8, last paragraph and page 9), and the subtitle of the corresponding section has been changed. Interestingly, these new super-resolution studies also confirmed both the strong colocalization of cenBAF with CENP-C at centromeres in control cells (**Figs. 4d and 4e**) and the reduced cenBAF levels when centromeric Flfl localization is impaired (**Figs. 4f and 4g**).*

13. P. 8 and figure 4B – the authors state that, "MTS depletion in Flfl-depleted cells .. had no significant effect in control non-depleted cells." but the BAF IF in the bottom left panel looks quite different from the BAF IF in the top right panel. Please explain. Also the lacZ spread here is pretty terrible and it's really hard to tell whether or not there is actually overlap between the CENP-C and BAF.

*We agree with the reviewer that, in general, the images presented in previous **Fig. 4b** were not of the highest quality. Therefore, in this revised version, we present improved images to show accumulation of perichromosomal BAF in Flfl-*

*depleted cells (**Fig. 5a, top right panel**) but not in MTS-depleted cells (**Fig. 5a, bottom left panel**), which is what we actually meant. We also show that depletion of MTS in Flfl-depleted cells abolishes the accumulation of perichromosomal BAF (**Fig. 5a, bottom right panel**) and the images of the dsRNA^{lacZ} control have also been improved (**Fig. 5a, top left panel**). Hopefully, we may have succeeded in explaining these results in a clearer way in this revised version (page 10, second paragraph).*

14. Middle of p. 8 and figure 4B,4C - Again, this part is very confusing. The order of the bars on the graph in 4B does not quite make sense to me, and I cannot tell if there are bars with 0% that cannot be seen. Perhaps it would be better to label the RNAi on the X axis and color code by 6d vs 3d.
*Following the reviewer's suggestions, we have changed the order of the graphs in previous **Fig. 4b** (**Fig. 5b** in this revision version) and we have rewritten this part of the Results section (page 10, last two paragraphs).*

15. It would be helpful if the RNAi protocol were a little better explained in the text – in the methods the authors describe sequential knockdowns or repeated RNAi treatments, and I think this needs to included in the main text where appropriate (for example, middle of p. 8 where MTS knockdowns are being done in Flfl depleted cells, etc).
As the reviewer suggested, we have explained in some more detail the RNAi experiments both in the Methods (page 18, last paragraph and page 19, first paragraph) and Results (page 10, lines 11 to 15 in the second paragraph) sections.

16. The statistical analyses preformed need a bit more explanation in the methods section. Also, it is "Fisher's" not "Fischer".
We have added in the Methods section a subsection on statistical analysis (page 21, last paragraph and page 22, first paragraph)

17. P.10 – I am not convinced that cenBAF, CENP-C, and Flfl are interdependent for centromeric localization. Is there a way to show that CENP-C depletion affects the localization of both BAF and Flfl, and vise versa? Some nice IF is all that is needed.
*In this revised version we present these IF data, which were scattered through various Figures in the previous version, in a single main **Fig. 8**, which shows the effects of CENP-C depletion on both centromeric Flfl (**Fig. 8d**) and cenBAF (**Fig. 8e**), as well as the effects of Flfl-depletion on CENP-C levels (**Fig. 8b**) and BAF-depletion on both centromeric CENP-C and Flfl levels (**Fig. 8c**). These results fully support the idea that cenBAF, Flfl and CENP-C are interdependent for their centromeric localization. We have also extensively rewritten for simplicity and clarity the part of the Results section describing these results (page 12, last paragraph).*

18. The figure legend for figure 7 is extremely insufficient and even after having read the manuscript, I cannot understand what is being portrayed in this model.
*We have extended legend to Figure 7 (**Figure 9** in this revised version) to explain in more detail the model discussed in the text (page 14, second paragraph).*

Reviewer #2

General comments

This manuscript by Torras-Llort et. al. describes a role for a sub-population of mitotic centromeric BAF in the regulation of the larger pool of non-chromatin associated mitotic BAF and in the proper assembly of CENP-C at centromeres and metaphase chromosome alignment. At the heart of this centromeric BAF is a proposed interaction of CENP-C, PP4 and BAF. The mis-regulation of mitotic BAF phosphorylation upon loss of centromeric BAF is reported to be due to aberrant activation of PP2A. Overall the experiments appear generally rigorous, perhaps with the exception of protein interaction studies and some of the imaging, and the data partially support the conclusions. There are some deficiencies in the design and/or interpretation of the co-IP studies as well as the colocalization of BAF with CENP-C and the localization of BAF in the absence of PP4. Until more clearly resolved these deficiencies limit the support for the proposed model/conclusions.

We thank the reviewer for the very careful and detailed review of our manuscript. We found the points raised by the reviewer very helpful and, by addressing them, we certainly think that our manuscript strongly improved. To address the main questions raised by the reviewer, we have performed a number of new experiments and analyses as detailed below. In particular, we have performed new localization experiments both in Drosophila and mammalian cells and additional co-IP experiments have been carried out (please see below and the answers to specific comments). We hope that these revisions would answer the reviewer's concerns.

Given that the relatively well studied BAF has never been seen before at mitotic centromeres, it would be worth demonstrating that this occurs in another well-studied system such as mammalian cells to ensure that the behavior is not species specific and to add rigor to the studies by showing the result is not specific to the utilized antibody.

[redacted]

[redacted]

[redacted].

Specific comments:

The localization of BAF, as detected with anti-BAF in figure 1A during interphase is strikingly unusual and not what has been shown for interphase BAF in other studies. Even given the striking genome organization of S2 cells compared to mammalian cells, there are not reports I am aware of for BAF being enriched so dramatically in HP1a foci of pericentromeric/telomeric heterochromatin. Was the custom anti-BAF used

in this manuscript raised against the drosophila BAF, information on this was missing from the relevant section of the methods?

*We thank the reviewer for pointing out that this information was missing. The αBAF antibody used in these experiments was raised against full length Drosophila BAF. This information has been added to the Methods section (page 17, first three of lines). Specificity of αBAF antibody was determined by both WB analysis (**Supplementary Fig. S1a**) and IF experiments in mitotic (**Supplementary Figs. S1b** and **S1d**) and interphase cells (**Supplementary Figs. S1c** and **S1d**). Moreover, to further analyze the localization of BAF in interphase, we performed IF experiments in stable S2 cells expressing BAF::YFP, which was detected by direct fluorescence. The pattern of localization of this construct largely mimicked immunostaining with αBAF, showing strong overlap with HP1a (**Supplementary Fig. S2a**). These new results are presented in the Results section (page 4, lines 14 to 16 in the first paragraph).*

All Western blots need MW markers and loading controls where appropriate.

Following the reviewer's suggestion, we have added MW markers to all WBs, except for the phos-tag WBs since, in these gels, proteins do not run according to their MW and, moreover, loading pre-stained markers perturbs electrophoretic migration in the adjacent lanes.

Why would FLAG-BAF3A bind only at centromeres and not all over the chromatin during mitosis as has been previously reported.

*We agree with the reviewer that these experiments were not accurately described. In fact, the phosphonull FLAG::BAF^{3A} mutant showed persistent binding across chromosomes in mitosis (**Fig. 3e, left**), but ~15% of the mitoses (N= 123) showed localization only at centromeres (**Fig. 3f, right**). These results are consistent with non-phosphorylated BAF binding chromatin and, on the other hand, with cenBAF being non-phosphorylated. We cannot unambiguously tell why FLAG::BAF^{3A} localizes exclusively at centromeres only in a relatively low percentage of mitoses, but we think that variable levels of expression might account for this. In cells expressing high levels of FLAG::BAF^{3A}, it binds across chromosomes, while, in cells showing lower levels of expression, FLAG::BAF^{3A} localizes only at centromeres in mitotic chromosomes. In this regard, it must be noted that, in these experiments, the various constructs were transiently expressed since we were not able to generate stable lines with the phosphonull FLAG::BAF^{3A} mutant. The part of the Results section describing these results has been extensively rewritten for accuracy and clarity (page 8, second paragraph). Hopefully, in this revised version, we may have succeeded in explaining these results in a better way.*

Figure2A. Could another histone protein be shown to show that there is specificity of loss of CENC-C from chromatin in the absence of BAF.

*This is a difficult question to address since the only centromere specific histone is CENP-A^{CID} that, as shown by our results, was also reduced upon disrupting cenBAF localization (**Figs. 2a-2c** and **Supplementary Fig. S4a** and **S4b**). However, CENP-A^{CID} was reduced to a much lower extent than CENP-C (**Fig. 2c***

*and **Supplementary Fig. S4b**). Moreover, when the dominant negative BAF::YFP mutant is expressed, CENP-C levels were strongly reduced without significantly affecting CENP-A^{CID} levels (**Supplementary Figs. S4c** and **S4d**). On the other hand, considering that BAF is known to affect histone modifications and higher-order chromatin organization, it is possible that cenBAF has a general stabilizing effect on centromeric chromatin. In this regard, cenBAF also appears to stabilize centromeric Flfl independently of CENP-C, as shown by the recruitment of Flfl when BAF is constitutively targeted to centromeres in CENP-C-depleted GFP::CENP-CΔFIM^{R}-expressing cells (**Supplementary Figs. S6a** and **S6b**).*

***The BAF-YFP does not appear to colocalize well with the CENP-C in Figure S2E-F. There is some overlap in the signal, but it does not appear to predominantly overlap. Given the resolution of light microscopy this would not suggest direct interaction, or even localization in the same protein complex**.*

*The reviewer is right in this matter, the localization of BAF::YFP in mitotic chromosomes often extended to pericentromeric heterochromatin. In this revised version, we have included enlarged images of several examples, ranging from centromere only to strong pericentromeric localization (**Supplementary Fig. 2c**). We have addressed the reasons behind this observation, finding that BAF::YFP has an impaired pattern of phosphorylation (**Supplementary Fig. S5b**) that likely accounts for its persistent binding to heterochromatin in mitosis.*

***The co-IP experiments are not convincing of a BAF-CENP-C association. Both proteins bind to double stranded DNA and are likely being co-IP'd via that very indirect mechanism. Similar issues would have also impacted the original proteomic analysis of CENP-A -based affinity purification. Could the authors also probe for other DNA-binding and BAF-binding proteins in Figure 1D to show if there is any specificity of the CENP-C interaction**.*

*To address this question, we have performed two sets of experiments. First, we have tested whether the co-IP was resistant to treatment of the extract with DNAse I prior to immunoprecipitation, finding that it was (**Supplementary Figs. S3a** and **S3b**). And second, we checked whether BAF co-immunoprecipitates with the centromeric H3 variant CENP-A^{CID}. For these experiments, we used a stable S2 cell line expressing a CENP-A^{CID}::YFP fusion protein that showed strong centromeric localization (**Supplementary Fig. S3c**) since, in our hands, endogenous CENP-A^{CID} was difficult to detect by WB in co-IP experiments. We observed that αBAF antibodies did not co-immunoprecipitate CENP-A^{CID}::YFP (**Supplementary Fig. S3d, lane 4**) and, similarly, neither BAF nor CENP-C could be co-immunoprecipitated with αGFP (**Supplementary Fig. S3c, lane 3**). These results strongly suggest that the co-IP of BAF with CENP-C is, on one hand, not mediated by chromatin binding and, on the other hand, specific. However, we do not think that BAF is interacting with CENP-C in the nucleoplasm. What we think is happening is that BAF and CENP-C are interacting in the centromere and, during preparation of the extract in mild salt conditions, they are partially solubilized. Of course, CENP-A^{CID} is not easily solubilized under these conditions since it forms part of the centromeric nucleosomal particles and, thus, it is tightly*

bound to chromatin. In this revised version, these new experiments are described in the Results section (page 5, second paragraph).

Blots in Figure S3 D and E are difficult to interpret.

*We agree that the way we presented these results was a bit confusing. In this revised version, we have deleted Supplementary Fig. S3d since the information it contained was redundant to that in Supplementary Fig. S3e (in this revised version **Supplementary Fig. S5e**). These results showed specificity of αBAFpS5 antibodies since, in phos-tag gel electrophoresis analysis, they recognized pBAF, but not non-phosphorylated BAF (**Supplementary Fig. S5e**, **first two lanes in right panel**), and a S5A mutation abolished this reactivity (**Supplementary Fig. S5e, last two lanes in right panel**). In this revised version, these results are presented in the Methods section (page 17, lines 7 to 12 in first paragraph).*

Can the authors show an immunoblot to accompany the results of Figure 4A. Levels of BAF in the CENP-C-FIM expressing cells appear quite elevated compared to the other conditions shown. It is also unclear what the BAF is colocalizing with as it doesn't appear on the chromosomes themselves or clearly concentrated in the chromosomal surface. It almost seems like there is simply more BAF and it is found in the cytoplasm, not on the chromatin.

*In this revised version, we present results from WB analysis showing that total BAF levels are not significantly increased when cenBAF localization is disrupted in CENP-C-depleted GFP::CENP-CΔFIM^R-expressing cells (**Supplementary Fig. S8**) (see first three lines in page 9). In addition, we have performed super-resolution microscopy analysis (**Fig. 4d-4g**). These studies showed that perichromosomal BAF partially overlaps with the DAPI signal, but the maximum intensity is observed beyond the chromosome surface in the "nucleoplasm". It is possible that this reflects polymerization of BAF around chromatin as observed at the initial steps of NER when BAF forms a dense network around the decondensing chromosomes. However, we cannot unambiguously tell if perichromosomal BAF is actually bound to the chromosomes, we can simply say that it is associated with them. Therefore, in this revised, we avoid talking about "binding of perichromosomal BAF to chromosomes" and we have extensively rewritten the part of the Results describing these results (page 8, last paragraph and page 9), and the subtitle of the corresponding section has been changed, as well. Interestingly, these super-resolution studies confirmed both the strong colocalization of cenBAF with CENP-C at centromeres in control cells (**Figs. 4d and 4e**) and the reduced cenBAF levels when centromeric Flfl localization is impaired (**Figs. 4f and 4g**).*

Is there any evidence of A-type lamins or LEM domain proteins at the centromeres?

To the best of our knowledge, there is no much information in the literature about the binding of LEM-domain proteins to centromeres, except for results in S. pombe (which does not contain BAF) showing that LEM2 binds centromeres and is required for their tethering to the NE and heterochromatin silencing (Barrales et al., (2016) Genes Dev 30:133-48). [redacted]

[redacted]

[redacted]

[redacted]

Figure S8A: Please show higher magnification images, and show colocalization with a centromeric marker.

*Following the reviewer's suggestion, we present higher magnification images of the co-localization of the CENP-A^{CID}::TAP construct with CENP-C in both interphase cells (**Supplementary Fig. S12a**) and mitotic chromosomes (**Supplementary Fig. S12b**).*

REVIEWERS' COMMENTS:

Reviewer #1 (Remarks to the Author):

The quality of the paper has greatly improved with the additional co-IP and imaging experiments. Additionally, some of new/clarified figures and the more appropriate title have eased many of my major concerns about this manuscript. Combined, the data is overall convincing, and the paper represents a significant advance in our understanding of BAF's function in chromosome segregation and role in centromere maintenance. Yet, there are still portions of the text that are unclear and the manuscript would benefit from being simplified in places. Therefore, I do recommend this paper for publication in Communications Biology with several suggestions:

1) The BAF-YFP construct used in supplemental figure 2B-2D is later determined to be a dominant negative mutant, so using it as a proof of principle for BAF antibody specificity doesn't make sense. What about showing the FLAG-BAF construct used later instead? Otherwise I would suggest removing this first discussion of the YFP-BAF and only introduce it later as a dominant negative mutant.
2) Also, p.4-p.6, any speculation on why the YFP tag leads to loss-of-function? Is it because of the phosphorylation defects mentioned (meaning, is the abnormal phosphorylation a cause or consequence of abnormal YFP-BAF function)? Are both centromere and non-centromere BAF functions affected? This section would benefit from some more explanation of this mutant and overall more coherent explanation of the phenotypes.
3) p.5 – result that CENP-C did not co-IP with YFP-CID - similar results has been shown before and the reference should be included (Mellone et al., 2011, PLoS Gen).
4) I still feel that zoomed images/insets would make it easier for the reader to see the centromeric (or non-centromeric) localization of proteins, specifically on the mitotic spreads in Figures 2a, 2b, 4a, 5a, and 5c.
5) Throughout the text, conclusions and interpretation of data need to be more clearly spelled out. The authors are relying on readers to make their own conclusions and sometimes the logic is unclear. For example, p.6-p.11 – Is the conclusion that CENP-C FIM mutants don't recruit cenBAF to centromeres, or don't protect cenBAF from phosphorylation? Either seems feasible. This is nicely spelled out on p. 12 ("Results reported above suggest a model by which CENP-C mediates recruitment of PP4/Flfl to centromeres, PP4/Flfl retains cenBAF at centromeres in mitosis, which in turn stabilizes CENP-C." This needs to be included earlier and similar statements are needed throughout the text.
6) Figure 6C – can the authors comment on whether the persistent lamin staining in mitosis corresponds to the location of the mitotic spindle and what this could possibly mean?
7) Also Figure 6C, CENP-C dsRNA, metaphase panel – how is there GFP-CENP-C staining where there is no PH3/chromatin staining? Is there DAPI at these regions?
8) p.15 – the authors' state, "Altered nuclear morphology is widely associated with generic mitotic problems. However, the defects observed upon impairing cenBAF localization are rescued by constitutive targeting of BAF to centromeres, indicating that they are directly linked to cenBAF disruption." Since tethering BAF to centromeres also rescues CENP-C (and therefore CENP-A) recruitment, I do not know if the authors can truly determine if the nuclear morphology defects are directly due to cenBAF dysfunction.
[redacted]

Reviewer #2 (Remarks to the Author):

This revised manuscript by Torras-Llort et. al. describing the functional association of BAF with centromeres has addressed all of my concerns raised from my first review. I would certainly like to see eventual follow-up of these finding in mammalian cells as well as exploration of the presence and function of other NE proteins such as LEM domain proteins, which would also suggest the presence of membranes, at mitotic centromeres.

POINT-BY-POINT RESPONSE TO REVIEWERS

Reviewer #1

We thank the reviewer for the positive comments on the revised version of our manuscript and the recommendation for publication.

The reviewer makes several suggestions to improve the paper:

1) The BAF-YFP construct used in supplemental figure 2B-2D is later determined to be a dominant negative mutant, so using it as a proof of principle for BAF antibody specificity doesn't make sense. What about showing the FLAG-BAF construct used later instead? Otherwise I would suggest removing this first discussion of the YFP-BAF and only introduce it later as a dominant negative mutant.
Following the reviewer's suggestion, we have moved the part describing the pattern of localization of the BAF::YFP construct to the section presenting the results on the dominant negative nature of BAF::YFP (page 5, third paragraph, lines 9-16).

2) Also, p.4-p.6, any speculation on why the YFP tag leads to loss-of-function? Is it because of the phosphorylation defects mentioned (meaning, is the abnormal phosphorylation a cause or consequence of abnormal YFP-BAF function)? Are both centromere and non-centromere BAF functions affected? This section would benefit from some more explanation of this mutant and overall more coherent explanation of the phenotypes.
Our results suggest that the dominant negative character of BAF::YFP is likely associated with destabilization and replacement of endogenous BAF by a non-functional BAF::YFP form of altered chromatin binding dynamics due to abnormal phosphorylation. This is now explicitly discussed in the Results section (page 6, first paragraph, lines 11-18), together with the description of the data supporting the hypothesis.

3) p.5 – result that CENP-C did not co-IP with YFP-CID - similar results has been shown before and the reference should be included (Mellone et al., 2011, PLoS Gen).
We thank the reviewer for pointing out that similar results were reported earlier by Mellone et al. We have included a sentence in the section describing the co-IP results and Mellone's paper is cited (page 5, last sentence in the first paragraph).

4) I still feel that zoomed images/insets would make it easier for the reader to see the centromeric (or non-centromeric) localization of proteins, specifically on the mitotic spreads in Figures 2a, 2b, 4a, 5a, and 5c.
We think that the mentioned Figures are already quite crowded to include additional images. On the other hand, readers could easily zoom-in to visualize these images to a higher magnification.

5) Throughout the text, conclusions and interpretation of data need to be more clearly spelled out. The authors are relying on readers to make their own conclusions and sometimes the logic is unclear. For example, p.6-p.11 – Is the conclusion that CENP-C FIM mutants don't recruit cenBAF to centromeres, or don't protect cenBAF from phosphorylation? Either seems feasible. This is nicely spelled out on p. 12 ("Results reported above suggest a model by which CENP-C mediates recruitment of PP4/Flfl to centromeres, PP4/Flfl retains cenBAF at centromeres in mitosis, which in turn stabilizes CENP-C." This needs to be included earlier and similar statements are needed throughout the text.

Following the reviewer suggestion, we have included several of such statements throughout the text (page 5, second paragraph; page 7, second paragraph; page 8, last sentence in the first paragraph; page 10, first paragraph, lines 1-5; page 12, last sentence in the first paragraph). We hope this will make easier for the reader to follow our interpretation of the results.

6) Figure 6C – can the authors comment on whether the persistent lamin staining in mitosis corresponds to the location of the mitotic spindle and what this could possibly mean?

In these experiments, we did not visualize the spindle. Therefore, we cannot tell much about this interesting question.

7) Also Figure 6C, CENP-C dsRNA, metaphase panel – how is there GFP-CENP-C staining where there is no PH3/chromatin staining? Is there DAPI at these regions?

Disrupting centromeric localization of PP4 (and consequently of cenBAF) results in CENP-C delocalization to centrosomes and across the spindle. This phenotype, which was already reported in (Lipinszki et al., 2015, Nature Commun), is observed in metaphase chromosomes, suggesting that tension exerted by spindle microtubules is somehow involved in CENP-C delocalization. All this is explicitly discussed in the Discussion section (page 14, second paragraph, lines 9-17). We thank the reviewer for pointing out this observation.

8) p.15 – the authors' state, "Altered nuclear morphology is widely associated with generic mitotic problems. However, the defects observed upon impairing cenBAF localization are rescued by constitutive targeting of BAF to centromeres, indicating that they are directly linked to cenBAF disruption." Since tethering BAF to centromeres also rescues CENP-C (and therefore CENP-A) recruitment, I do not know if the authors can truly determine if the nuclear morphology defects are directly due to cenBAF dysfunction.

We agree with the reviewer that, considering the interdependences between BAF, PP4 and CENP-C (as well as of CENP-C and CENP-A^{CID}), we cannot unambiguously say that the effect on nuclear morphology of constitutive targeting of BAF to centromeres is directly due to BAF. Therefore, we have removed the word "directly" from the sentence discussing these results (page 16, first paragraph, line 4).

[redacted]

Reviewer #2

The reviewer did not raise any additional questions. We thank the reviewer for the positive comments.
